# Differentiated demographic histories and local adaptations between Sherpas and Tibetans

**DOI:** 10.1186/s13059-017-1242-y

**Published:** 2017-06-15

**Authors:** Chao Zhang, Yan Lu, Qidi Feng, Xiaoji Wang, Haiyi Lou, Jiaojiao Liu, Zhilin Ning, Kai Yuan, Yuchen Wang, Ying Zhou, Lian Deng, Lijun Liu, Yajun Yang, Shilin Li, Lifeng Ma, Zhiying Zhang, Li Jin, Bing Su, Longli Kang, Shuhua Xu

**Affiliations:** 1 Chinese Academy of Sciences (CAS) Key Laboratory of Computational Biology, Max Planck Independent Research Group on Population Genomics, CAS-MPG Partner Institute for Computational Biology (PICB), Shanghai Institutes for Biological Sciences, CAS, Shanghai, 200031 China; 2 University of Chinese Academy of Sciences, Beijing, 100049 China; 3 School of Life Science and Technology, ShanghaiTech University, Shanghai, 201210 China; 4 Key Laboratory for Molecular Genetic Mechanisms and Intervention Research on High Altitude Disease of Tibet Autonomous Region, School of Medicine, Xizang Minzu University, Xianyang, Shaanxi 712082 China; 5 State Key Laboratory of Genetic Engineering and Ministry of Education (MOE) Key Laboratory of Contemporary Anthropology, School of Life Sciences, Fudan University, Shanghai, 200433 China; 6 State Key Laboratory of Genetic Resources and Evolution, Kunming Institute of Zoology, Chinese Academy of Sciences, Kunming, 650223 China; 7 Collaborative Innovation Center of Genetics and Development, Shanghai, 200438 China

**Keywords:** Sherpa, Tibetan, Next-generation sequencing, High-altitude adaptation, Population history, Gene flow

## Abstract

**Background:**

The genetic relationships reported by recent studies between Sherpas and Tibetans are controversial. To gain insights into the population history and the genetic basis of high-altitude adaptation of the two groups, we analyzed genome-wide data in 111 Sherpas (Tibet and Nepal) and 177 Tibetans (Tibet and Qinghai), together with available data from present-day human populations.

**Results:**

Sherpas and Tibetans show considerable genetic differences and can be distinguished as two distinct groups, even though the divergence between them (~3200–11,300 years ago) is much later than that between Han Chinese and either of the two groups (~6200–16,000 years ago). Sub-population structures exist in both Sherpas and Tibetans, corresponding to geographical or linguistic groups. Differentiation of genetic variants between Sherpas and Tibetans associated with adaptation to either high-altitude or ultraviolet radiation were identified and validated by genotyping additional Sherpa and Tibetan samples.

**Conclusions:**

Our analyses indicate that both Sherpas and Tibetans are admixed populations, but the findings do not support the previous hypothesis that Tibetans derive their ancestry from Sherpas and Han Chinese. Compared to Tibetans, Sherpas show higher levels of South Asian ancestry, while Tibetans show higher levels of East Asian and Central Asian/Siberian ancestry. We propose a new model to elucidate the differentiated demographic histories and local adaptations of Sherpas and Tibetans.

**Electronic supplementary material:**

The online version of this article (doi:10.1186/s13059-017-1242-y) contains supplementary material, which is available to authorized users.

## Background

Living in the Qinghai-Tibet Plateau with an average elevation of over 4500 m, the Sherpas and Tibetans were some of the most mysterious populations until Tenzing Norgay, a Sherpa, conquered Mount Everest in the middle of the 20th century and attracted the attention of anthropologists, archaeologists, and geneticists. Both highlander groups seem to cope well with the tremendously hypoxic environment and possess a distinctive set of adaptive physiological traits, including unelevated hemoglobin concentrations even up to 4000 m, which is clearly associated with oxygen delivery [1–5]. Many genetic studies have attributed these adaptive traits to variants in *EPAS1* (MIM 603349) and *EGLN1* (MIM 606425), two key genes in the hypoxia inducible factor (HIF) pathway that detect and react to oxygen supply changes [1, 6–9]. The adaptation to high altitude suggests these groups have occupied the region for a long time. Archaeological evidence suggests the first people arrived at the Tibetan plateau as early as 30,000 years ago [10]. By collecting 6109 Tibetan samples and conducting phylogeographic analyses using paternal, maternal, and genome-wide autosomal markers, Qi et al. revealed the presence of both Upper Paleolithic (40–10 thousand years ago [ka]) colonization and Neolithic (10–4 ka) expansion of modern humans on the Tibetan plateau [11], while Yi et al. suggested the divergence period between the highlanders and the Han Chinese, a lowland population, was only 2750 years [6]. In a recent study, we provided compelling evidence of the co-existence of Paleolithic and Neolithic ancestries in the modern Tibetan gene pool through whole-genome sequencing, and thus indicated a genetic continuity between pre-historical highland-foragers and present-day Tibetans and Sherpas [12].

However, the Neolithic population history and the genetic relationships between Sherpas and Tibetans remain controversial. It is mostly conceded that Sherpas were originally Tibetans who migrated from eastern Tibet to the Everest region of Nepal 500 years ago according to their similarity in Tibeto-Burman languages, adherence to Tibetan Buddhism sects, oral legends, and other traditions [13, 14]. The absence of a written history of the Sherpa people makes their origins much more legendary [13–15]. Recent genetic evidence has led to conflicting conclusions when elucidating the genetic relationships of the two highlander populations. Based on autosomal genomes, Jeong et al. posited that modern Tibetans were a mixture of ancestral populations related to the Sherpa and Han Chinese, and consequently their genetic adaptations to high altitudes were likely inherited from the ancestral Sherpa [16]. Conversely, two recent studies based on mtDNA and Y-chromosomal data reported that the Sherpa people are a recently derived sub-lineage of Tibetans, dated to less than 1500 years ago, suggesting that Sherpas likely acquired high-altitude adaptive features during their ancestors’ long stay on the Tibetan Plateau prior to their most recent migration towards Nepal [17, 18]. These contrasting views may have resulted from different genetic material, investigative methods, or interpretations, which indicates the complex genetic admixture origins of the Sherpa and Tibetan people.

Additionally, much less is known about geographic and cultural roles in shaping the population substructures within both Tibetans and Sherpas. Since Tibetans reside in different regions surrounding high transverse valleys, complex terrain may have hindered communication between subgroups. Moreover, gene flow in different Tibetan ethnic groups is entirely unexplored despite there being three cultural regions of historical Tibet [19] (Ü-Tsang, Kham, and Amdo Tibet). On the other hand, the Sherpa people primarily reside in the Khumbu region of Nepal with smaller groups in Dingjie County and Zhangmu Town [18], along the Sino-Nepalese border in the Tibet Autonomous Region of China. Furthermore, Khumbu Sherpas consider themselves as distinct from both other Sherpas and non-Sherpa peoples [13–15], suggesting a more complex history of Sherpa populations. Whether the genetic makeup of Khumbu Sherpas is distinct from Sherpas residing in Tibet and whether genetic contact between Sherpa subgroups occurred remain to be elucidated.

**Fig. 1:**
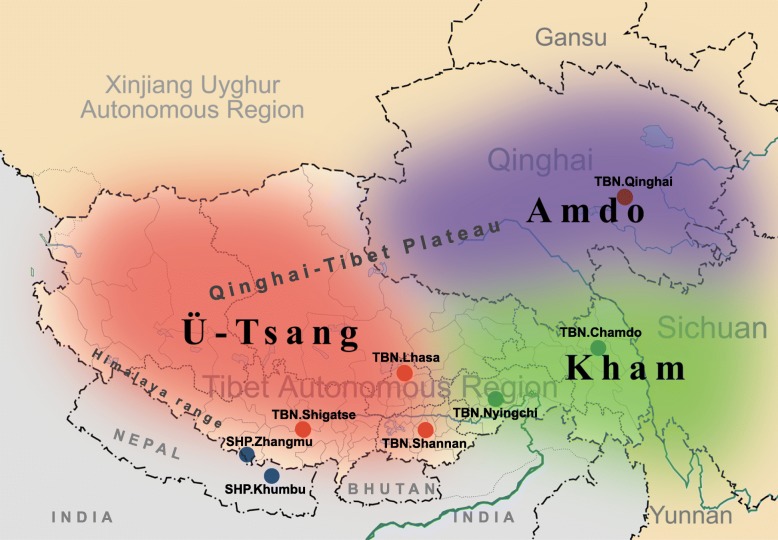
Culturally defined regions of historical Tibet and geographical locations of Sherpa and Tibetan samples analyzed in this study. The culturally defined regions in historical Tibet are illustrated in different colors: *red*, Ü-Tsang (central Tibet); *green*, Kham (eastern Tibet); and *purple*, Amdo (northeastern Tibet). *Dots with distinct colors* represent subgroups classified according to the collected geographical locations: *blue* for two SHP subpopulations; and *non-blue* for regional Tibetans. The locations of Amdo and Kham regions make Tibetans there more easily influenced by cultures and genetics from East Asians or Central Asians/Siberians. The figure was modified from one obtained from Wikipedia (https://en.wikipedia.org/wiki/Kham)

Existing archaeological and genetic data are insufficient to directly resolve the complex relationship between and within the two highlander populations. Therefore, we used whole-genome deep sequencing and genome-wide genotyping data from Sherpas, Tibetans, and the Han Chinese (Fig. 1; Additional file 1: Table S1) to revisit and address four major unresolved issues regarding their prehistory, especially the Neolithic history of Sherpas and Tibetans, and their hypoxic adaptation: (i) whether they are two genetically different ethnic groups; (ii) whether population substructures exist in either of the two groups; (iii) how long they have diverged from their ancestral group and when the two separated groups started to re-contact by population admixture; and (iv) whether the two groups share major high-altitude adaptation mechanisms. The careful and systematic analysis of these newly sequenced genomes, together with available genotyping data, can provide further insight into the genetic origins of Sherpas and Tibetans and uncover their different adaptive mechanisms.

## Results

### Sherpas and Tibetans are two genetically distinct groups

Genetic relationships between Sherpas (SHP) and Tibetans (TBN) in the context of 203 contemporary worldwide populations (Additional file 1: Figure S1), measured by unbiased *F*_ST_ (Additional file 1: Figures S2–S4) and outgroup *f*_3_ tests (Additional file 1: Figures S6–S7) show that the two highlander populations’ closest affinity is to East Asian populations, and the second closest is to Central Asian/Siberian populations. The overall genetic makeup of SHP is closest to TBN (*F*_ST_ = 0.007), followed by surrounding populations living on the Tibet Plateau, such as Tu (*F*_ST_ = 0.012), Yizu (*F*_ST_ = 0.013), and Naxi (*F*_ST_ = 0.016), which possibly results from a direct shared ancestry or reciprocal gene flow between these populations (Additional file 1: Figures S3 and S7). Although South Asian populations are located geographically near SHP, the genetic differences are much larger between SHP and South Asians than between SHP and East Asians, indicating a gene flow barrier between East Asia and the South Asian subcontinent. These relationships were roughly consistent when analyzing TBN (Additional file 1: Figure S3 and S7), but with some differences. Although SHP share the greatest number of alleles with TBN, TBN’s nearest affinity was not with SHP (*F*_ST_ = 0.007) but with populations such as Tu (*F*_ST_ = 0.005) and Yizu (*F*_ST_ = 0.006) (Additional file 1: Figure S3). This pattern was also confirmed by outgroup *f*_3_ tests (Additional file 1: Figure S7), suggesting different demographic histories of SHP and TBN following a population split. Furthermore, Sherpa from Khumbu of Nepal (SHP.Khumbu) were closest to Sherpa from Zhangmu County of China (SHP.Zhangmu), while SHP.Zhangmu showed nearest genetic affinities with some Tibetan subgroups, particularly TBN.Shigatse (Additional file 1: Figures S4 and S8). These results indicate that SHP.Zhangmu might have had genetic contact with Tibetans following population divergence.

**Fig. 2:**
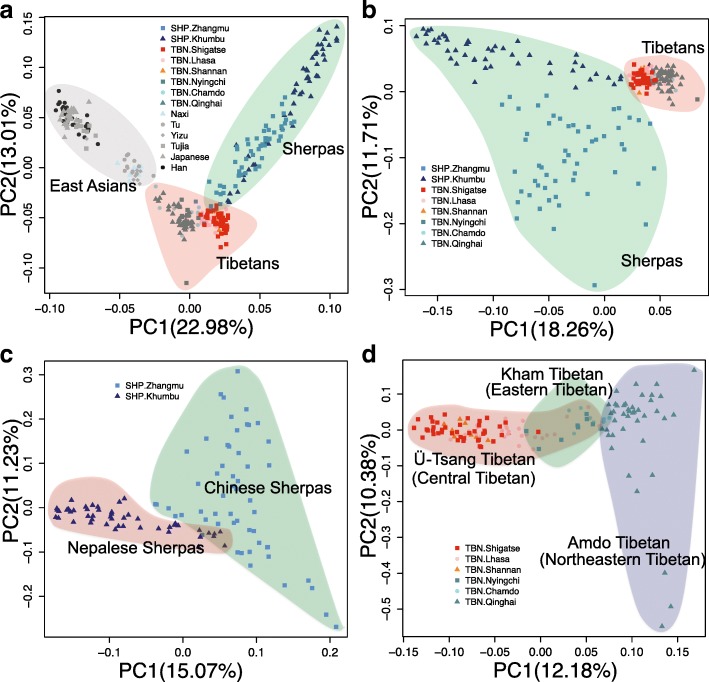
Principal component analysis (PCA) for SHP, TBN, and their subgroups. PCA of **a** SHP and TBN within the context of some East Asians, **b** SHP and TBN, **c** SHP subgroups, and **d** TBN subgroups. Subgroups are classified according to their geographic locations. *Numbers in parentheses* denote variance explained by each principal component (*PC*). Note that three outliers in **d** (one in TBN.Nyingchi and two in TBN.Shigatse) were removed when we drew the figure

Principal component analysis (PCA) positions SHP and TBN in clusters surrounded by a majority of East Asian populations and a small number of Central Asian/Siberian and South Asians populations (Fig. 2a; Additional file 1: Figures S11 and S12). SHP and TBN were separated into two different subclusters in the two-dimensional PC plot, either before or after removing a series of worldwide populations (Fig. 2b; Additional file 1: Figures S11 and S12), suggesting they are two distinct groups rather than a homogenous population as previously thought [13–15]. Interestingly, population substructures were observed in both SHP and TBN when we grouped individuals according to their geographical locations (Fig. 2c, d). Tibetans were clustered more tightly whereas Sherpas were much more scattered. SHP.Zhangmu and SHP.Khumbu were split by PC1 (Fig. 2c). It is unlikely that a batch effect from different genotype platforms accounts for the substructure of the two regional SHP subgroups since microarray data from both platforms were consistent with whole-genome sequencing data for the replicated samples (see “Methods”; Additional file 1: Figure S13). Meanwhile, the TBN subgroups were generally clustered into three clades: one for groups from Ü-Tsang Tibetan (TBN.Shigatse, TBN.Lhasa, and TBN.Shannan), one for that from Kham Tibetan (TBN.Chamdo and TBN.Nyingchi), and another for Amdo Tibetan (TBN.Qinghai) (Fig. 2b). These patterns were also revealed by *F*_ST_ (Additional file 1: Figure S5) and outgroup *f*_3_ (Additional file 1: Figures S9 and S10) analysis. We assorted Tibetan individuals according to the literature resources [7, 9, 20] and ruled out the possibility that population structures were induced by batch effects (Additional file 1: Figure S14).

Lastly, analysis of molecular variance (AMOVA) was performed. When assuming SHP and TBN as two distinct groups, the results show that although the majority (99%) of the variance was sourced from the within-population level, the among-group variance is significant (*P* ≤ 0.001) and larger than the variance among populations within groups (Additional file 1: Table S2). However, when assorting either one of the TBN subgroups (TBN.Shigatse, TBN.Lhasa, TBN.Shannan, TBN.Nyingchi, TBN.Chamdo, or TBN.Qinghai) with the SHP (SHP.Zhangmu and SHP.Khumbu), the variance among populations within groups significantly exceeded the among-group variance (*P* < 0.001) (Additional file 1: Table S2), confirming that SHP and TBN are two genetically distinct populations.

### Admixture history of Sherpas and Tibetans

**Fig. 3:**
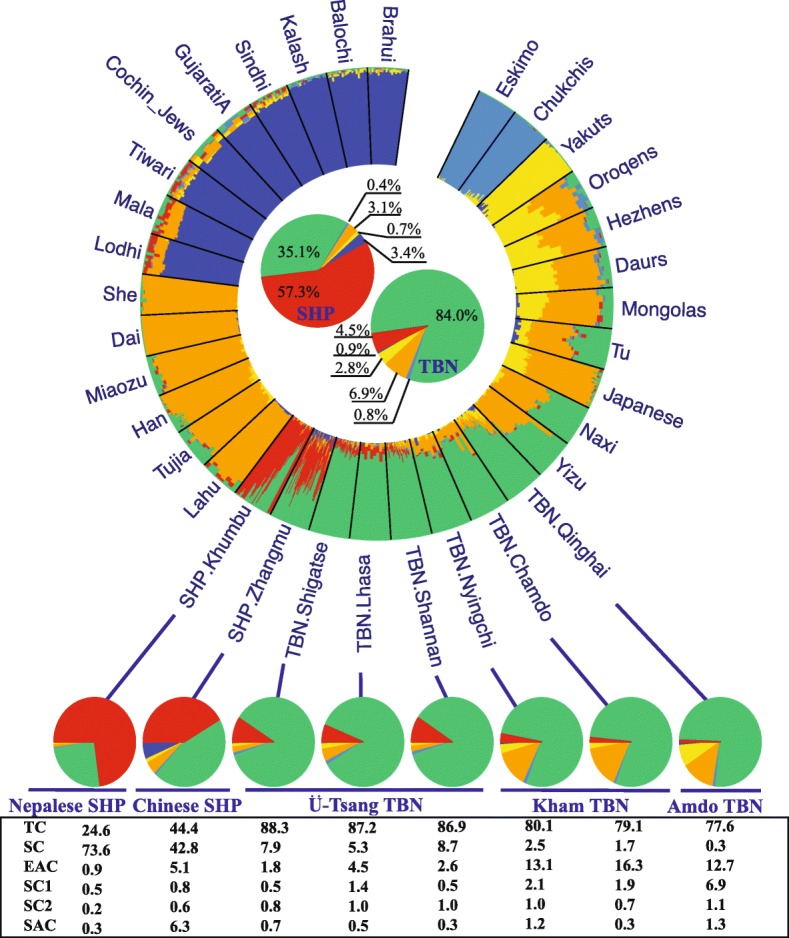
Panel 2 dataset-based results of genetic admixture when assuming *K* = 6. Each individual is represented by a single line broken into *K* = 6 colored segments, with lengths proportional to the *K* = 6 inferred clusters. Results for all SHP and TBN are further summarized and displayed in the two large pie charts in the center of the *circle plot* with component proportion denoted as percentage. Proportions of each genetic component for SHP and TBN subgroups are summarized in the small pie charts with their proportions listed below. *TC* Tibetan major component, *SC* SHP major component, *EAC* East Asian major component, *SC1* Central Asian/Siberian major component 1, *SC2* Central Asian/Siberian major component 1, *SAC* South Asian major component

To dissect the genetic components of SHP and TBN, we conducted *ADMIXTURE* analysis using the surrounding populations (Additional file 1: Figures S15 and S16) and a panel consisting of nine South Asian populations (Lodhi, Sind, Tiwari, Mala, Cochin Jews, Gujarati, Brahui, Balochi, and Kalash), three Central Asian/Siberian populations (Yakuts, Chukchis, and Eskimo), and 13 East Asian populations (She, Dai, Miaozu, Han, Japanese, Tu, Tujia, Lahu, Yizu, Naxi, Mongolas, Daurs, and Hezhens) as applied by Jeong et al. [16] (Additional file 1: Figures S17 and S18). This made our results comparable to those of Jeong et al. [16]. We estimated 4, 5, and 6 as the best numbers of ancestral populations (*Ks*) based on the estimation of cross-validation (CV) error (Additional file 1: Figure S19) and observed that larger *Ks* did not change the genetic components for most of populations. Assuming *K* = 4 or 5, we found SHP and TBN shared genetic components with some East Asians, especially Yizu, Naxi, and Tu. However, in each scenario with *K* > 5 we observed a SHP-specific component that was in low frequency in TBN, illustrating SHP’s distinct demographic history from TBN after splitting from their common ancestor. Furthermore, SHP showed, on average, more South Asian ancestry (3.5 ± 4.8%) than TBN (0.8 ± 1.5%) when assuming *K* = 6 (Fig. 3; Additional file 1: Figure S20). On the other hand, the East Asian component (EAC) and Central Asian/Siberian component (CSC) in TBN (6.9 ± 6.8% and 2.8 ± 3%, respectively) were much higher than that in SHP (3.0 ± 3.8% and 0.6 ± 1%, respectively), suggesting greater genetic influences from East Asians and Central Asians/Siberians in TBN.

Additionally, substructures within both TBN and SHP were consistent with our PCA (Fig. 2). Although residing South of the Himalayas, the Nepalese Sherpas (SHP.Khumbu) have a smaller inferred ancestry component from the dark blue cluster, predominantly assigned to populations from South Asia (0.3 ± 1.2%; Fig. 3), than the Chinese Sherpas (SHP.Zhangmu) (6.2 ± 5.0%). One possible explanation is that Zhangmu town is a port of entry on the Nepal–Tibet border with an average elevation of 2300 m, a much lower altitude than Khumbu in Nepal (3800 m), and therefore facilitated gene flow from East and South Asia to SHP.Zhangmu. Furthermore, the SHP.Khumbu have been more isolated than SHP.Zhangmu, which is supported by their much longer run of homozygosity (ROH) compared to Chinese Sherpas (Wilcoxon’s test, *P* < 0.01) (Additional file 1: Figure S21). Within Tibetans, more SHP-enriched ancestry (7.3 ± 2.7%) was observed in Ü-Tsang Tibetans, including TBN.Shigatse (7.9 ± 2.6%), TBN.Lhasa (5.3 ± 3.0%), and TBN.Shannan (8.7 ± 2.4%), than in Kham and Amdo Tibetans (0.8 ± 1.4%), including TBN.Nyingchi (2.5 ± 2.4%), TBN.Chamdo (1.7 ± 1.7%), and TBN.Qinghai (0.3 ± 0.7%). This suggests that more gene flow occurred from SHP to Ü-Tsang TBN, and from East Asians into East Tibetans (Kham Tibetan and Amdo Tibetan). Lastly, a greater Central Asian/Siberian component was observed in TBN.Qinghai than in any other Tibetan subgroup.

**Fig. 4:**
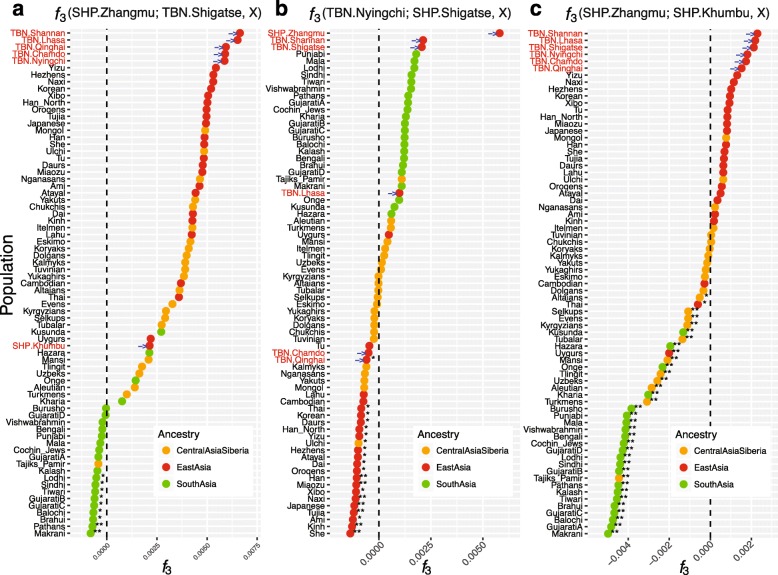
Evidence of gene flow between SHP and TBN subgroups. We performed *f*_3_ tests to detect gene flow events from the TBN subgroup to SHP subgroup (Additional file 1: Figure S23), SHP subgroup to TBN subgroup (Additional file 1: Figure S24), and within SHP subgroups (Additional file 1: Figure S25). The *f*_3_ statistics were significantly negative (with Z score ≤3) for: **a** *f*_3_(SHP.Zhangmu; TBN.Shigatse, X) when X was assumed as a South Asian population; **b** *f*_3_(TBN.Nyingchi; SHP.Khumbu, X) when X is an East Asian population; and **c** *f*_3_(SHP.Zhangmu; SHP.Khumbu, X) when X was South Asians and some Central Asians/Siberians. Results provide evidence for gene flow from South Asians and Nepalese Sherpas to Chinese Sherpas, and from East Asians and Nepalese Sherpas to Tibetans in Nyingchi. **Significantly negative value with Z scores ≤3; *score of 3 < Z ≤ 2. Highlander subgroups are highlighted with *red fonts* and *blue arrows*

To further test presence of gene flow, we performed three*-*population tests following Raghavan et al. [21]. Firstly, we detected the admixture signals in TBN when it was treated as one single population. By using *f*_*3*_(TBN; SHP, X), we found significantly negative scores where X represented some East Asian population, such as Yizu, Oreqens, or Naxi (Additional file 1: Figure S22a), possibly indicating the target population (TBN) was admixed between SHP and X. In contrast, little gene flow was detected with SHP as the target population with *f*_*3*_(SHP; TBN, X) (Additional file 1: Figure S22b). However, the high degree of population-specific drift in Nepalese SHP could have resulted in non-significantly negative *f*_*3*_(SHP; TBN, X). Secondly, we detected gene flow between subgroups of SHP and TBN. We identified gene flow from TBN.Shigatse and some South Asians into SHP.Zhangmu by testing *f*_*3*_(SHP.Zhangmu; TBN.Shigatse, X) (Fig. 4a; Additional file 1: Figure S23a). In contrast, no significant negative values were observed when SHP.Khumbu was the target in testing *f*_*3*_(SHP.Khumbu; TBN.Subgroup, X) (Additional file 1: Figure S23b) [22]. Meanwhile, gene flow from SHP.Khumbu and some East Asians, such as Naxi, into TBN.Nyingchi was also detected (Fig. 4b; Additional file 1: Figure S24). Strong gene flow event(s) from South Asians occurred in SHP.Zhangmu (SHP.Zhangmu; SHP.Khumbu, X), where SHP.Khumbu was assumed the reference/ancestral population of SHP.Zhangmu (Fig. 4c; Additional file 1: Figure S25). These results suggest the gene flow from both East and South Asians to SHP.Zhangmu would be much more frequent than that to SHP.Khumbu as the latter was more isolated and shows much longer ROH (Additional file 1: Figure S21).

As revealed by *ADMIXTURE* analysis, SHP samples are genetically heterogeneous (Fig. 3) and such high variation is suggestive of recent rather than ancient admixture, otherwise a uniform distribution of ancestry components across individuals is expected. We selected 16 proxies from SHP.Khumbu with their SHP-specific component larger than 97% (according to *ADMIXTURE* analysis) to represent ancient SHP (hereafter referred to as SHPproxy) to detect the recent admixture signals for non-SHPproxy (Additional file 1: Table S3). On one hand, we found East Asians, Central Asians/Siberians, and South Asians contributed genetic ancestry to SHP with significantly negative *f*_*3*_ (SHP; SHPproxy, X) scores, where SHP represents non-SHPproxy individuals from both Khumbu and Zhangmu (Additional file 1: Figure S26a). Since the *f*_3_ test is model-based and relies on referenced populations, we detected whether the gene flow from East Asians and Central Asians/Siberians was indirect and introduced via gene flow from TBN, which received ancestry from those populations. We found that East Asian and Central Asian/Siberian ancestry estimated by *ADMIXTURE* was significantly positively correlated with the estimated Tibetan ancestry (with R^2^ = 0.45 and *p* = 1.4 × 10^−6^, and R^2^ = 0.22 and *p* = 0.026, respectively) across Sherpa individuals, supporting the speculation that SHP received East Asian and Central Asian/Siberian ancestry indirectly via gene flow from Tibetans. On the other hand, assuming TBN as the recipient of gene flow with *f*_*3*_(TBN; SHPproxy, X) (Additional file 1: Figure S26b), we further confirmed that TBN received gene flow from some East Asians and Central Asians/Siberians instead of South Asians. The results are also supported by *TreeMix* [23], which indicated gene flow from South Asians into SHP and from East Asians and Central Asians/Siberians into the common ancestor of TBN and SHP (Additional file 1: Figures S31 and 32).

Lastly, to compare the relative ancestry contribution from the reference populations to SHP and TBN, we applied *f*_4_(SHP, TBN; Yoruba, X), where negative *f*_4_ values suggest excess sharing of SHP alleles and positive scores indicate more shared alleles with TBN (Additional file 1: Figure S27). Overall, when setting X as South Asians, the *f*_4_ values tended to be negative, indicating that populations from South of the Himalayas, such as Balochi and Brahui, have closer genetic affinities with SHP than with TBN. Meanwhile, with always positive *f*_4_ scores, East Asian and Central Asian/Siberian populations shared more alleles with TBN than with SHP, illustrating more genetic influence by their geographically eastern neighbors. These results are in agreement with *ADMIXTURE* and show that SHP harbors greater South Asian ancestry compared to TBN, indicating more gene flow from South Himalayan populations. We then applied *f*_4_ tests to detect population substructures within SHP (SHP.Zhangmu, SHP.Khumbu; Yoruba, X) (Additional file 1: Figure S28) and TBN (TBN.Subgroup1, TBN.Subgroup2; Yoruba, X) (Additional file 1: Figures S29 and 30). Consistent with our PCA (Fig. 2), *ADMIXTURE* (Fig. 3), *F*_ST_ (Additional file 1: Figures S4 and S5), and outgroup *f*_3_ tests (Additional file 1: Figures S8–10), TBN.Shigatse, TBN.Shannan, and TBN.Lhasa tended to share more alleles, while TBN.Nyingchi, TBN.Chamdo, and TBN.Qinghai showed close genetic affinities. These results further support the existence of population substructures among Ü-Tsang, Kham, and Amdo Tibetans, and are consistent with culturally defined regions of historical Tibet [19] (Fig. 1).

### Sherpa and Tibetan Paleolithic and Neolithic demographic history

**Fig. 5:**
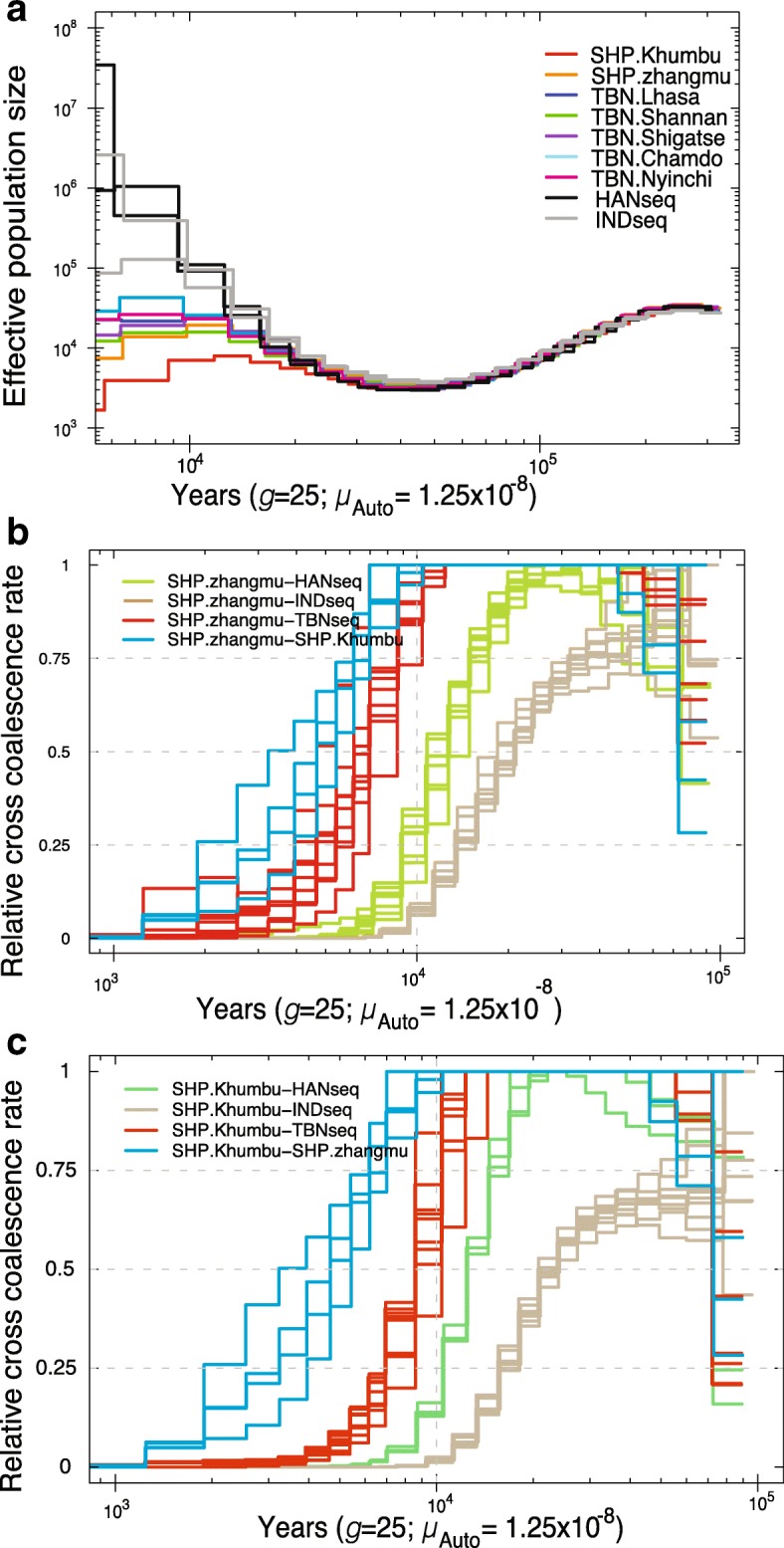
The historical effective population size (*N*_e_) and divergence time between SHP and TBN. Estimates of **a** *N*_e_ and divergence time between **b** SHP.Zhangmu and others and **c** SHP.Khumbu and others using MSMC. The *N*_e_ was estimated using autosomal sequences of two genomes (four haplotypes) for each population. Divergence time between each pair of populations was evaluated using autosomal sequences of four genomes, i.e., two individuals for each population. An autosomal mutation rate (*μ*_Auto_) with 1.25 × 10^−8^ per base-pair per generation and 25 years per generations (*g*) were used

We sequenced the genomes of five Chinese Sherpas, 33 Tibetans and 39 Han Chinese to high coverage (>30×) [12]. Two Nepalese Sherpa [16] and seven Indian [24] genomes were also included to comprise a next-generation sequencing (NGS) panel (see “Methods”; Table 1), which was used to infer the historical effective population size (*N*_e_) and divergence time using multiple sequentially Markovian coalescent (MSMC) analysis [25]. The Nepalese Sherpa had a small *N*_e_ (Fig. 5a) since ~30,000 years ago, which is also consistent with estimates from previous studies [16] and results obtained from the linkage disequilibrium (LD)-based method (Additional file 1: Figure S33). Meanwhile, the *N*_e_ of Chinese Sherpa was relatively larger than that of Nepalese Sherpa and lower than that of Tibetan subgroups, Indian, and Han Chinese. Both Sherpa groups, especially Nepalese Sherpa, experienced bottleneck events 8000–9000 years ago (320–360 generations ago) (Fig. 5a), at which time the Han Chinese underwent continual Neolithic population expansion. We speculate that the decreased population size of Nepalese Sherpa resulted from the dispersion of Han agriculturalists around 10,000 years ago [11, 26–28]. Compared with Nepalese Sherpa, Tibetans showed a slightly increasing population size during that time (Fig. 5a; Additional file 1: Figure S33), indicating gene flow from outside the Tibet Plateau into Tibetans, but not into Sherpas, beginning in the early Neolithic, 10,000–7000 years ago [11].

**Table 1:** Summary of population samples and data used in this study

Population	Number of samples	Number passing QC	Platform	Collected region	Altitude (m)	Source	Symbol	Panel
Tibetan	31	31	Affy 6.0	Qinghai (31)	~4350	Simonson et al. [20]	TBN.Qinghai (42) TBN.Shigatse (43) TBN.Lhasa (30) TBN.Shannan (9) TBN.Nyingchi (9) TBN.Chamdo (9)	1, 2
Tibetan	50	49	Affy 6.0	Lhasa (20), Shigatse (18), Qinghai (11)	>3000	Peng et al. [7]		
Tibetan	69	64	Affy 6.0	Lhasa (10), Chamdo (9), Nyingchi (9), Shannan (9) and Shigatse (25)	>3000	Xu et al. [9] and newly generated in this study		
Sherpa	61	55	Affy 6.0	Zhangmu Town, Shigatse (55)	~3400	This study	SHP.Zhangmu (55)	1, 2
Sherpa	2	2	NGS	Solo-Khumbu region, Nepal (2)	~3800	Jeong et al. [16]	SHP.Khumbu (2) (SHPseq2 in NGS panel)	1, NGS panel
Sherpa	69	49	Illumina HO-Q	Solo-Khumbu region, Nepal (49)	~3800	Jeong et al. [16]	SHP.Khumbu (49)	2
Sherpa	5	5	NGS	Zhangmu Town, Tibet. (5)	~3400	Lu et al. [12]	SHPseq (5)	NGS panel
Tibetan	33	33	NGS	Lhasa (3), Chamdo (6), Nagqu (3), Nyingchi (2), Shannan (7), and Shigatse (12)	>3000	Lu et al. [12]	TBNseq (33)	NGS panel
HAN Chinese	39	39	NGS	Diverse region in China (39)	<2500	Lu et al. [12]	HANseq (39)	NGS panel
Indian	7	7	NGS	Diverse region in South Asia	<2500	Chambers et al. [24]	IND	NGS panel
203 worldwide populations	2345	2345	Affy HumanOri	Worldwide regions (2345)	-	Patterson et al. [22]	Followed the original paper	1, 2
Tibetan	118	118	SNaPshot	Six prefectures in Tibet	>3000	This study	-	Target-genotyping panel
Sherpa	78	78	SNaPshot	Zhangmu Town, Tibet	~3400	This study	-	Target-genotyping panel

Included are both our newly generated genomes and other previously published samples. We assigned four different panels for distinct investigations: panels 1 and 2 comprised SNP array data, except the Nepalese Sherpas; the NGS panel contained enrolled NGS genomes; and the Target-genotyping panel was used to validate allele frequencies of interesting SNPs by enlarging size. Subgroup symbols are classified according to their geographical locations (see also Fig. 1). Numbers in brackets are the counts of individuals after quality control with proportion of identity by descant (IBD) smaller than 3.5 and individual SNP missing rate less than 0.1. *Abbreviations*: *Affy 6.0* Affymetrix Genome-wide Human SNP Array 6.0, *Illumina HO-Q* Illumina HumanOmni1-Quad beadchip, *NGS* next-generation sequencing, *Affy HumanOri* Affymetrix Axiom Genome-wide Human Origins 1 array

In our recent work [12], we estimated that Tibetans diverged from Han Chinese ~15,000–9000 years (~600–360 generations) ago, much earlier than the estimate of 2750 years ago by a recent study based on exome sequencing data [6]. We also estimated that Chinese Sherpas shared ancestry with Tibetans ~11,000–7000 years (~440–280 generations) ago. These results indicated that the divergence between Sherpa and Tibetan populations was later than that between Han Chinese and either of the two groups. In a previous study, Jeong et al. suggested that that Nepalese Sherpas began to diverge from Han Chinese ~40,000 years ago and Tibetans are descendants of admixture of Han Chinese and ancestral Sherpas [16]. We analyzed the two individual Nepalese Sherpa genomes reported by Jeong et al. [16] together with genomes of Chinese Sherpas, Tibetans, and Han Chinese (Table 1) to address the discrepancies between the two studies. We estimated that the divergence time was ~1240–7800 years between Nepalese Sherpas and Chinese Sherpas and ~6100–13,300 years between Nepalese Sherpas and Tibetans, both slightly later than that between Nepalese Sherpas and Han Chinese (~9500–18,000 years ago) (Fig. 5; Additional file 1: Figure S34). Therefore, our analysis confirmed that the divergence of the gene pool of Nepalese Sherpas from that of Han Chinese was much less than 40,000 years ago.

To examine whether Tibetans are descendants of admixture of Han Chinese and ancestral Sherpas as Jeong et al. suggested, we further applied G-PhoCS [29], which considers gene flow in modeling population demographic history. Analysis of the same data sets using G-PhoCS gave a divergence time of ~5100 years between Sherpas and Tibetans, and ~6100 years between Han Chinese and both highlander groups (Additional file 1: Table S5), which were also consistent with the estimates of divergence time (*T*_*F*_) based on *F*_ST_ and *N*_e_ [30] (Additional file 1: Table S4). Despite these estimations (based on G-PhoCS and *T*_*F*_) being smaller than those based on MSMC analysis, the overall relationships among the three groups were consistent with that suggested by MSMC analysis, i.e., the divergence between Sherpas and Tibetans was later than that between Han Chinese and either of the two groups. Therefore, our findings do not support the previous hypothesis that Tibetans derive their ancestry from Sherpas and Han Chinese.

**Fig. 6:**
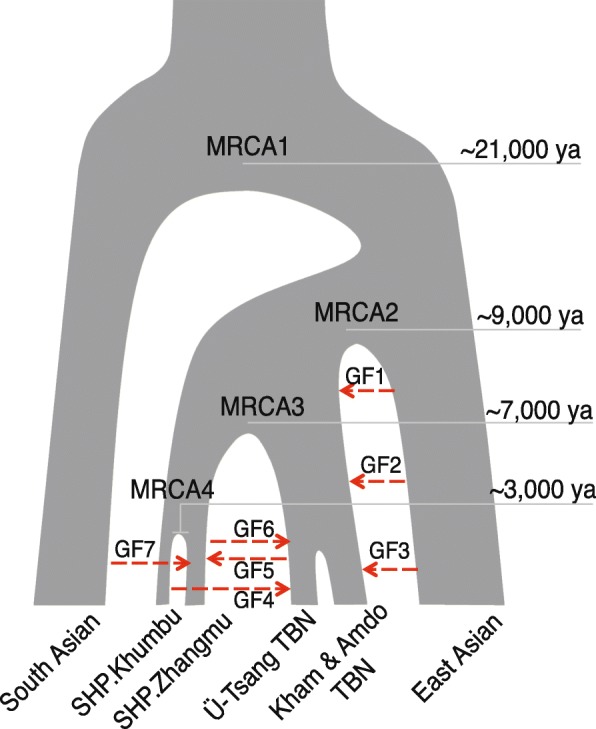
A proposed model of demographic history of SHP and TBN. A simplified model for the origins and evolutionary history of Tibetans and Sherpas based on the observations and estimations from this study. *GF* gene flow, *MRCA* most recent common ancestor. *Dashed lines* indicate gene flow events and *arrows* denote directions. *MRCA1*, *MRCA2*, and *MRCA3* are based on Fig. 5b. We inferred GF1 from the *treemix* results (Additional file 1: Figures S31 and S32) and the observation that both SHP (mainly for Chinese Sherpa) and TBN contain an East Asian genetic component (EAC) (Fig. 3). GF2 was based on the excess EAC in TBN compared to SHP (Fig. 3; Additional file 1: Figure S27). Based on the *f*_3_ tests (Fig. 4b; Additional file 1: Figure S22) and the higher proportion of EAC in Kham and Amdo Tibetans (Fig. 3), we confirmed GF3. GF4 is based on Fig. 4b and Additional file 1: Figure S23 and the historical record that Sherpas migrated from the Kham region in eastern Tibet to Nepal within the last 300–400 years, possibly supporting the genetic contact between Khumbu Sherpas and Kham Tibetans. GF5 is based on the excess Sherpa genetic component in Ü-Tsang Tibetans compared to that in Kham and Amdo Tibetans (Fig. 3) and also on the results shown in Additional file 1: Figure S26. GF6 is based on Fig. 4a. The higher South Asian component in Chinese Sherpas compared to that in Nepalese Sherpas (Fig. 3) and the *f*_3_ statistics (Fig. 4a) validated the presence of GF7. Population substructures in both SHP and TBN are based on PCA (Fig. 2), *ADMIXTURE* (Fig. 3), *F*_ST_ (Additional file 1: Figures S4 and S5), outgroup *f*_3_ tests (Additional file 1: Figures S8–S10), and D statistics (Additional file 1: Figures S28–S30). Estimates of MRCA1, MRCA2, and MRCA3 are based on Fig. 5b and Additional file 1: S34

Taken together, we propose a simplified model (Fig. 6) to describe the Paleolithic and Neolithic demographic history of both Sherpas and Tibetans. Recent gene flow from South Himalayan populations into Sherpas, East Asian genetic contribution to modern Tibetans, and the disparate contact between Sherpas, Tibetans, and their subgroups were collectively responsible for the genetic diversification in the two highlander populations.

### Shared and differential altitudinal adaptation between Sherpas and Tibetans

Previous studies reported some common adaptation mechanisms between Tibetan and Sherpa, involving genes such as *EPAS1* and *EGLN1* [12]. Since Sherpas and Tibetans split from their common ancestral population ~3200–11,300 years ago, we speculated that Sherpas could also have evolved some distinct adaptations. Taking advantage of the whole-genome sequence data, we identified 68 non-synonymous genetic variants showing high derived allele frequencies (DAF) in Sherpas but not in Tibetans and Han Chinese (Additional file 1: Table S6). To avoid bias from the relatively small sample size of Sherpa sequences, we further validated allele frequencies (AFs) by target-genotyping with much larger Sherpa (*n* = 78) and Tibetan (*n* = 118) sample sizes (Table 1). The identified variants showed relatively smaller population differentiation in their validated AFs than in their sequence data AFs (Additional file 1: Table S6). However, the AF of each NGS panel site correlated linearly with that in the target-genotyping panel (*P* = 0.02), indicating, though not obviously, that differentiations exist in the candidate sites.

**Fig. 7:**
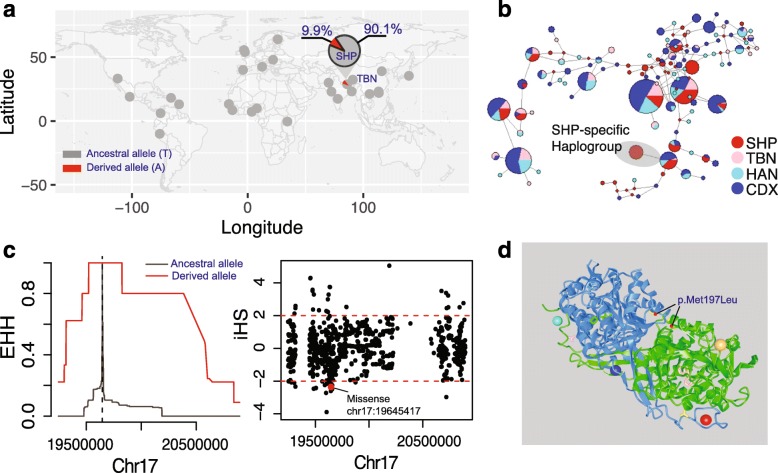
Example of a putatively functional adaptive variant. A novel missense variant (chr17: 19645417) located in *ALDH3A1* was selected as an example. **a** The derived allele frequency (DAF) of this SNP in SHP and TBN was estimated based on the Target-genotyping panel (Tables 1 and 2). **b** Median-joining network of *ALDH3A1* showing a Sherpa-specific haplogroup. Haplotypes consisted of the missense variant and 30 randomly selected shared variants between SHP and non-SHP residing at the *ALDH3A1* region with minor allele frequency (MAF) larger than 5%. The derived allele is specific to SHP in the SHP-specific haplogroup. **c** Positive selection signals of extended haplotype homozygosity (*EHH*) and Integrated Haplotype Score (*iHS*). Analyses in **b** and **c** are based on 55 imputed genomes of Zhangmu Sherpas. **d** Functional consequences of the missense variant

Notably, we pinpointed ten putatively functional missense variants located in genes that could contribute to differential adaptation to extreme high-altitude environments, including hypoxia and high levels of ultraviolet (UV) radiation (Table 2). For instance, *OXR1* (oxidation resistance gene 1 [MIM 605609]) plays pivotal roles in clearing oxidants like reactive oxygen species (ROS), which greatly increase under hypoxic conditions [31, 32], and preventing oxidative stress-induced DNA damage and cell death [33–35]. On the other hand, *ALDH3A1* (aldehyde dehydrogenase 3A1 [MIM 100660]) plays critical and multifaceted roles in protecting the cornea from UV radiation or UV-induced oxidative stress by directly absorbing UV light [36, 37]. Furthermore, *ALDH3A1* expression is nullified by hypoxia [38, 39]. Interestingly, the derived allele of the novel missense variant (chr17: 19645417, GRCh37; Table 2) in *ALDH3A1* was absent in Tibetan (0%) and in other worldwide populations (according to currently accessible databases), but was present in Sherpa (~10%) (Table 2 and Fig. 7a). The homozygosity of the haplotype consisting of the derived allele (A) was extended when measured using extended haplotype homozygosity (EHH) and Integrated Haplotype Score (iHS) (Fig. 7b, c), indicating that positive selection occurred in the *ALDH3A1* region. Results from the population branch statistic (PBS) and cross-population extended haplotype homozygosity (XP-EHH) also supported the selection signal in this genomic region (Additional file 1: Figure S35). This derived allele changes position 197 of the *ALDH3A1* protein sequence (Ensembl protein ID ENSP00000378923) from methionine to leucine (p.Met197Leu) (Fig. 7d), the region of which is highly conserved as measured by CADD and GERP scores (Table 2). Moreover, we identified another novel variant (chr8:108264111, GRCh37) with a derived allele frequency of 7.2 and 0.9% in Sherpas and Tibetans, respectively. This variant is located on *ANGPT1 (*MIM 601667), which is associated with vascular development and angiogenesis [40] and identified as a candidate gene in hypoxia adaptation in Tibetans [8] and grey wolves in the Qinghai-Tibet Plateau [41]. Lastly, *NOS1* (nitric oxide synthases 1 [MIM 163731]) encodes proteins belonging to the family of nitric oxide synthases (*NOS1*, *NOS2* [MIM 163730], and *NOS3* [MIM 163729]) and may regulate oxygen delivery by local paracrine control of vasomotor tone and central control over cardiovascular and respiratory responses [42, 43]. Furthermore, *NOS1* stabilizes HIFα by S-nitrosylation [44]. Additionally, previous studies have reported the *NOS2* locus as a possible selection candidate in the highlanders [45, 46], and a gene–phenotype association study identified that two polymorphisms at the *NOS3* loci are related to nitric oxide (NO) synthesis rates in Nepalese Sherpas [47]. Despite this previous research, our results are the first to indicate that the non-synonymous SNP rs549340789 in *NOS1* might be beneficial for the hypoxic adaptation seen in Sherpas.

**Table 2:** Selected putatively adaptive genetic variants in SHP

Chrom	Position	rsID	Ref	Alt	Ances	DAF_SHPseq_	DAF_TBNseq_	DAF_HANseq_	DAF_TBN*_	DAF_SHP*_	DAF_SHPseq2_	DAF_ESA_	DAF_SAS_	DAF_AFR_	DAF_EUR_	DAF_AMR_	CADD	GERP	Gene	*P* value
8	107691513	rs28921397	A	G	A	0.5	0.0000	0.0000	0.0172	0.0789	0.0000	0.0029	0.0000	0.0000	0.0000	0.0000	**31**	**5.96**	*OXR1*	*0.003*
8	108264111	NeA	G	A	G	0.5	0.0152	0.0000	0.0086	0.0724	0.0000	-	-	-	-	-	**31**	**5.9**	*ANGPT1*	*0.0*
17	19645417	NA	T	A	T	0.4	0.0000	0.0000	0.0000	0.0987	0.0000	-	-	-	-	-	**31**	**4.49**	*ALDH3A1*	*0.0*
3	196921405	rs527829647	A	G	A	0.3	0.0000	0.0000	0.0129	0.0789	0.0000	0.0011	0.0000	0.0000	0.0000	0.0000	**22.3**	**5.17**	*DLG1*	*0.0*
12	117768315	rs549340789	G	A	G	0.3	0.0000	0.0000	0.0086	0.0855	0.0000	0.0022	0.0000	0.0000	0.0000	0.0000	**17.79**	**4.74**	*NOS1*	*0.0*
10	45956828	rs3764990	G	A	G	0.4	0.0303	0.0256	0.0905	0.1974	0.2500	0.0528	0.0226	0.0143	0.0787	0.0293	**18.84**	**5.65**	*MARCH8*	*0.001*
7	21948010	rs200891942	A	G	A	0.3	0.0152	0.0000	0.0129	0.1053	0.0000	0.0010	0.0000	0.0000	0.0000	0.0000	**21.1**	**5.95**	*CDCA7L*	*0.0*
2	37232879	rs2302657	A	C	A	0.3	0.0152	0.0128	0.0086	0.1067	0.2500	0.0325	0.0023	0.0000	0.0000	0.0000	**16.87**	**5.63**	*HEATR5B*	*0.0*
2	109545691	rs61761321	T	C	T	0.3	0.0152	0.1795	0.0345	0.1447	0.0000	0.0910	0.0023	0.0012	0.0000	0.0000	10.27	**4.09**	*EDAR*	*0.0*
1	91405998	rs149597385	C	T	C	0.3	0.0152	0.0000	0.0259	0.1184	0.0000	0.0100	0.0012	0.0000	0.0000	0.0000	**32**	**4.63**	*ZNF644*	*0.001*
1	156551628	rs116035113	G	T	G	0.3	0.0152	0.0000	0.0474	0.1250	0.0000	0.0030	0.0010	0.0475	0.0125	0.0124	**23.9**	**4.83**	*TTC24*	*0.002*
2	46707674	rs116983452	C	T	C	0.4	0.2121	0.0256	0.7241	0.6389	1.0000	0.0207	0.0035	0.0000	0.0000	0.0000	11.18	**2.97**	*TMEM247*	*-*
1	231557623	rs186996510	G	C	G	0.1	0.5910	0.0385	0.5500	0.4800	0.25	0.0100	0.0020	0.0000	0.0000	0.0020	14.73	**3.51**	*EGLN1*	*-*

Among these adaptive genetic variants (AGVs), ten (top 10) showed differences between SHP and TBN, and two (rs116983452 in *TMEM247* near the *EPAS1* region and rs186996510 in *EGLN1*) had similar derived allele frequencies (DAFs), suggesting that both distinct and shared genetic adaptations occurred between TBN and SHP. Conservation scores with CADD >15 and GERP >2 are highlighted in *bold. NA* denotes that the variant is novel and has no current rsID. The physical position of each site follows GRCh37. The *p* value for each candidate was estimated by simulation based on the demographic history of SHP.Zhangmu estimated by MSMC. *Chrom* chromosome, *Ref* reference, *Alt* alteration, *Ances* ancestral, *ESA* East Asians, *SAS* South Asians, *AFR* Africans, *EUR* Europeans, and *AMR* Americans

To rule out the force of drift that could shift allele frequencies, we carry out simulations based on the estimated demographic model. Significant *p* values were obtained for all of the 11 candidate loci showing differentiation between SHP and TBN, indicating that drift alone could not result in the observed AF differences. The variants with elevated, but not extremely high, DAFs, ranging from 8 to 25% in Sherpas (Table 2), could be induced by polygenic adaptation, which would go largely undetected by conventional methods of detecting selection [48]. This does not conflict with high altitude being considered a substantial evolutionary selection pressure [1] since strong positive selection signals were identified in the *EPAS1* region in both Tibetans and Sherpas [1, 6–9, 16]. Moreover, two missense variants, rs116983452 and rs186996510, located in *TMEM247* and in *EGLN1*, respectively (Table 2), both of which are key components (regions) in the HIF pathway for detecting and reacting to changes in oxygen supply [1, 6–9]. The two genes harbor substantially high DAF in both Chinese Sherpas and Tibetans, supporting the premise that they shared adaptive variants. Nonetheless, the identified variants in *ALDH3A1*, *ANGPT1*, and other genes (Table 2) might be the adaptive variants specific to Sherpas. Further efforts to investigate the association of these variants with phenotypic traits, such as blood hemoglobin levels, and to carry out molecular experiments in vitro and in vivo, would provide optimal evidence for validating the adaptive signals.

## Discussion

Despite extensive studies, some questions remain unresolved on the genetic origins, relationships, and adaptive mechanisms of the Sherpa and Tibetan people. A recent study suggested that modern Tibetans are descendants of an admixture of Han Chinese and ancestral Sherpas who began to split from East Asians as early as ~40,000 years ago [16]. However, different conclusions were given by other studies based on mtDNA and Y-chromosome data [17, 18]. Here, we propose that the Sherpas split from Tibetans more recently following the divergence of ancestral populations of Tibetans and Han Chinese. The controversy could result from different interpretations of the ancestry patterns observed in Sherpas and Tibetans, although the *ADMIXTURE* results (*K* = 4 and *K* = 5) are similar between our study and Jeong et al. (Fig. 1 in Jeong et al. and Additional file 1: Figures S17 and S18 in our analysis). According to Jeong et al., unsupervised *ADMIXTURE* infers Tibetans as a mixture of two genetic components: one is highly enriched in the Sherpa population (but rare in lowland populations), which was referred to as the “high-altitude component”, and the other is enriched in low-altitude East Asians, which was referred to as the “low-altitude component”. However, it is challenging to determine whether clustering patterns among groups resulted from recent admixture between distinct ancestral populations or shared ancestry prior to the population divergence [49, 50]. On the contrary, we suggest the high-altitude component shared between the Tibetans and Sherpas was more likely from the shared ancestry prior to their divergence. Moreover, a much larger number of full sequence data obtained from our study, including 33 Tibetan, 5 Sherpa, and 38 Han Chinese genomes which were not available to previous studies, enabled us to make a more sophisticated estimation of evolutionary genetic parameters such as divergence time. By using MSMC analysis, we show that Sherpas (both Chinese Sherpas and Nepalese Sherpas) split from Tibetans much more recently (~7000 years ago), following the divergence event between Tibetans and the Han Chinese (~9000 years ago) (Fig. 5; Additional file 1: Figure S34). The previous study estimated that Sherpas began to diverge from the Han Chinese and Dai ~40,000 years [16], which could be biased due to limited sequence data (only two sequences were available) and the analysis relying on PSMC being based on single genomes. Additional uncertainties could have resulted from a long history of isolation which Nepalese Sherpas have experienced (Additional file 1: Figure S21).

The role of geography and culture in migration and population structure is a central topic in human evolutionary genetics. We show that population substructures exist within Ü-Tsang, Kham, and Amdo Tibetans, possibly attributing to the differentiation of culture in historical Tibet and the natural barriers from complex terrain surrounding high transverse valleys in the Qinghai-Tibet Plateau, which hindered communication between subgroups. Our observations of substantial East Asian genetic influence on Tibetans and the presence of gene flow from Tibetans to Sherpas support the direction of gene flow from East Asia into Tibet and Nepal [51–54]. Although the natural barrier of the Himalayas (low-oxygen environment [55]) effectively limited gene flow from South Asia, the observed genetic component (Fig. 3) and admixture signals from South Himalayan populations in Sherpas (Additional file 1: Figure S26) indicated bi-directional gene flow. Furthermore, gene flow from South Asians was selectively permeable and highlanders who adapted well to high altitudes could relatively easily pass the Himalayas and dwell in highlander regions. Sherpas, therefore, were the genetic carriers who transferred the South Asian ancestry from south of the Himalayas to the north. In our estimation, Sherpas show 3.5% (0.3 ± 1.2% for SHP.Khumbu and 6.2 ± 5.0% for SHP.Zhangmu) South Asian ancestry, with proportions ranging from 0 to 20% across individuals. This estimation is consistent with results from mtDNA investigations [18, 56], in which proportions were reported to be between 0.34 and 2.53% in Nepalese Sherpa and 8 and 17% in Chinese Sherpa.

In this study, we reveal complex population structures of Tibetans and Sherpas which further indicate the complicated history of two groups. However, since the current study enrolled only two Sherpa subgroups, Zhangmu Sherpa and Khumbu Sherpa, with a limited sample size (Fig. 1 and Table 1), we believe that the complete landscape of genetic diversity in Sherpas requires further investigation by increasing the number of Sherpa subgroups and increasing sample sizes.

## Conclusions

Sherpas and Tibetans show sufficient genetic difference and can be distinguished as two distinct groups; on the other hand, their divergence time (~3200–11,300 years ago) is much more recent than that of their common ancestors and Han Chinese (~6200–16,000 years). The two highlander groups harbor shared and differentiated genetic variants associated with adaptation to either high-altitude or UV radiation. Our analysis indicates that Tibetan highlanders share a common genetic origin but experienced a complex history of population divergence, a long period of isolation, local adaptation, and recent gene flow, which jointly shaped the genetic landscape of human genetic diversity on the plateau.

## Methods

### Sample acquisition

Peripheral blood samples were collected from 78 high-altitude native Sherpas (SHP) living in Zhangmu, Nyalam County, and 118 Tibetans (TBN) residing at >3000 m in six prefectures (Lhasa, Nyingchi, Chamdo, Shannan, Shigatse, and Nagqu) of the Tibet Autonomous Region (Fig. 1 and Table 1). For comparison, 39 Han Chinese (HAN) individuals residing at low altitude were also collected. Each individual was the offspring of a non-consanguineous marriage of members of the same nationality within three generations. All samples were collected with informed consent and approved by the Biomedical Research Ethics Committee of the Shanghai Institutes for Biological Sciences (number ER-SIBS-261408).

### Genotyping and whole-genome sequencing

For diverse genetic analysis, the collected samples were subjected to genotyping, whole-genome sequencing, and SNP target-genotyping. In total, 61 Sherpa and 66 Tibetan samples were genotyped using Affymetrix Genome-wide Human SNP Array 6.0, which contains more than 906,600 single nucleotide polymorphism (SNP) loci. We removed samples with identity by descent (IBD) larger than 35% or missing rate larger than 10%. SNPs with a low call rate (<90%) were also filtered based on analysis with *PLINK* v1.07 [57]. Moreover, five Sherpas, 33 Tibetans, and 39 HAN Chinese individuals were chosen for whole-genome sequencing, with 30× coverage for 150-bp paired-end reads, using Illumina HiSeq X performed in Wuxi NextCODE at Shanghai, China (Table 1), detailed methods of which were described in our recent work [12, 58]. For quality control, three Sherpa and 18 Tibetan samples were replicated by both genotyping and whole-genome sequencing as mentioned above.

To analyze the genetic variation of Sherpas and Tibetans in a broader context, we obtained data for 81 genotyped Tibetans from two previous studies [7, 20]. We also included data from 49 genotyped and two sequenced Sherpas reported by Jeong et al. [16] and 2345 individuals genotyped on the Affymetrix Axiom Genome-wide Human Origins 1 Array, described by Patterson et al. [22] (Fig. 1; Table 1; Additional file 1: Figure S1). All Sherpa and Tibetan individuals were classified into different subgroups (SHP.Zhangmu, SHP.Khumbu, TBN.Shigatse, TBN.Shannan, TBN.Lhasa, TBN.Nyingchi, TBN.Chamdo, and TBN.Qinghai) according to geographic location. Given the differences between platforms used in each dataset and the various genetic analyses that would be performed, we divided the combined datasets into three panels as described below (Table 1).

#### Panel 1

Panel 1 included all genotyped individuals generated by Affymetrix technology and two sequenced Nepalese Sherpas. This data set contained 156,143 overlapping SNPs after removing SNPs with call rates <90% or with strand-ambiguity.

#### Panel 2

Panel 2 combined all genotyped individuals generated by Affymetrix technology with the 49 unrelated Nepalese Sherpas genotyped by Illumina technology reported by Jeong et al. [16]. The number of overlapping SNPs (81,023) in panel 2 was much lower than that in panel 1 (156,143), which was due to the different platforms used. However, panel 2 enrolled a larger number of Nepalese Sherpas, making the two Sherpa subgroups (Chinese Sherpas and Nepalese Sherpas) more comparable, despite panel 1 harboring many more SNPs and being less affected by batch effects induced by different platforms (Affymetrix and Illumina). To obtain more reliable results, we performed analysis using both panels. Panel 2 was used as the default panel when not specified.

#### NGS panel

Given the insufficient genetic information provided by the chip genotyped panels (panels 1 and 2), we included all sequence data generated in this study to conduct more comprehensive analyses, including estimating effective population size (*N*_e_), population divergence time, and the time to the most recent common ancestor (TMRCA), as well as other analyses when needed. In addition, seven sequenced Indians (IDN) were also included [24] to represent South Asian population to enable more comprehensive analysis.

#### SNP target-genotyping panel

In total, 68 SNPs (Table 1; Additional file 1: Table S5) were hierarchically genotyped for 78 Sherpa and 118 Tibetan samples with a SNaPshot Multiplex Kit (Applied Biosystems, Foster City, CA, USA) and fluorescent allele-specific PCR. Products (fragments) were then read on a 3730xl Genetic Analyzer (Applied Biosystems). A series of primers designed for covering these genetic regions are listed in Additional file 1: Table S1.

### Estimation of *F*_ST_, outgroup *f*_3_ statistics, and AMOVA

*F*_ST_ between each population pair was measured following Weir and Cockerham [59]. To reduce the influence of large sample size differences between populations, populations with sample sizes less than 5 were not included for pairwise comparison. First, Sherpa (SHP) and Tibetan (TBN) samples were taken as single groups, and *F*_ST_ was calculated between each group, and also between each group and other worldwide populations. Next, the *F*_ST_ between each SHP or TBN subgroup and other populations was estimated in both genotyped panels (panels 1 and 2).

When performing outgroup *f*_*3*_ statistics [60], we assumed no admixture had occurred in a tree with topology (YRI; A, B), where the expected value was proportional to the shared genetic history between A and B. That is, the larger the *f*_*3*_ value, the greater the genetic relatedness between the two populations. *ADMIXTOOLS* [22] with the *qp3pop* program was employed to calculate outgroup *f*_3_ statistics in the form of *f*_3_(SHP; Yoruba, X) or *f*_3_(TBN; Yoruba, X), where X represents East Asian, Central Asian/Siberian, or South Asian populations. Similar analyses were also carried out when comparing the genetic relatedness between SHP or TBN subgroups and their surrounding populations.

We used *Arlequin* v3.5 [61] to perform AMOVA. We estimated the genetic variance among the two highlander groups (SHP and TBN) and among sub-populations within the two groups using Arlequin. We further performed random sorting by separating SHP.Zhangmu + SHP.Khumbu + X as one group, and the rest of the populations as the other group, where X represents one of the Tibetan sub-populations (TBN.Shigatse, TBN.Shannan, TBN.Lhasa, TBN.Nyingchi, TBN.Chamdo, and TBN.Qinghai). For each X, we repeated the estimation of variance among groups or among sub-populations within a group. We compared the among-group variance with that among sub-populations within groups. Given Tibetans and Sherpas are genetically different ethnic groups, the variance between SHP and TBN groups is expected to be larger than that within the groups, while the variance within SHP.Zhangmu + SHP.Khumbu + X and non-X groups is expected to exceed that between groups.

### PCA, admixture analysis with *ADMIXTURE, f* statistics, and *TreeMix*

To investigate fine-scale population structures, we performed a series of PCAs using *EIGENSOFT* v3.0 [62] by gradually removing outliers based on the first and second principal components (PCs) and reanalyzing the remaining samples based on the same set of SNP markers.

For unsupervised clustering analysis, we used *ADMIXTURE* v1.30 [63] with cross-validation (CV) to find the optimal number of clusters. Since the model in *ADMIXTURE* does not take linkage disequilibrium (LD) into consideration, we generated an LD-pruned dataset using an *r* ^2^ cutoff of 0.1 in each continuous window of 50 SNPs, and advanced by 10 SNPs (−−indep-pairwise 50 10 0.1) using *PLINK* v1.07 [57]. We ran *ADMIXTURE* with random seeds for the dataset from *K* = 2 to *K* = 20 with default parameters (−−cv = 5) in ten replicates for each *K*. We assessed the CV error in the ten replicates to find the best *K* of the ancestral populations. The *K*s that best explained our data and best represented the population structure of highlanders were 4, 5, and 6.

To detect gene flow between populations, we used *f*_3_ statistics by assuming one population from SHP, TBN, or their subgroups to be a potential admixed population, another highlander population, and a third from surrounding populations as ancestral populations. Tests were performed with *qp3pop* in *ADMIXTOOLS* [22]. We used *qpDstat* (*f*_4_ statistics) to estimate the relative contribution from ancestral populations to SHP and TBN. We also ran *TreeMix* [23] to infer the ancestral populations contributing to the TBN and SHP gene pools.

### Estimation of historical population effective sizes (*N*_e_) and divergence time

We applied multiple sequentially Markovian coalescent (MSMC) analysis [25] to infer the *N*_e_ of SHP, TBN, HAN, and IDN from high-coverage genomes in the NGS panel. The whole-genome sequences were phased by *SHAPEIT2* with the 1000 Genomes phase 1 data as a reference panel [64]. *N*_e_ estimations were based on autosomal sequences by analyzing two genomes (four haplotypes), four genomes (eight haplotypes), and five genomes (ten haplotypes) for each population separately, using the following options: −N 25 -t 15 -r 5 -p "4 + 25*2 + 4 + 6". Since only two Nepalese Sherpas were included in the NGS panel, we therefore chose results based on four haplotypes for our main estimate. The time of divergence was also estimated by MSMC, and involved similar strategies as those implemented in *N*_e_ estimation. To convert population parameter estimates into *N*_e_ and time in years, we used an autosomal neutral mutation rate of *μ*_Auto_ = 1.25 × 10^−8^ per base-pair per generation and 25 years per generation [65]. Besides MSMC, we estimated *N*_e_ for TBN, SHP, and their subgroups based on LD decay by the following formula: *N*_*e*_ = 1/(4*c*) × [(1/*r*_LD_^2^) − 2] for *t* generations ago with chip array data (panels 1 and 2), where *c* is the recombination distance between loci in Morgans (M) and *t* = 1/(2*c*) [30]. As experimental sampling introduces chance LD, all individual *r*_LD_^2^ values were adjusted as *r*_LD_^2^ – (1/*n*), where *n* is the sample size prior to the calculation of *N*_e_. We calculated *N*_e_ for each subgroup with recombination distances ranging from 0.01 to 0.25 centimorgan (cM), corresponding to 125 to 5000 years ago, with 25 years per generation. The divergence times measured by *T*_*F*_ in generations based on the chip array dataset (panel 2) were also estimated [30]. Here *T*_*F*_ = 2*N*_*e*_ *F*_ST_, where *N*_*e*_ is the harmonic mean *N*_*e*_ of two target populations. Divergence times between SHP, TBN, and HAN were also estimated using G-PhoCS [29] with one individual genome randomly sampled from each population. Variants were filtered using “data quality filters” and “comparative filters” as suggested by the authors of G-PhoCS. Only regions with a length of 1000 bp were retained for further analysis. In total 35,279 regions were finally used for G-PhoCS analysis. G-PhoCS was run with a burn-in of 100,000 iterations followed by 400,000 sampling iterations.

### Analysis of natural selection, median-joining network, functional annotation, and simulation

To estimate the positive selection signal of a genomic region, we calculated extended haplotype homozygosity (EHH) [66] and Integrated Haplotype Score (iHS) [67] with the *R* package *REHH* [68]. Cross-population extended haplotype homozygosity (XP-EHH) was calculated using selscan [69]. Population branch statistic (PBS) was calculated using an in-house perl script. The genomes used were from 55 Zhangmu Sherpas imputed from the microarray data set with 259 sequenced genomes (>30×, unpublished but including five Sherpas in Table 1) as references by *BEAGLE* v4.0 [70]. A median-joining haplotype network was constructed following methods discussed by Bandelt et al. [71]. The haplotypes consisted of the novel missense variant (chr17: 19645417, GRCh37) in *ALDH3A1* and 30 randomly selected variants shared by SHP and non-SHP (including TBN and HAN in this study, and CDX in the 1000 Genomes Project [72]) residing at the gene region with minor allele frequency larger than 5%. Functional annotation, such as variant type, gene mapping, CADD [73], and GERP++ [74] scores, was performed using the variant effect predictor (VEP) [75]. Lastly, the protein structure of *ALDH3A1* was obtained from the NCBI Structure database. To rule out the force of drift that shifted allele frequencies, we carried out simulation based on the demographic history of Chinese Sherpas inferred by MSMC analysis (as illustrated in Fig. 5). For each candidate locus, we used the allele frequency of present TBN as the initial frequency of the ancient SHP who split from TBN ~ 7000 years ago (~280 generations ago). We next estimated the allele frequency of present SHP if the ancient SHP experienced 280 generations of drift assuming the *N*_*e*_ was 11,000. We simulated the process of drift 1000 times for each candidate and compared the observed allele frequency of the given site with the distribution of simulated frequencies.

## Additional file

Additional file 1: Figures S1–S35. and **Table S1–S6.** (PDF 35929 kb)

## References

[1] Beall CM, Cavalleri GL, Deng L, Elston RC, Gao Y, Knight J, Li C, Li JC, Liang Y, McCormack M (2010). Natural selection on EPAS1 (HIF2alpha) associated with low hemoglobin concentration in Tibetan highlanders. Proc Natl Acad Sci U S A.

[2] Samaja M, Veicsteinas A, Cerretelli P (1979). Oxygen affinity of blood in altitude Sherpas. J Appl Physiol Respir Environ Exerc Physiol.

[3] Hackett PH, Reeves JT, Reeves CD, Grover RF, Rennie D (1980). Control of breathing in Sherpas at low and high altitude. J Appl Physiol Respir Environ Exerc Physiol.

[4] Holden JE, Stone CK, Clark CM, Brown WD, Nickles RJ, Stanley C, Hochachka PW (1985). Enhanced cardiac metabolism of plasma glucose in high-altitude natives: adaptation against chronic hypoxia. J Appl Physiol.

[5] Hochachka PW, Clark CM, Monge C, Stanley C, Brown WD, Stone CK, Nickles RJ, Holden JE (1996). Sherpa brain glucose metabolism and defense adaptations against chronic hypoxia. J Appl Physiol.

[6] Yi X, Liang Y, Huerta-Sanchez E, Jin X, Cuo ZX, Pool JE, Xu X, Jiang H, Vinckenbosch N, Korneliussen TS (2010). Sequencing of 50 human exomes reveals adaptation to high altitude. Science.

[7] Peng Y, Yang Z, Zhang H, Cui C, Qi X, Luo X, Tao X, Wu T, Ouzhuluobu, Basang (2011). Genetic variations in Tibetan populations and high-altitude adaptation at the Himalayas. Mol Biol Evol.

[8] Wang B, Zhang YB, Zhang F, Lin H, Wang X, Wan N, Ye Z, Weng H, Zhang L, Li X (2011). On the origin of Tibetans and their genetic basis in adapting high-altitude environments. PLoS One.

[9] Xu S, Li S, Yang Y, Tan J, Lou H, Jin W, Yang L, Pan X, Wang J, Shen Y (2011). A genome-wide search for signals of high-altitude adaptation in Tibetans. Mol Biol Evol.

[10] Aldenderfer M (2011). Peopling the Tibetan plateau: insights from archaeology. High Alt Med Biol.

[11] Qi X, Cui C, Peng Y, Zhang X, Yang Z, Zhong H, Zhang H, Xiang K, Cao X, Wang Y (2013). Genetic evidence of paleolithic colonization and neolithic expansion of modern humans on the tibetan plateau. Mol Biol Evol.

[12] Lu D, Lou H, Yuan K, Wang X, Wang Y, Zhang C, Lu Y, Yang X, Deng L, Zhou Y (2016). Ancestral origins and genetic history of Tibetan highlanders. Am J Hum Genet.

[13] Oppitz M (1968). Myths and facts: reconsidering some data concerning the clan history of the Sherpas. Kailash.

[14] Stevens SF (1993). Claiming the high ground: Sherpas, subsistence, and environmental change in the highest himalaya. Geogr Rev.

[15] Norbu L (2008). Through a Sherpa window : illustrated guide to traditional Sherpa culture.

[16] Jeong C, Alkorta-Aranburu G, Basnyat B, Neupane M, Witonsky DB, Pritchard JK, Beall CM, Di Rienzo A (2014). Admixture facilitates genetic adaptations to high altitude in Tibet. Nat Commun.

[17] Kang L, Zheng HX, Chen F, Yan S, Liu K, Qin Z, Liu L, Zhao Z, Li L, Wang X (2013). mtDNA lineage expansions in Sherpa population suggest adaptive evolution in Tibetan highlands. Mol Biol Evol.

[18] Bhandari S, Zhang X, Cui C, Bianba, Liao S, Peng Y, Zhang H, Xiang K, Shi H, Ouzhuluobu, et al. Genetic evidence of a recent Tibetan ancestry to Sherpas in the Himalayan region. Sci Rep. 2015;5:16249 10.1038/srep16249 PMC4633682 26538459

[19] Gayden T, Bukhari A, Chennakrishnaiah S, Stojkovic O, Herrera RJ (2012). Y-chromosomal microsatellite diversity in three culturally defined regions of historical Tibet. Forensic Sci Int Genet.

[20] Simonson TS, Yang Y, Huff CD, Yun H, Qin G, Witherspoon DJ, Bai Z, Lorenzo FR, Xing J, Jorde LB (2010). Genetic evidence for high-altitude adaptation in Tibet. Science.

[21] Raghavan M, Skoglund P, Graf KE, Metspalu M, Albrechtsen A, Moltke I, Rasmussen S, Stafford TW, Orlando L, Metspalu E (2014). Upper Palaeolithic Siberian genome reveals dual ancestry of Native Americans. Nature.

[22] Patterson N, Moorjani P, Luo Y, Mallick S, Rohland N, Zhan Y, Genschoreck T, Webster T, Reich D (2012). Ancient admixture in human history. Genetics.

[23] Pickrell JK, Pritchard JK (2012). Inference of population splits and mixtures from genome-wide allele frequency data. PLoS Genet.

[24] Chambers JC, Abbott J, Zhang W, Turro E, Scott WR, Tan ST, Afzal U, Afaq S, Loh M, Lehne B (2014). The South Asian genome. PLoS One.

[25] Schiffels S, Durbin R (2014). Inferring human population size and separation history from multiple genome sequences. Nat Genet.

[26] Barton L, Newsome SD, Chen FH, Wang H, Guilderson TP, Bettinger RL (2009). Agricultural origins and the isotopic identity of domestication in northern China. Proc Natl Acad Sci U S A.

[27] Bettinger RL, Barton L, Morgan C (2010). The origins of food production in north China: A different kind of agricultural revolution. Evol Anthropol.

[28] Yang X, Wan Z, Perry L, Lu H, Wang Q, Zhao C, Li J, Xie F, Yu J, Cui T (2012). Early millet use in northern China. Proc Natl Acad Sci U S A.

[29] Gronau I, Hubisz MJ, Gulko B, Danko CG, Siepel A (2011). Bayesian inference of ancient human demography from individual genome sequences. Nat Genet.

[30] McEvoy BP, Powell JE, Goddard ME, Visscher PM (2011). Human population dispersal "Out of Africa" estimated from linkage disequilibrium and allele frequencies of SNPs. Genome Res.

[31] Clanton TL (2007). Hypoxia-induced reactive oxygen species formation in skeletal muscle. J Appl Physiol.

[32] Chandel NS, McClintock DS, Feliciano CE, Wood TM, Melendez JA, Rodriguez AM, Schumacker PT (2000). Reactive oxygen species generated at mitochondrial complex III stabilize hypoxia-inducible factor-1alpha during hypoxia: a mechanism of O2 sensing. J Biol Chem.

[33] Oliver PL, Finelli MJ, Edwards B, Bitoun E, Butts DL, Becker EBE, Cheeseman MT, Davies B, Davies KE (2011). Oxr1 is essential for protection against oxidative stress-induced neurodegeneration. PLoS Genet.

[34] Elliott NA, Volkert MR (2004). Stress induction and mitochondrial localization of Oxr1 proteins in yeast and humans. Mol Cell Biol.

[35] Volkert MR, Elliott NA, Housman DE (2000). Functional genomics reveals a family of eukaryotic oxidation protection genes. Proc Natl Acad Sci U S A.

[36] Pappa A, Chen C, Koutalos Y, Townsend AJ, Vasiliou V (2003). Aldh3a1 protects human corneal epithelial cells from ultraviolet- and 4-hydroxy-2-nonenal-induced oxidative damage. Free Radical Biol Med.

[37] Estey T, Cantore M, Weston PA, Carpenter JF, Petrash JM, Vasiliou V (2007). Mechanisms involved in the protection of UV-induced protein inactivation by the corneal crystallin ALDH3A1. J Biol Chem.

[38] Manzer R, Pappa A, Estey T, Sladek N, Carpenter JF, Vasiliou V (2003). Ultraviolet radiation decreases expression and induces aggregation of corneal ALDH3A1. Chem Biol Interact.

[39] Reisdorph R, Lindahl R (2007). Constitutive and 3-methylcholanthrene-induced rat ALDH3A1 expression is mediated by multiple xenobiotic response elements. Drug Metab Dispos.

[40] Jeansson M, Gawlik A, Anderson G, Li C, Kerjaschki D, Henkelman M, Quaggin SE (2011). Angiopoietin-1 is essential in mouse vasculature during development and in response to injury. J Clin Invest.

[41] Zhang W, Fan Z, Han E, Hou R, Zhang L, Galaverni M, Huang J, Liu H, Silva P, Li P (2014). Hypoxia adaptations in the grey wolf (Canis lupus chanco) from Qinghai-Tibet Plateau. PLoS Genet.

[42] Galkin A, Higgs A, Moncada S (2007). Nitric oxide and hypoxia. Essays Biochem.

[43] Umbrello M, Dyson A, Feelisch M, Singer M (2013). The key role of nitric oxide in hypoxia: hypoxic vasodilation and energy supply–demand matching. Antioxid Redox Signal.

[44] Li F, Sonveaux P, Rabbani ZN, Liu S, Yan B, Huang Q, Vujaskovic Z, Dewhirst MW, Li CY (2007). Regulation of HIF-1alpha stability through S-nitrosylation. Mol Cell.

[45] Bigham AW, Mao X, Mei R, Brutsaert T, Wilson MJ, Julian CG, Parra EJ, Akey JM, Moore LG, Shriver MD. Identifying positive selection candidate loci for high-altitude adaptation in Andean populations. Hum Genomics. 2009. 10.1186/1479-7364-4-2-79 PMC2857381 20038496

[46] Bigham A, Bauchet M, Pinto D, Mao X, Akey JM, Mei R, Scherer SW, Julian CG, Wilson MJ, Lopez Herraez D, Brutsaert T, et al. Identifying signatures of natural selection in Tibetan and Andean populations using dense genome scan data. PLoS Genet. 2010. 10.1371/journal.pgen.1001116 PMC2936536 20838600

[47] Droma Y, Hanaoka M, Basnyat B, Arjyal A, Neupane P, Pandit A, Sharma D, Miwa N, Ito M, Katsuyama Y, Ota M, et al. Genetic contribution of the endothelial nitric oxide synthase gene to high altitude adaptation in sherpas. High Alt Med Biol. 2006. 10.1089/ham.2006.7.209 16978133

[48] Pritchard JK, Di Rienzo A (2010). Adaptation--not by sweeps alone. Nat Rev Genet.

[49] van Dorp L, Balding D, Myers S, Pagani L, Tyler-Smith C, Bekele E, Tarekegn A, Thomas MG, Bradman N, Hellenthal G (2015). Evidence for a common origin of blacksmiths and cultivators in the Ethiopian Ari within the last 4500 years: lessons for clustering-based inference. PLoS Genet.

[50] Li JZ, Absher DM, Tang H, Southwick AM, Casto AM, Ramachandran S, Cann HM, Barsh GS, Feldman M, Cavalli-Sforza LL, Myers RM (2008). Worldwide human relationships inferred from genome-wide patterns of variation. Science.

[51] Gayden T, Cadenas AM, Regueiro M, Singh NB, Zhivotovsky LA, Underhill PA, Cavalli-Sforza LL, Herrera RJ (2007). The Himalayas as a directional barrier to gene flow. Am J Hum Genet.

[52] Gayden T, Mirabal S, Cadenas AM, Lacau H, Simms TM, Morlote D, Chennakrishnaiah S, Herrera RJ (2009). Genetic insights into the origins of Tibeto-Burman populations in the Himalayas. J Hum Genet.

[53] Wang HW, Li YC, Sun F, Zhao M, Mitra B, Chaudhuri TK, Regmi P, Wu SF, Kong QP, Zhang YP (2012). Revisiting the role of the Himalayas in peopling Nepal: insights from mitochondrial genomes. J Hum Genet.

[54] Jeong C, Ozga AT, Witonsky DB, Malmstrom H, Edlund H, Hofman CA, Hagan RW, Jakobsson M, Lewis CM, Aldenderfer MS, et al. Long-term genetic stability and a high-altitude East Asian origin for the peoples of the high valleys of the Himalayan arc. Proc Natl Acad Sci U S A. 2016. 10.1073/pnas.1520844113 PMC4941446 27325755

[55] Cordaux R, Weiss G, Saha N, Stoneking M (2004). The northeast Indian passageway: a barrier or corridor for human migrations?. Mol Biol Evol.

[56] Kang L, Wang CC, Chen F, Yao D, Jin L, Li H (2016). Northward genetic penetration across the Himalayas viewed from Sherpa people. Mitochondrial DNA.

[57] Purcell S, Neale B, Todd-Brown K, Thomas L, Ferreira MA, Bender D, Maller J, Sklar P, de Bakker PI, Daly MJ, Sham PC (2007). PLINK: a tool set for whole-genome association and population-based linkage analyses. Am J Hum Genet.

[58] Mallick S, Li H, Lipson M, Mathieson I, Gymrek M, Racimo F, Zhao M, Chennagiri N, Nordenfelt S, Tandon A, et al. The Simons Genome Diversity Project: 300 genomes from 142 diverse populations. Nature. 2016; advance online publication. 10.1038/nature18964 PMC5161557 27654912

[59] Weir BS, Cockerham CC (1984). Estimating F-statistics for the analysis of population structure. Evolution.

[60] Reich D, Thangaraj K, Patterson N, Price AL, Singh L (2009). Reconstructing Indian population history. Nature.

[61] Excoffier L, Lischer HE (2010). Arlequin suite ver 3.5: a new series of programs to perform population genetics analyses under Linux and Windows. Mol Ecol Resour.

[62] Patterson N, Price AL, Reich D (2006). Population structure and eigenanalysis. PLoS Genet.

[63] Alexander DH, Novembre J, Lange K (2009). Fast model-based estimation of ancestry in unrelated individuals. Genome Res.

[64] O'Connell J, Gurdasani D, Delaneau O, Pirastu N, Ulivi S, Cocca M, Traglia M, Huang J, Huffman JE, Rudan I (2014). A general approach for haplotype phasing across the full spectrum of relatedness. PLoS Genet.

[65] Scally A, Durbin R (2012). Revising the human mutation rate: implications for understanding human evolution. Nat Rev Genet.

[66] Sabeti PC, Reich DE, Higgins JM, Levine HZ, Richter DJ, Schaffner SF, Gabriel SB, Platko JV, Patterson NJ, McDonald GJ (2002). Detecting recent positive selection in the human genome from haplotype structure. Nature.

[67] Voight BF, Kudaravalli S, Wen X, Pritchard JK (2006). A map of recent positive selection in the human genome. PLoS Biol.

[68] Gautier M, Vitalis R (2012). rehh: an R package to detect footprints of selection in genome-wide SNP data from haplotype structure. Bioinformatics.

[69] Szpiech ZA, Hernandez RD (2014). selscan: an efficient multithreaded program to perform EHH-based scans for positive selection. Mol Biol Evol.

[70] Browning BL, Browning SR (2011). A fast, powerful method for detecting identity by descent. Am J Hum Genet.

[71] Bandelt HJ, Forster P, Rohl A (1999). Median-joining networks for inferring intraspecific phylogenies. Mol Biol Evol.

[72] Genomes Project C, Auton A, Brooks LD, Durbin RM, Garrison EP, Kang HM, Korbel JO, Marchini JL, McCarthy S, McVean GA, Abecasis GR (2015). A global reference for human genetic variation. Nature.

[73] Kircher M, Witten DM, Jain P, O'Roak BJ, Cooper GM, Shendure J (2014). A general framework for estimating the relative pathogenicity of human genetic variants. Nat Genet.

[74] Davydov EV, Goode DL, Sirota M, Cooper GM, Sidow A, Batzoglou S (2010). Identifying a high fraction of the human genome to be under selective constraint using GERP++. PLoS Comput Biol.

[75] McLaren W, Pritchard B, Rios D, Chen Y, Flicek P, Cunningham F (2010). Deriving the consequences of genomic variants with the Ensembl API and SNP Effect Predictor. Bioinformatics.

