# Supplementary material for: Differentiated demographic histories and local adaptations between Sherpas and Tibetans
Source: Genome Biol. 2017 Jun 15;18:115. doi: 10.1186/s13059-017-1242-y (PMC5472941; doi:10.1186/s13059-017-1242-y)
Supplement: Additional file 1: Figures S1–S35. — and Table S1–S6. (PDF 35929 kb) [file 13059_2017_1242_MOESM1_ESM.pdf]

Additional file 1

Figure S1 Geographical locations of worldwide samples analyzed in this study. We combine all TBN and SHP array data with 203 worldwide populations obtained from human origins genotyping dataset. Each red dot represents a particular population located in the corresponding geographic region in the world map. See Table S1 for further information.

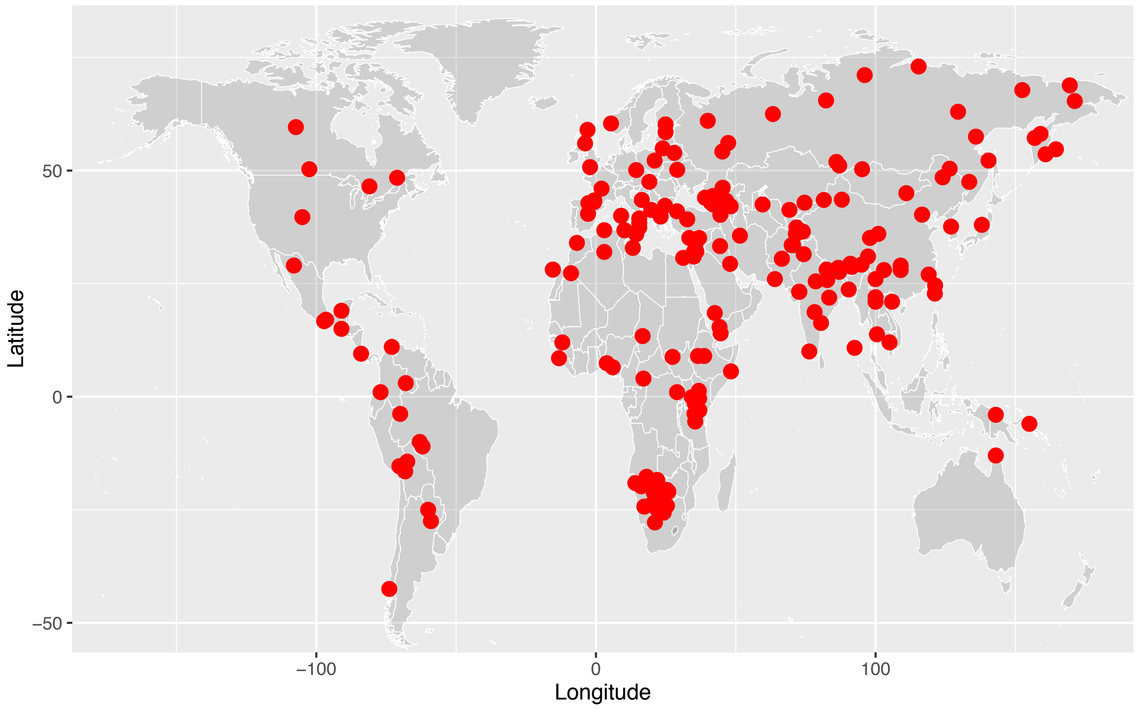

Figure S2 Overview of genetic affinities of (A, C) SHP and (B, D) TBN measured by F_{ST} in the context of worldwide populations. The analysis was based on panel 2 since more SHP.Khumbu individuals were included in it.

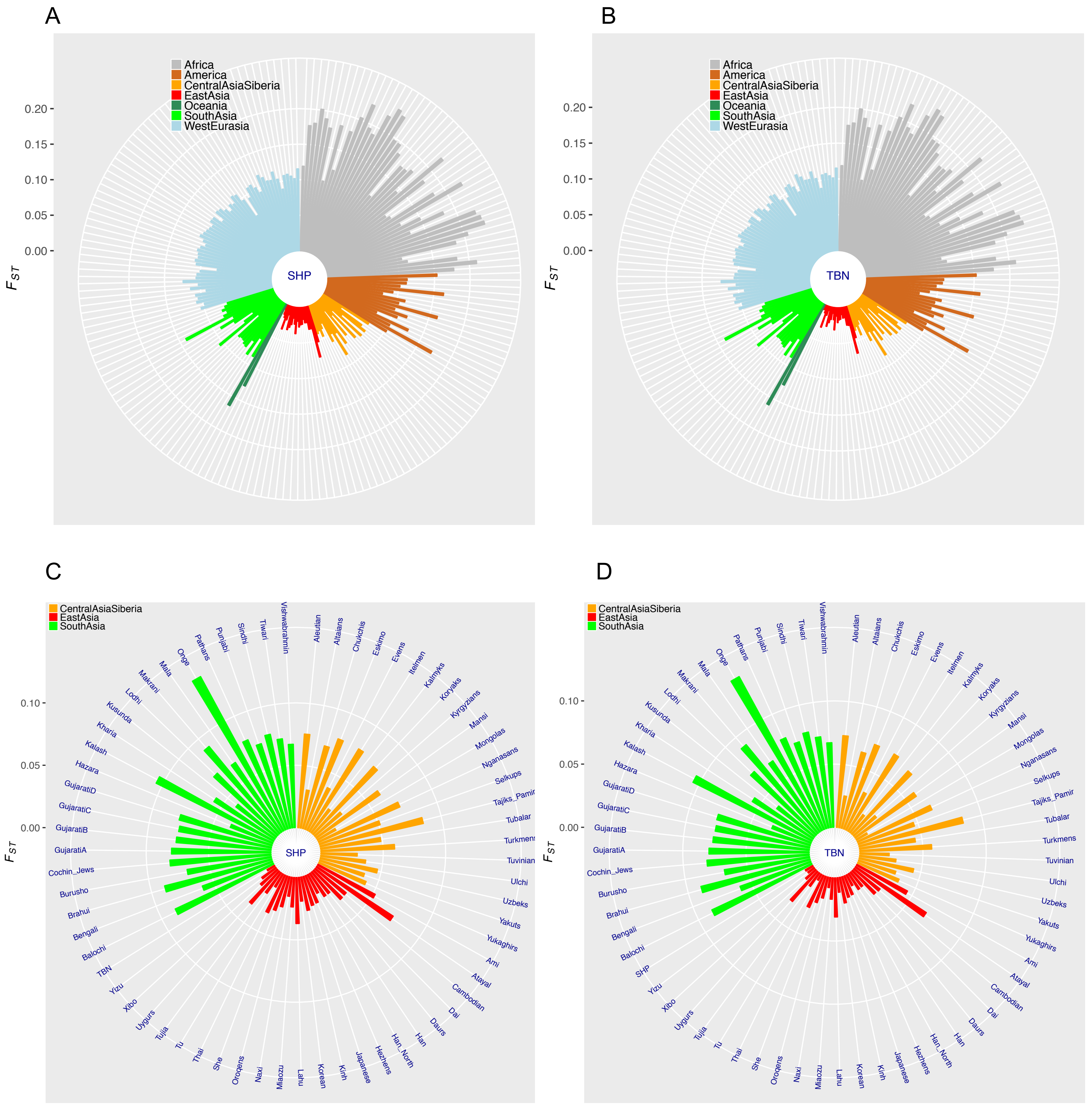

Figure S3 ***F*$_{ST}$ between SHP (or TBN) and populations from East Asians, Central Asian/Siberians and South Asians.** The analysis was based on panel 2 since more SHP.Khumbu individuals were included in it.

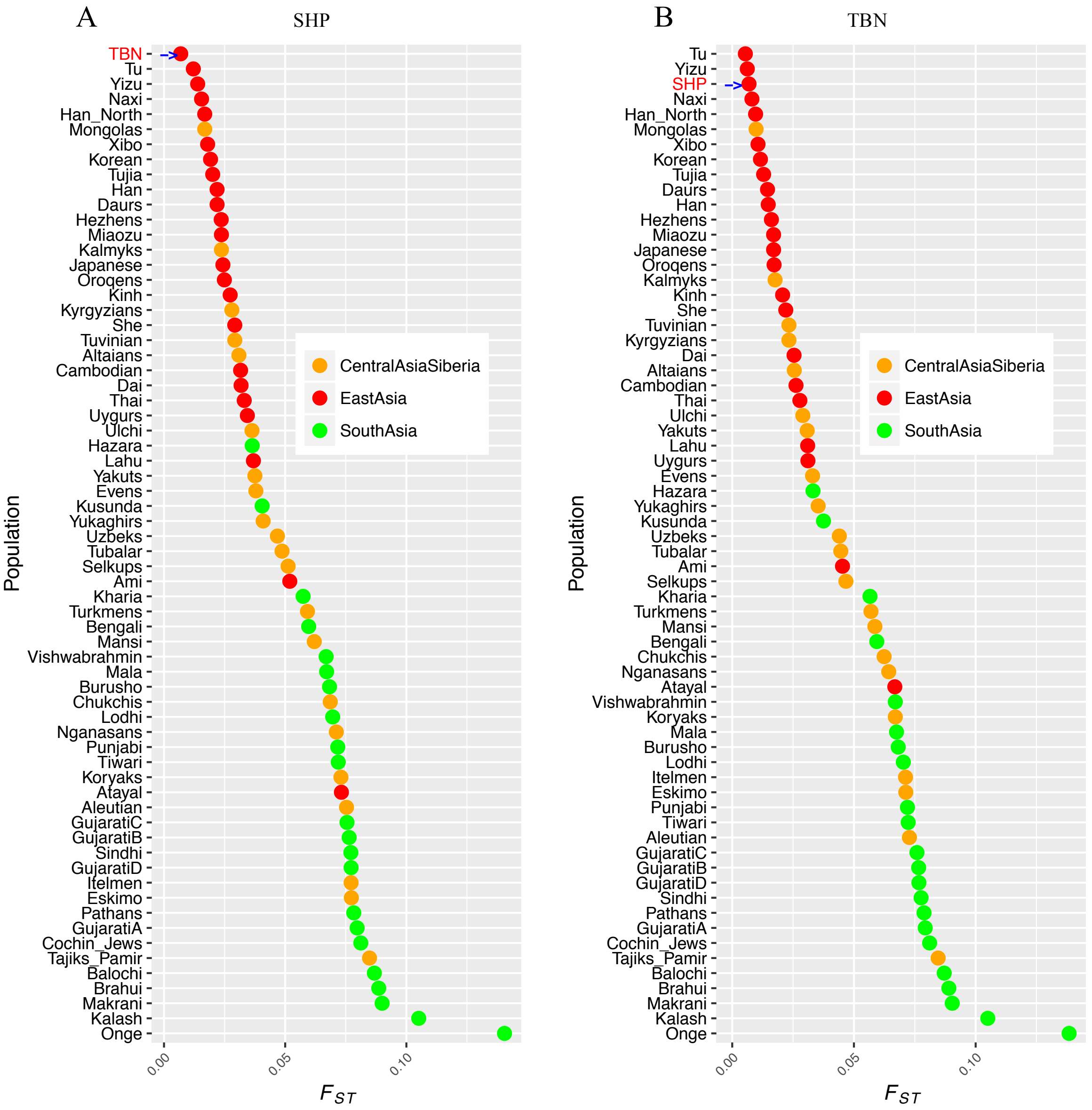

Figure S4 ***F_{ST}* between regional SHP and populations from East Asians, Central Asian/Siberians and South Asians.** The analysis was based on panel 2 since more SHP.Khumbu individuals were included in it. To exclude the influence of sample size on the result, 9 individuals from each TBN and SHP subgroup were included.

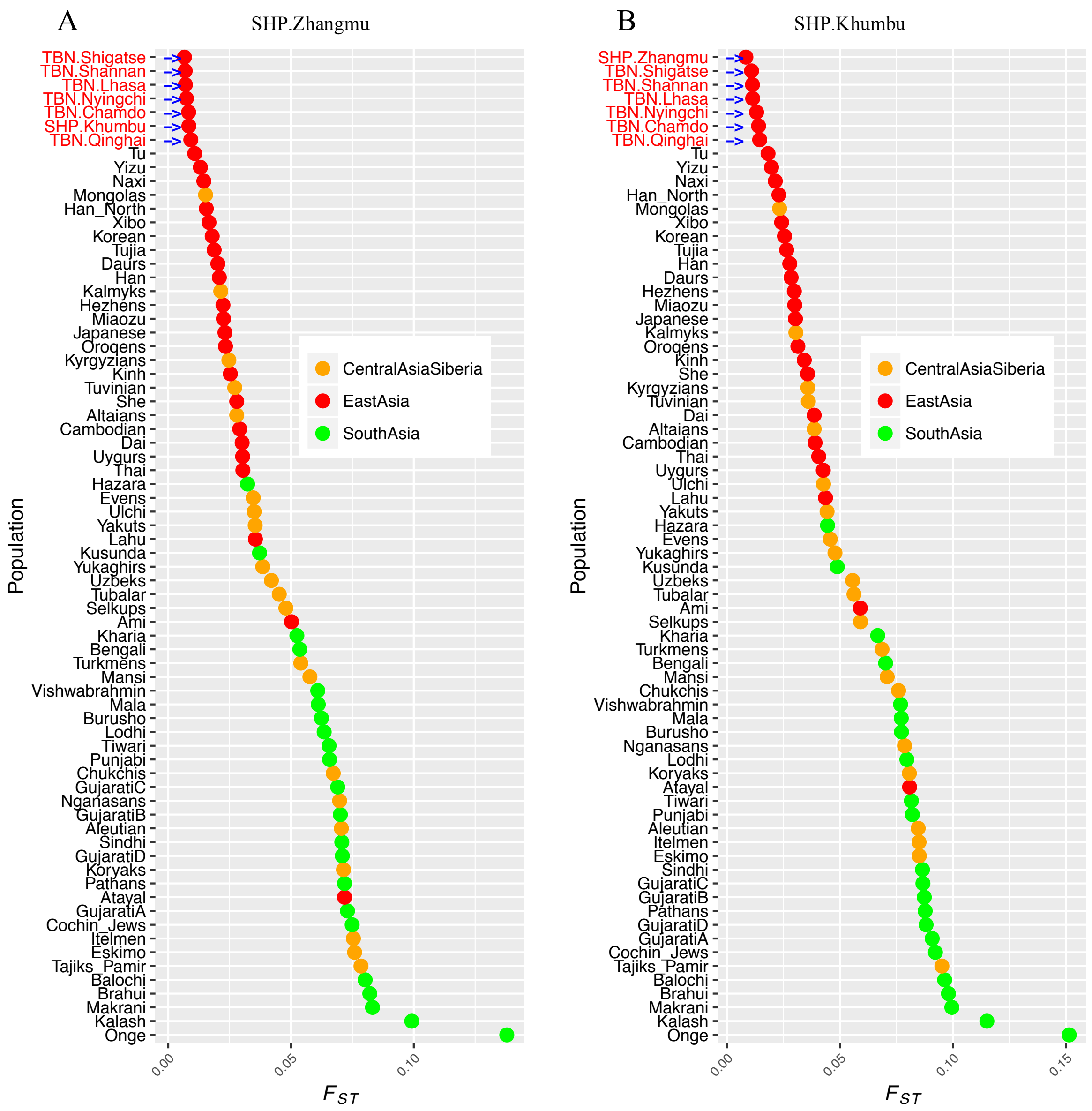

Figure S5 **F_{ST} between regional TBN and populations from East Asians, Central Asian/Siberians and South Asians.** The analysis was based on panel 2 since more SHP.Khumbu individuals were included in it. To exclude the influence of sample size on the result, 9 individuals from each TBN and SHP subgroup were included. The Ü-Tsang West Tibetans (TBN.Shigatse, TBN.Shannan and TBN.Lhasa) are more genetically related, while Kham and Amdo Tibetans (TBN.Nyingchi, TBN.Chamdo and TBN.Qinghai) have more genetic affinities.

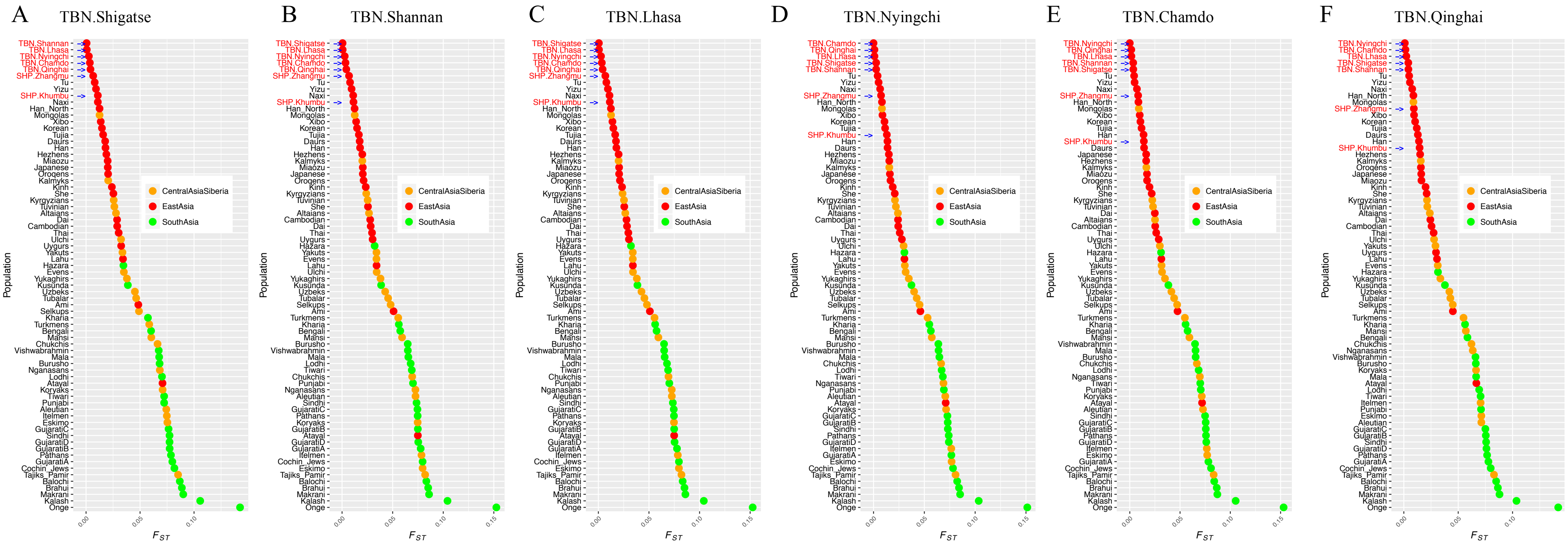

Figure S6 **Panel 1 dataset-based outgroup f_3 tests in the form of (A, B) f_3(SHP; Yoruba, X) and (C, D) f_3(TBN; Yoruba, X) to detect the genetic affinities of SHP and TBN.** Larger f_3 value indicates two populations shared more genetic history or are more genetically related. In A and C, each dot represents one specific population X located in its geographic region and the color of the dot denotes the f_3 score between population X and SHP (or TBN). The wormer the color is, the more genetic history X and SHP (or TBN) share as shown in the heat key. B and D show the rank of the f_3 scores. SHP or TBN was highlighted by its font color and by the corresponding blue arrow.

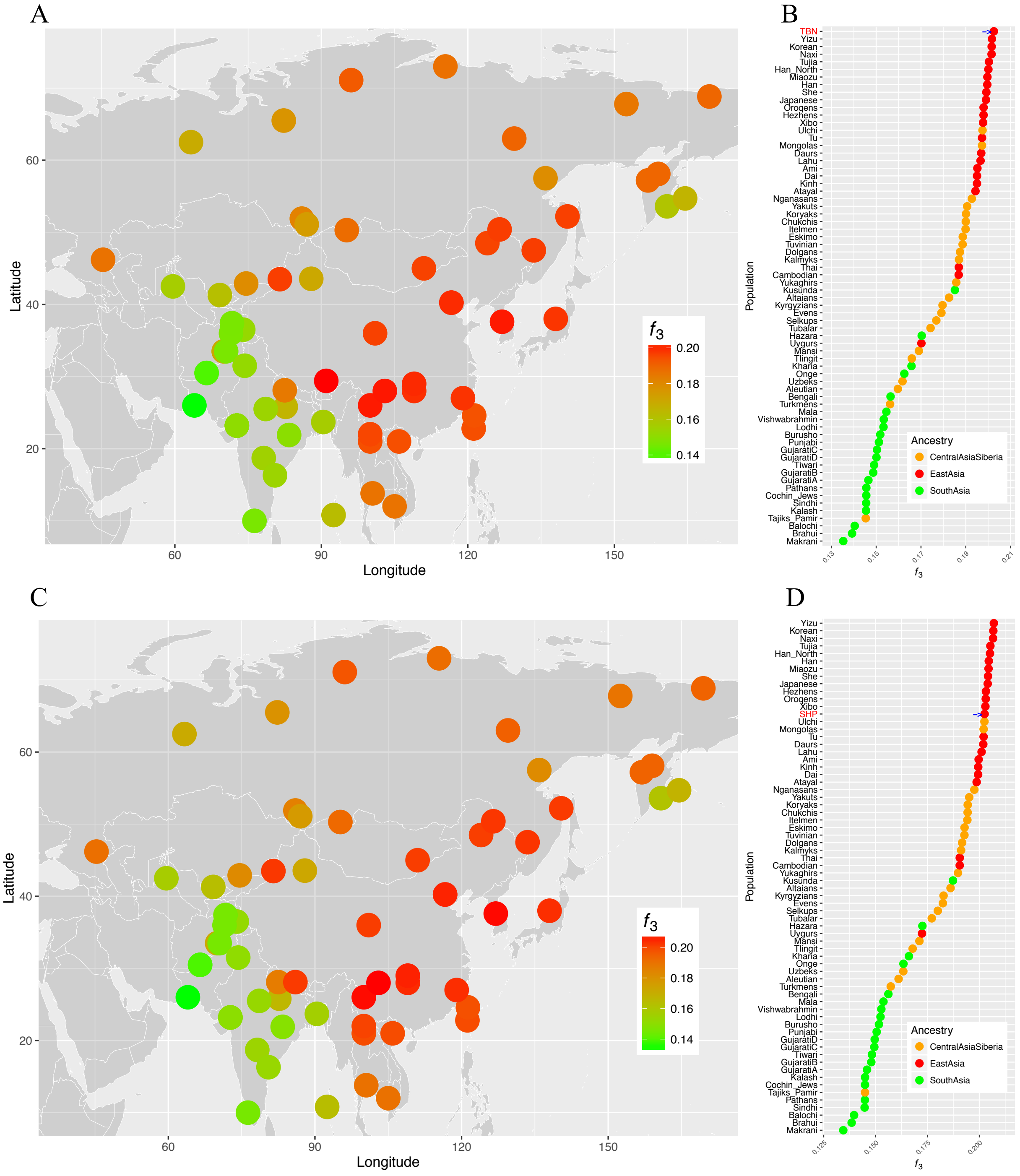

Figure S7 Panel 2 dataset-based outgroup f_3 tests in the form of (A, B) f_3(SHP; Yoruba, X) and (C, D) f_3(TBN; Yoruba, X) to detect the genetic affinities of SHP and TBN. Larger f_3 value indicates two populations shared more genetic history or are more genetically related. In A and C, each dot represents one specific population X located in its geographic region and the color of the dot denotes the f_3 score between population X and SHP (or TBN). The wormer the color is, the more genetic history X and SHP (or TBN) share as shown in the heat key. B and D show the rank of the f_3 scores. SHP or TBN was highlighted by its font color and by the corresponding blue arrow.

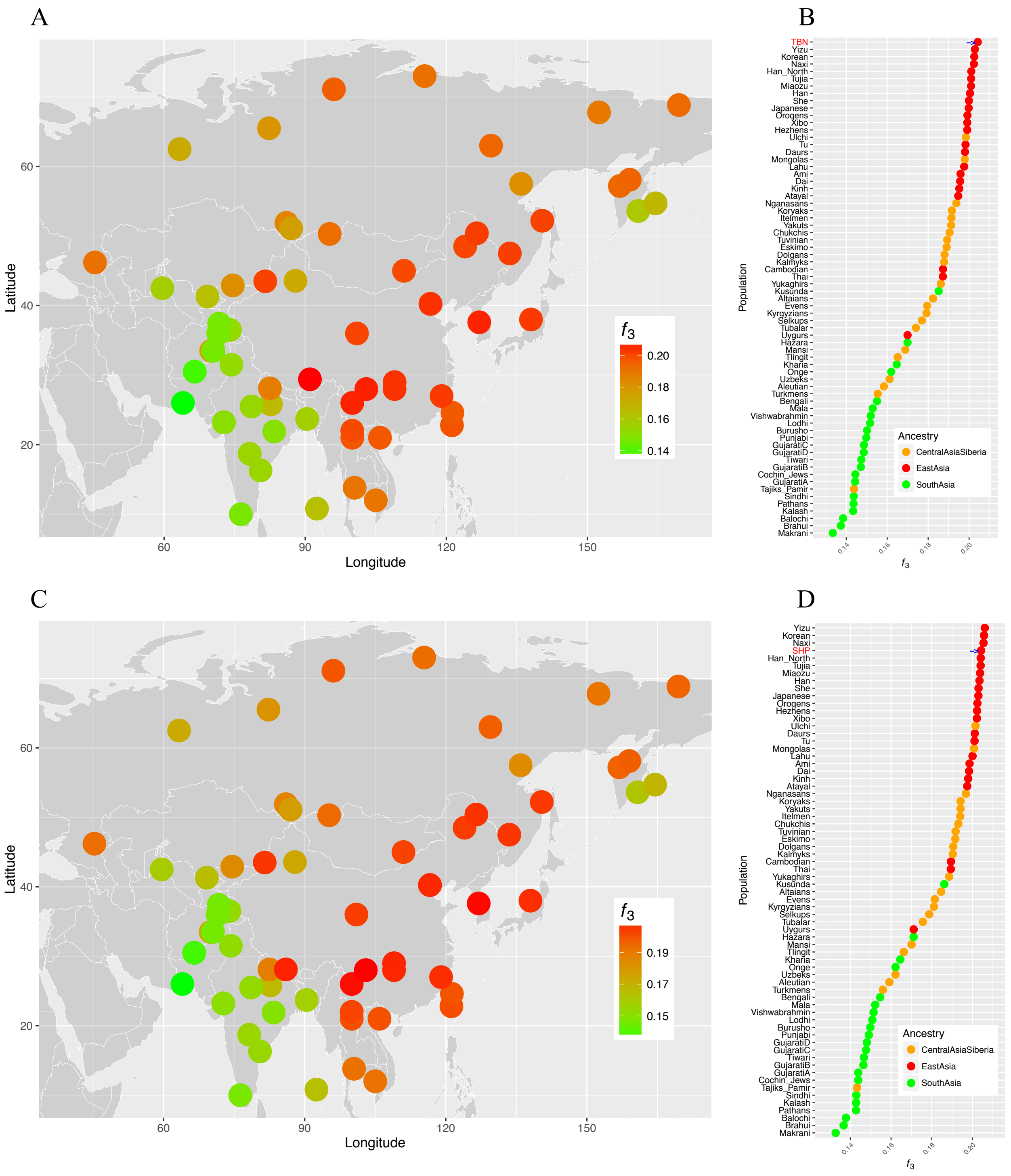

Figure S8 Outgroup f_3 test in the form (A, C) f_3(SHP.Zhangmu; Yoruba, X) and (B, D) f_3(SHP.Khumbu; Yoruba, X). X here are populations from East Asians, Central Asian/Siberians and South Asians. (A, B) and (C, D) are based on Panel 1 and Panel 2, respectively. SHP, TBN or their subgroup was highlighted by its font color and by the corresponding blue arrow. In A, B and C, SHP.Zhangmu or SHP.Khumbu shows higher genetic affinities with another Sherpa subgroup, followed by Tibetan subgroups. In D, though the f_3 score between SHP.Khumbu and SHP.Zhangmu is not the largest, possibly indicating the distinct population history after their diversification.

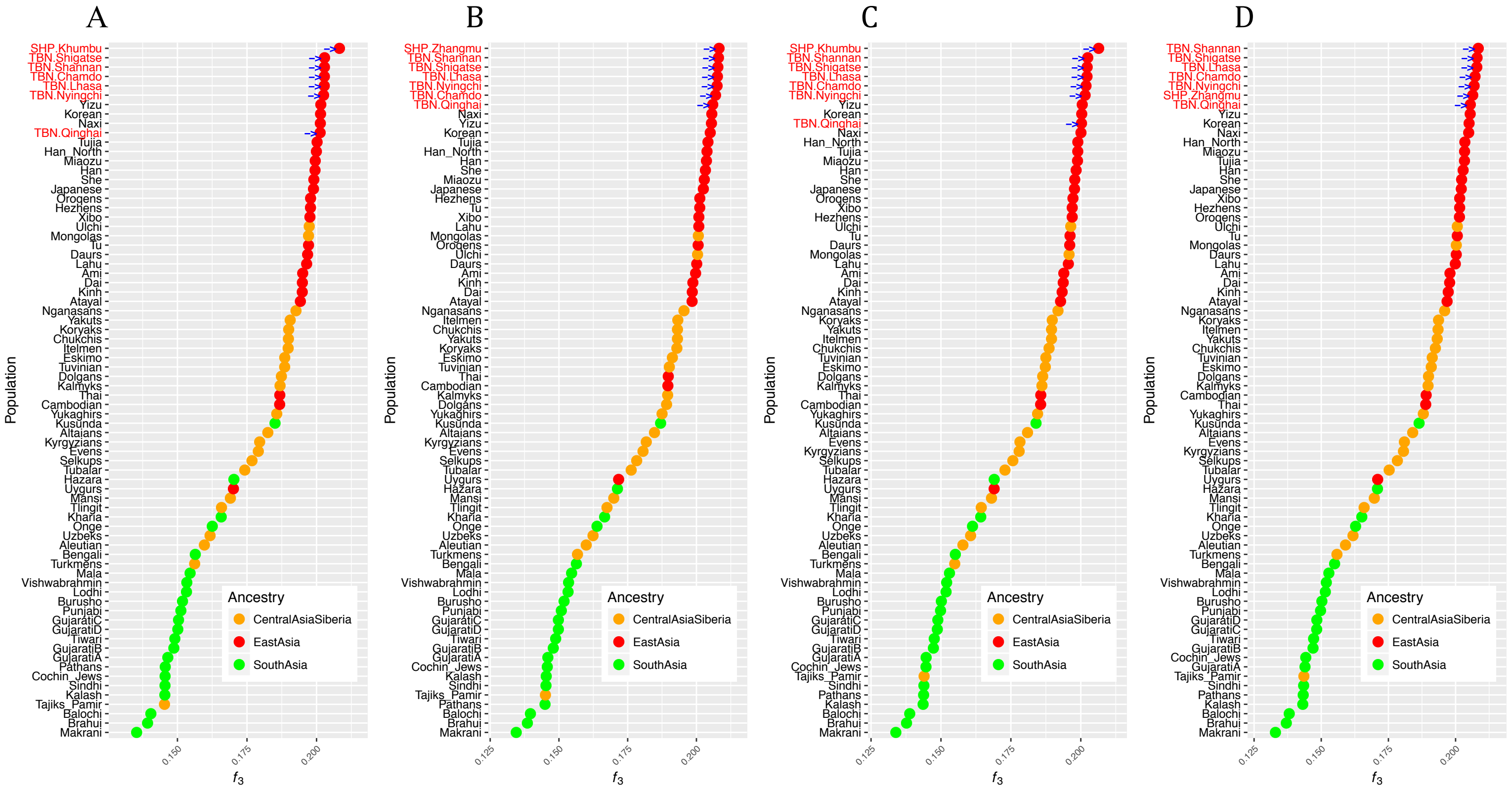

Figure S9 Panel 1 dataset-based outgroup f_3 test in the form f_3(TBN.region; Yoruba, X). TBN.region represents one of the Tibetan subgroup and X denotes population of East Asians, Central Asian/Siberians or South Asians. SHP, TBN or their subgroup was highlighted by its font color and by the corresponding blue arrow.

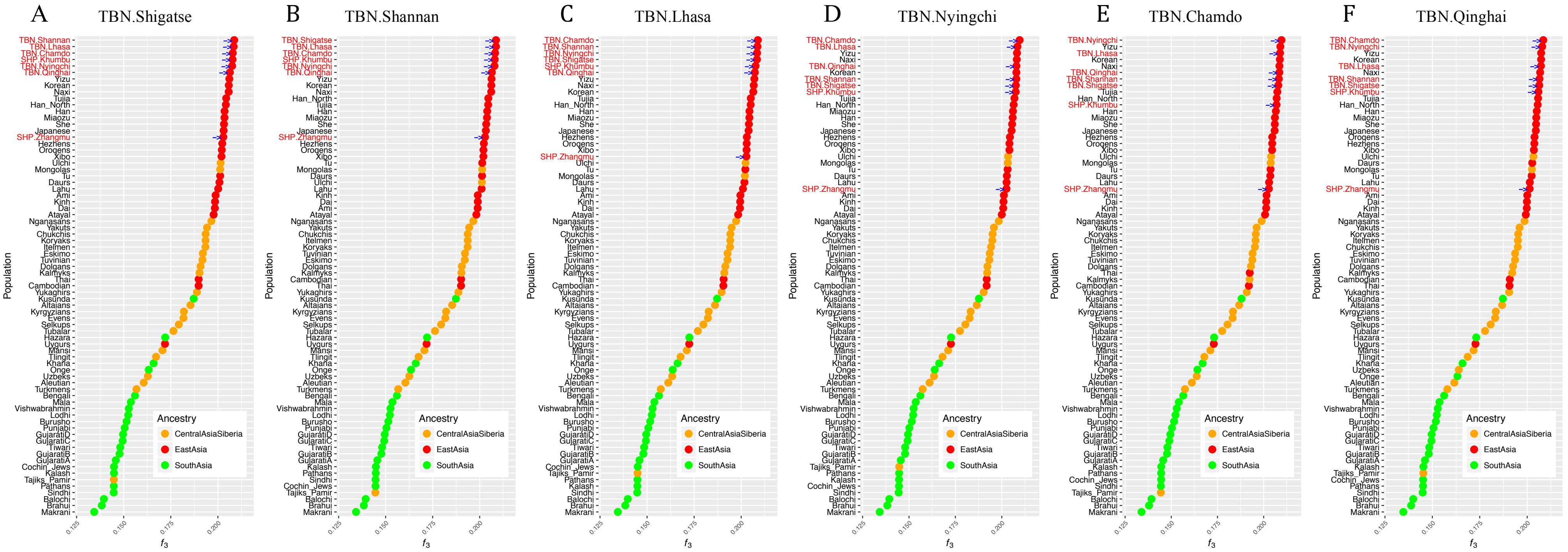

Figure S10 Panel 2 dataset-based Outgroup f_3 test in the form f_3(TBN.region; Yoruba, X). TBN.region represents one of the Tibetan subgroup and X denotes population of East Asians, Central Asian/Siberians or South Asians. SHP, TBN or their subgroup was highlighted by its font color and by the corresponding blue arrow.

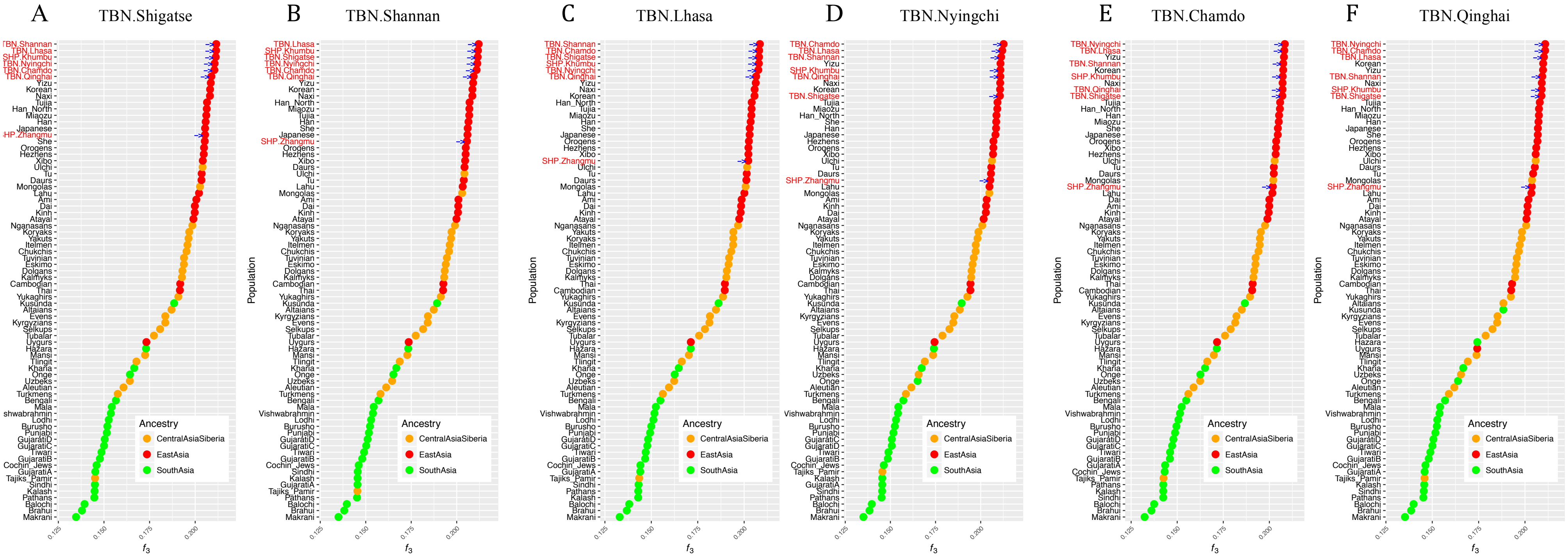

Figure S11 Principal component analysis (PCA) of SHP and TBN in the contexts of worldwide population samples. Analysis of the first two principal components of SHP and TBN in Panel 1 within the context of world-wide population samples which are consist of 2,035 Human Origin samples (A), removing Africans (B) and removing Africans, West Eurasians and the Oceanian(C). Geographical regions where the individuals are located are indicated with colors as shown in the legend. Numbers in brackets denote variance explained by each principal component (PC). The same analyses were also performed in Panel 2 with corresponding figures shown in (D), (E) and (F), respectively.

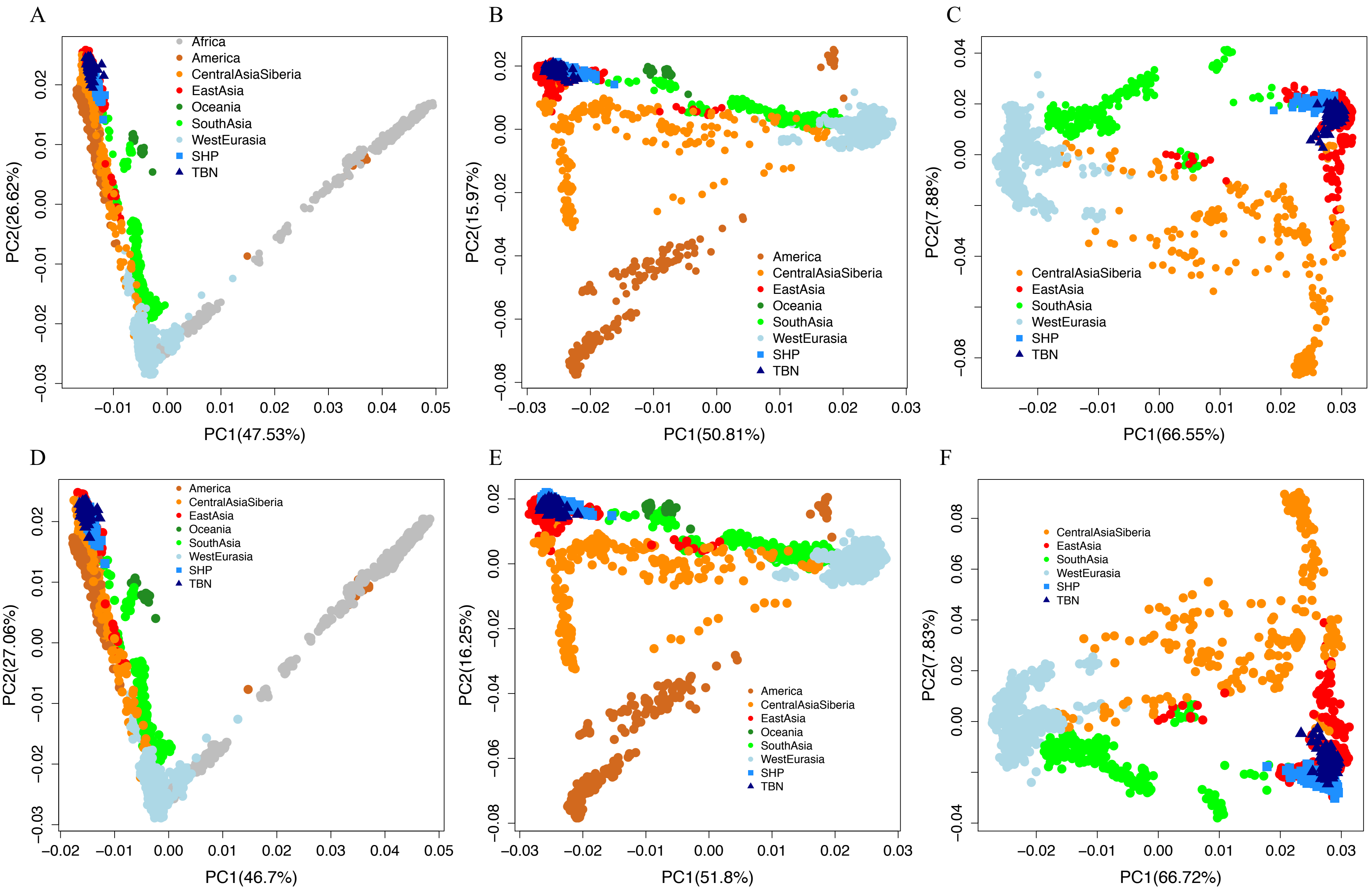

Figure S12 PCA of SHP and TBN within the context of some East Asian and South Asian samples. (A) and (B) indicate the PCA of SHP and TBN with the context of some East Asians and South Asians for Panel 1 and 2 respectively. (C) Panel 1 dataset-based PCA of SHP and TBN with the context of some East Asians and (D) Panel 1 dataset-based PCA with only the two highlanders were included. Population labels of the individuals are indicated with colors as shown in the legend. The subgroups of SHP and TBN are classified according to their geographic locations. Numbers in brackets denote variance explained by each PC.

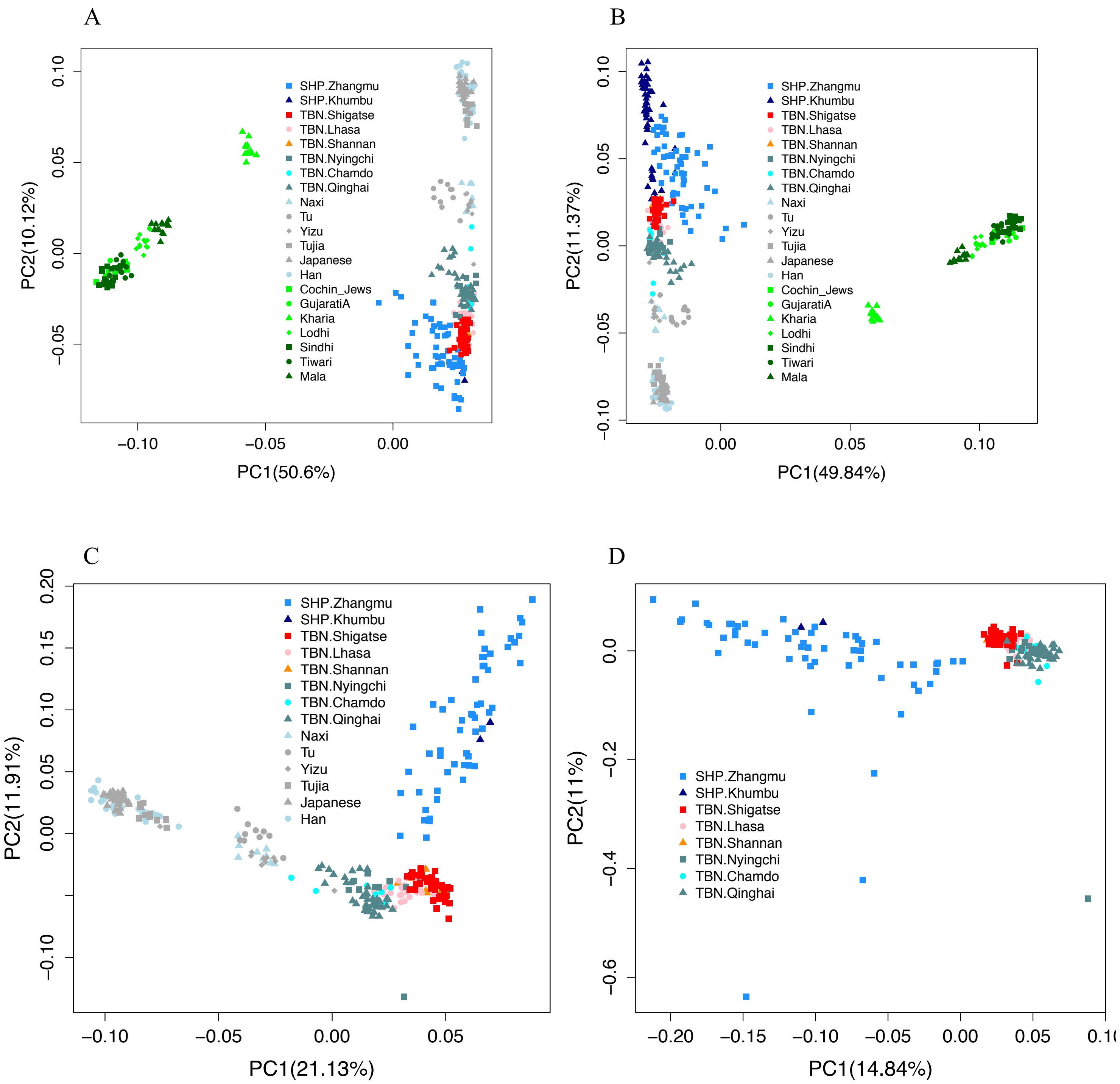

Figure S13 PCA of SHP and TBN with SHPseq and SHPseq2 are included. The subgroups of SHP are classified according to their geographic locations. Population labels of the individuals are indicated with colors as shown in the legend. Numbers in brackets denote variance explained by each PC. The PCA is based on Panel 2 for the lager sample size of Nepalese Sherpa (SHP.Khumbu) in this Panel. Totally, eight sequenced Sherpas were included. Three individuals from Chinese Sherpas and two from Nepalese Sherpas were both genotyped by array and by next generation sequencing (NGS) (Each red circle indicates the same individual generated the different technology). The array data and NGS data accord well, suggesting the differences between SHP.Zhangmu and SHP.Khumbu possibly not be result from batch effect though Affymetrix and Illumina platforms were used, respectively.

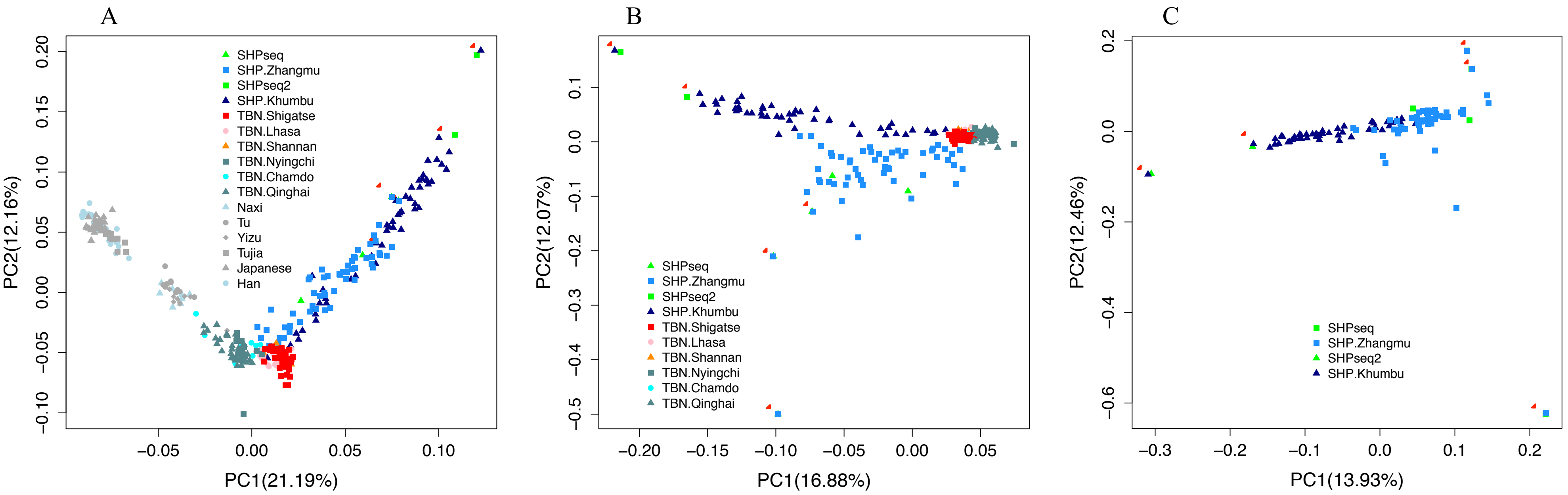

Figure S14 Estimation of batch effect in the influence of population substructure in TBN according to PCA. Population labels of the individuals are indicated with colors as shown in the legend. Numbers in brackets denote variance explained by each PC. The individuals and SNPs used here are the same with Figure 2D except that population labels are based on dataset sources. TBN.Current denotes TBN samples from Xu *et al*(1) as well as this current study; TBN.Simonson for sample from Simonson *et al* (2) and TBN.Peng for samples from Peng *et al* (3) (Table S1). In the PCA, three line of evidences show that the population substructure observed in TBN possibly are not induced by batch effect. Firstly, the samples do not split by different data sources. Secondly, individuals from different dataset clustered together according to their geographic locations or cultural regions. For instance, Qinghai Tibetans from TBN.Peng and TBN.Simonson make up a clade (purple shadow). Lastly, individuals from same dataset split well by their cultural regions, such as TBN.Current which comprises 5 subgroups (TBN.Shigatse, TBN.Shannan, TBN.Lhasa, TBN.Nyingchi and TBN.Chamdo) (Table S1). Therefore, we speculate little influence of batch effect on the population substructure observed in TBN.

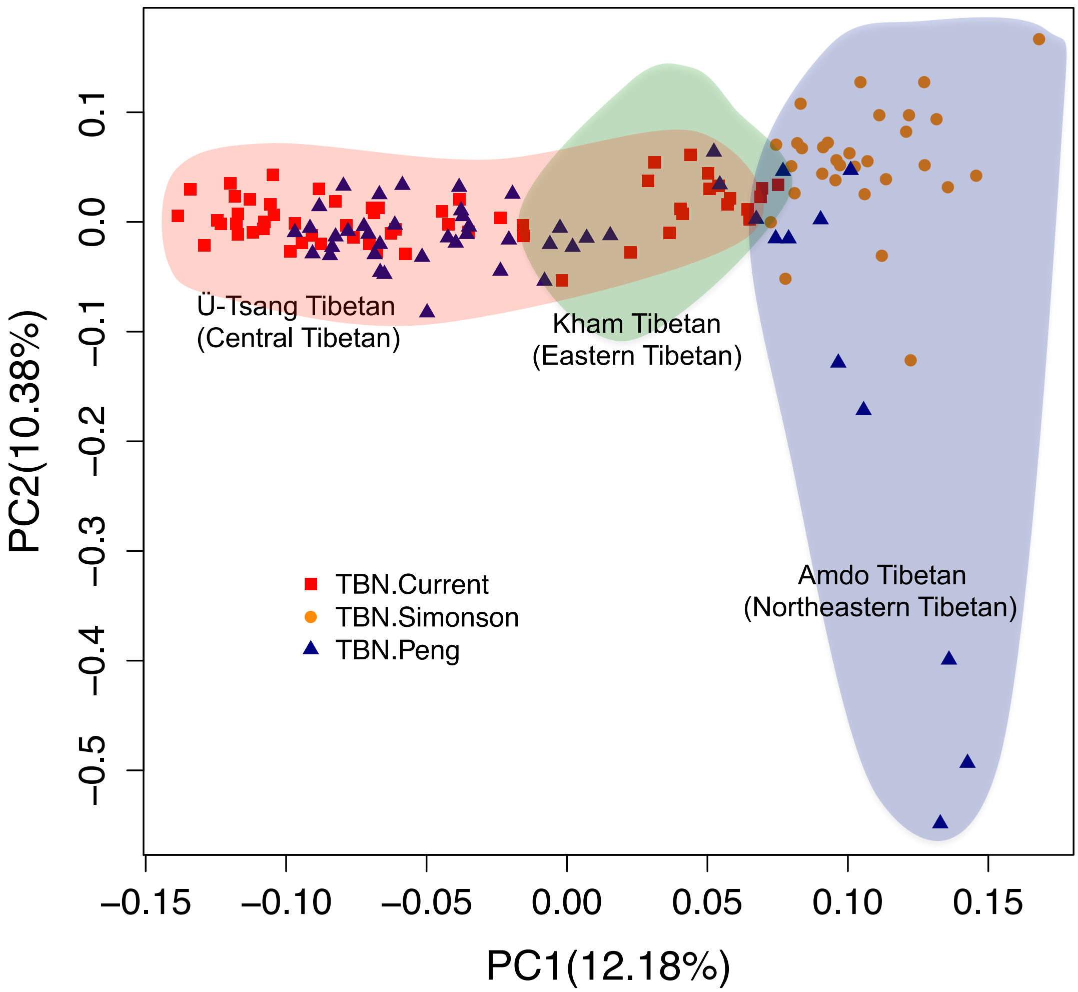

Figure S15 **Panel 1 dataset-based summary plot for *ADMIXTURE* of East Asians, Central Asian/Siberians and South Asians.** **Each individual is represented by a single vertical line broken into *K* colored segments with lengths proportional to each of the K inferred clusters, where the *K* values are shown in the legend. The predefined populations IDs are presented at the bottom of the plot**.

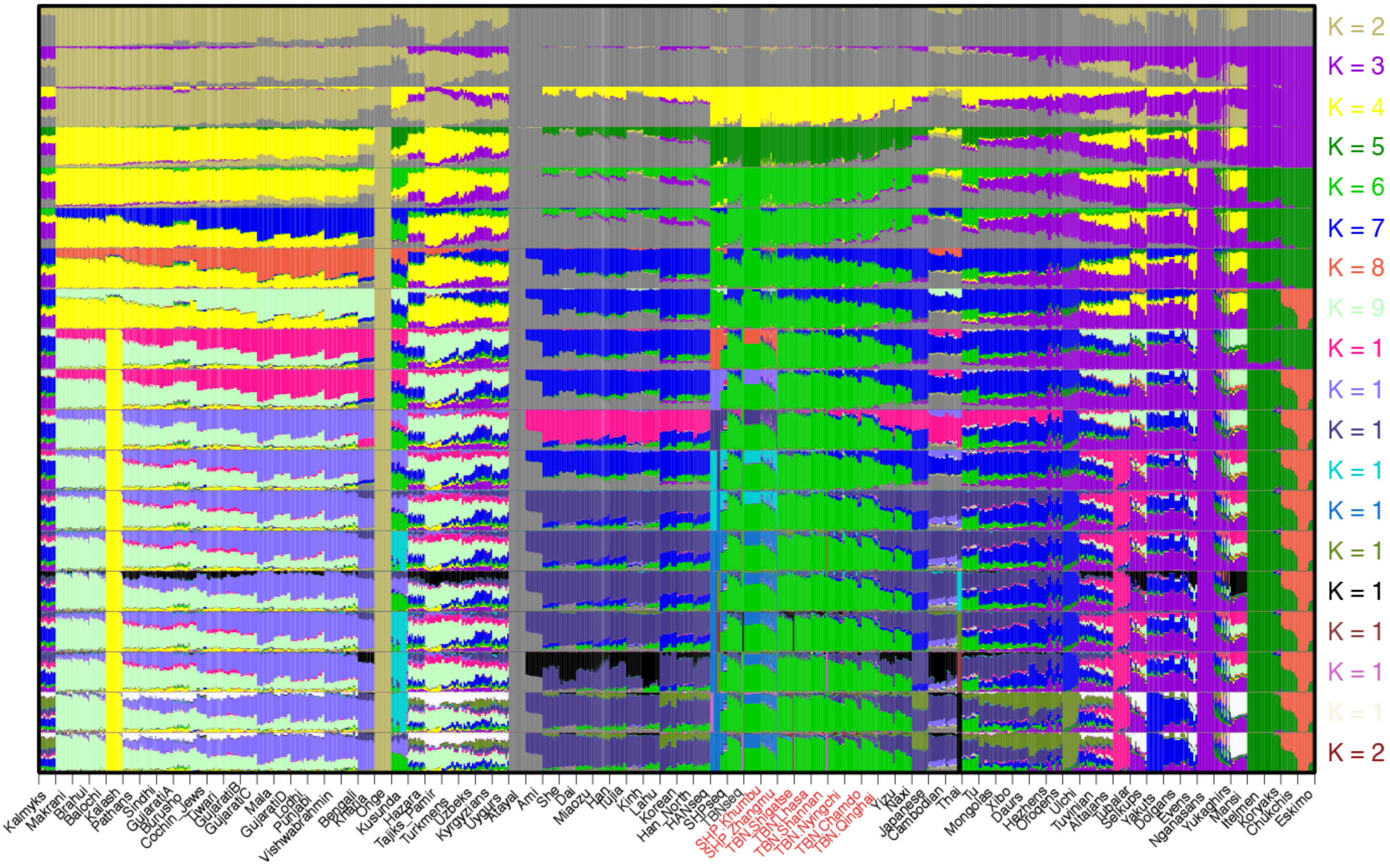

Figure S16 **Panel 2 dataset-based summary plot for** ***ADMIXTURE*** of East Asians, Central Asian/Siberians and South Asians. **Each individual is represented by a single vertical line broken into K colored segments with lengths proportional to each of the K inferred clusters, where the K values are shown in the legend. The predefined populations IDs are presented at the bottom of the plot**.

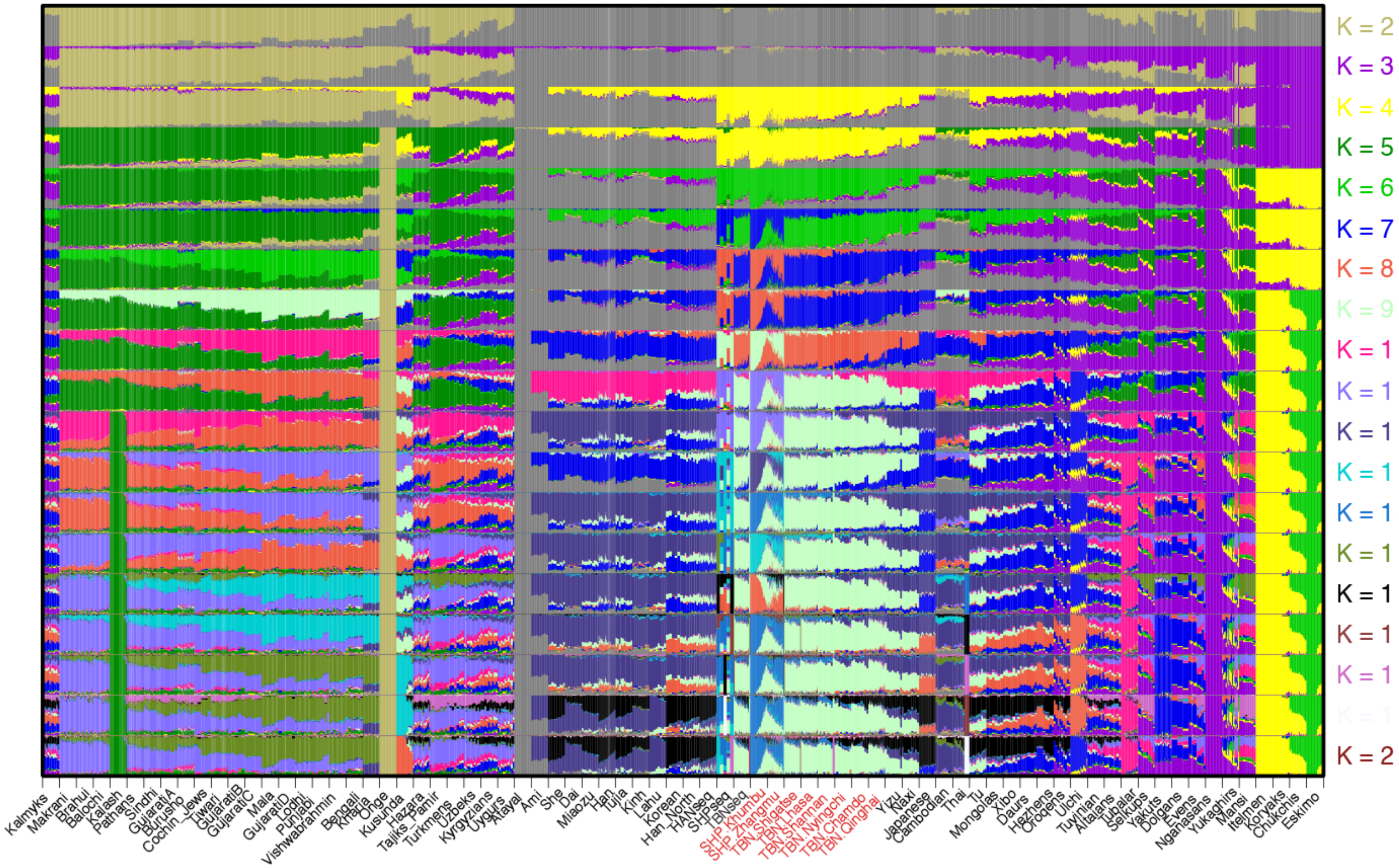

Figure S17 **Panel 1 dataset-based *ADMIXTURE*** of selected some East Asians, Central Asian/Siberians and South Asians. **Each individual is represented by a single vertical line broken into K colored segments with lengths proportional to each of the K inferred clusters, where the K values are shown in the legend. The predefined populations IDs are presented at the bottom of the plot**.

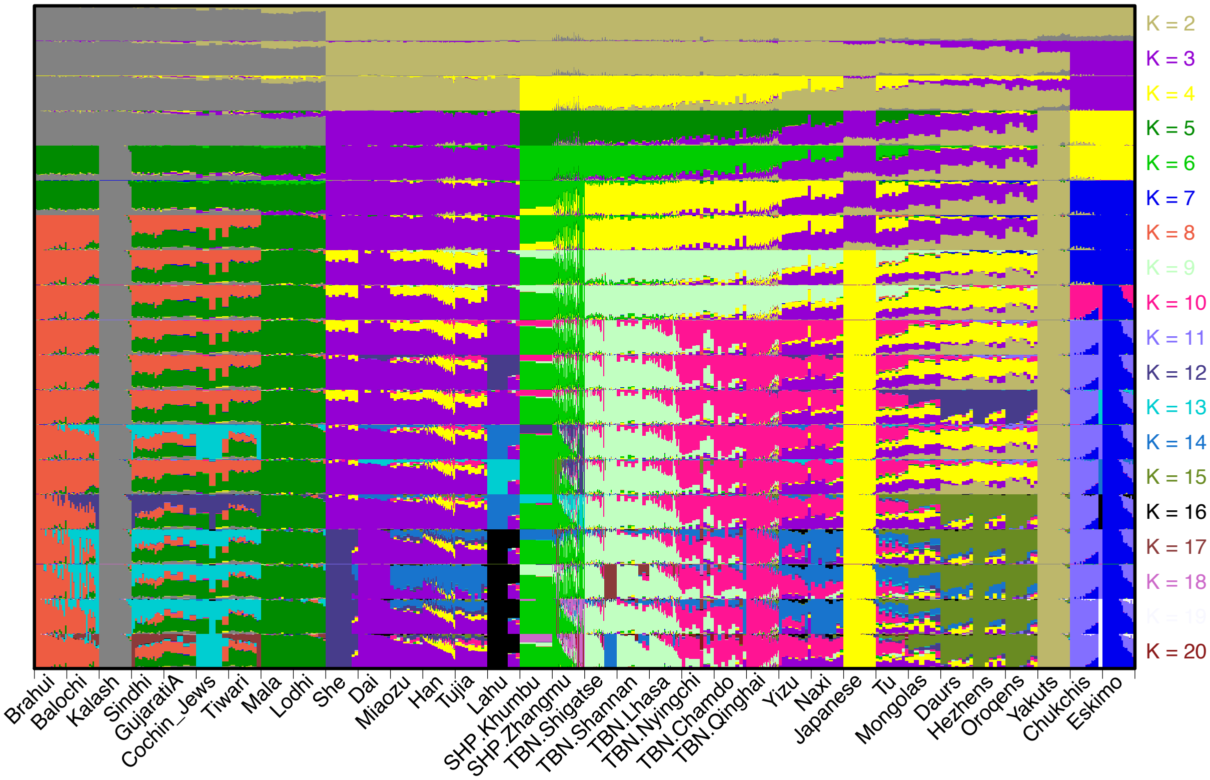

Figure S18 Panel 2 dataset-based *ADMIXTURE* of selected some East Asians, Central Asian/Siberians and South Asians. Each individual is represented by a single vertical line broken into K colored segments with lengths proportional to each of the K inferred clusters, where the K values are shown in the legend. The predefined populations IDs are presented at the bottom of the plot.

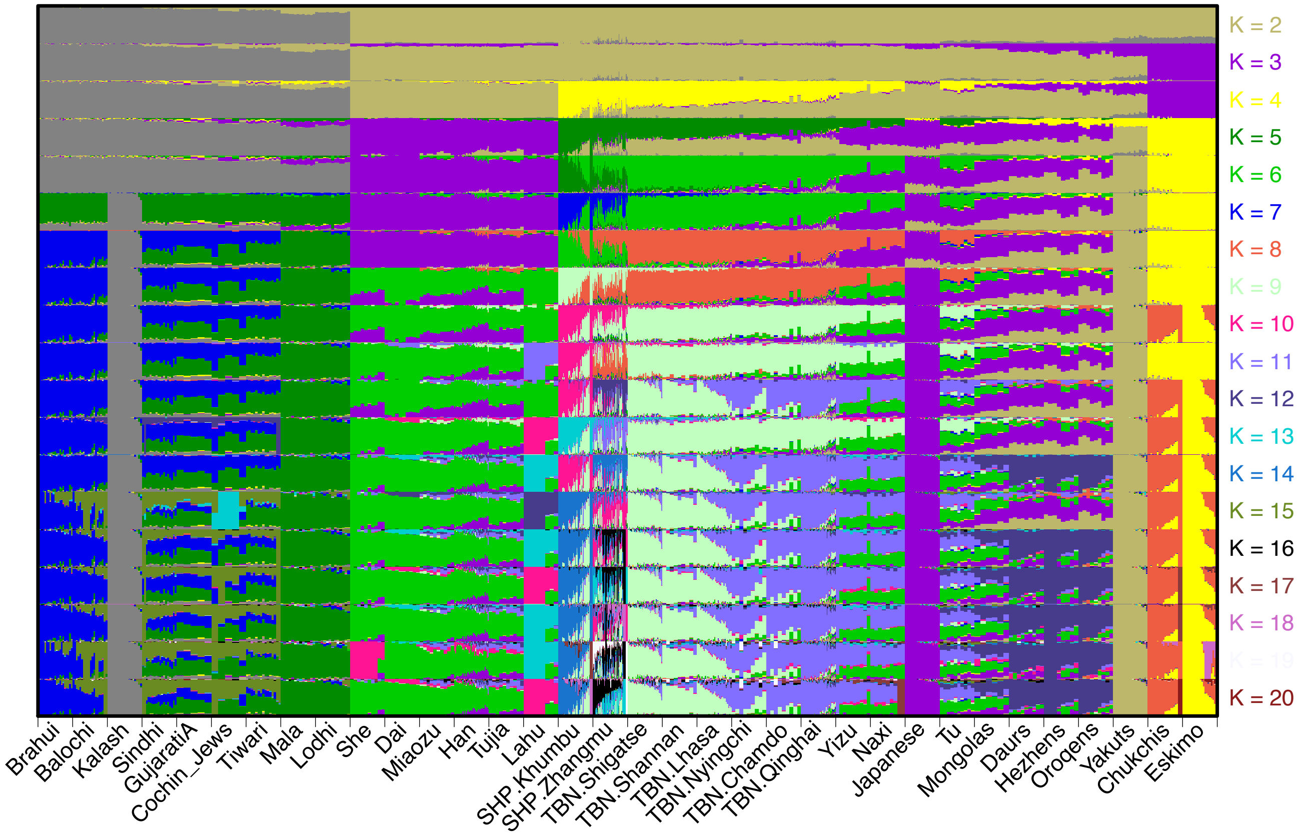

Figure S19 The cross validation error estimated by *ADMIXTURE.* The analysis is based on Figure S17 and S18. In both panels, 4, 5 and 6 are best Ks due to their small cross validation errors.

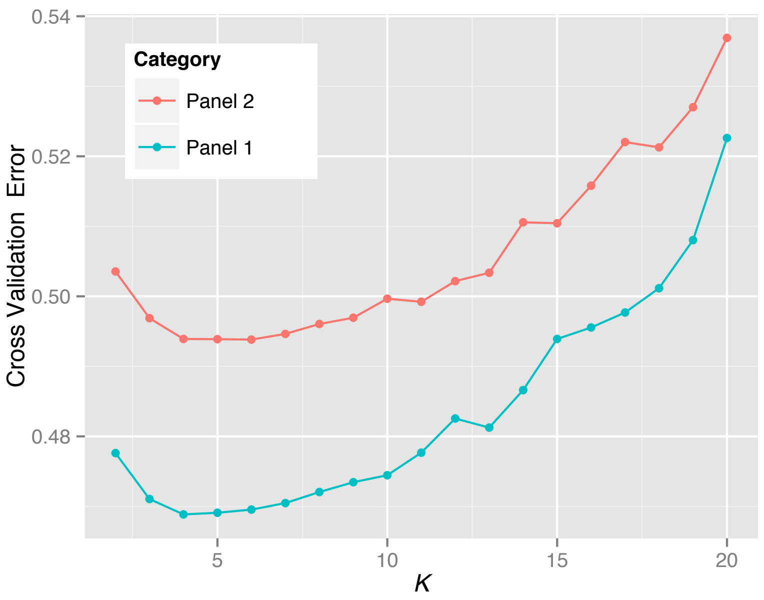

Figure S20 Panel 1 dataset-based result of genetic admixture when assuming K=7. Each individual is represented by a single line broken into K=6 colored segments, with lengths proportional to the K=6 inferred clusters. The population IDs are presented outside of the circle of the plot. The results of population level admixture of all SHP and TBN are further summarized and displayed in the two big pie charts in the center of the circle plot with admixture proportion denoted in percentage. Admixture proportions for SHP and TBN subgroups are summarized in the small pie charts with their proportions values listed below.

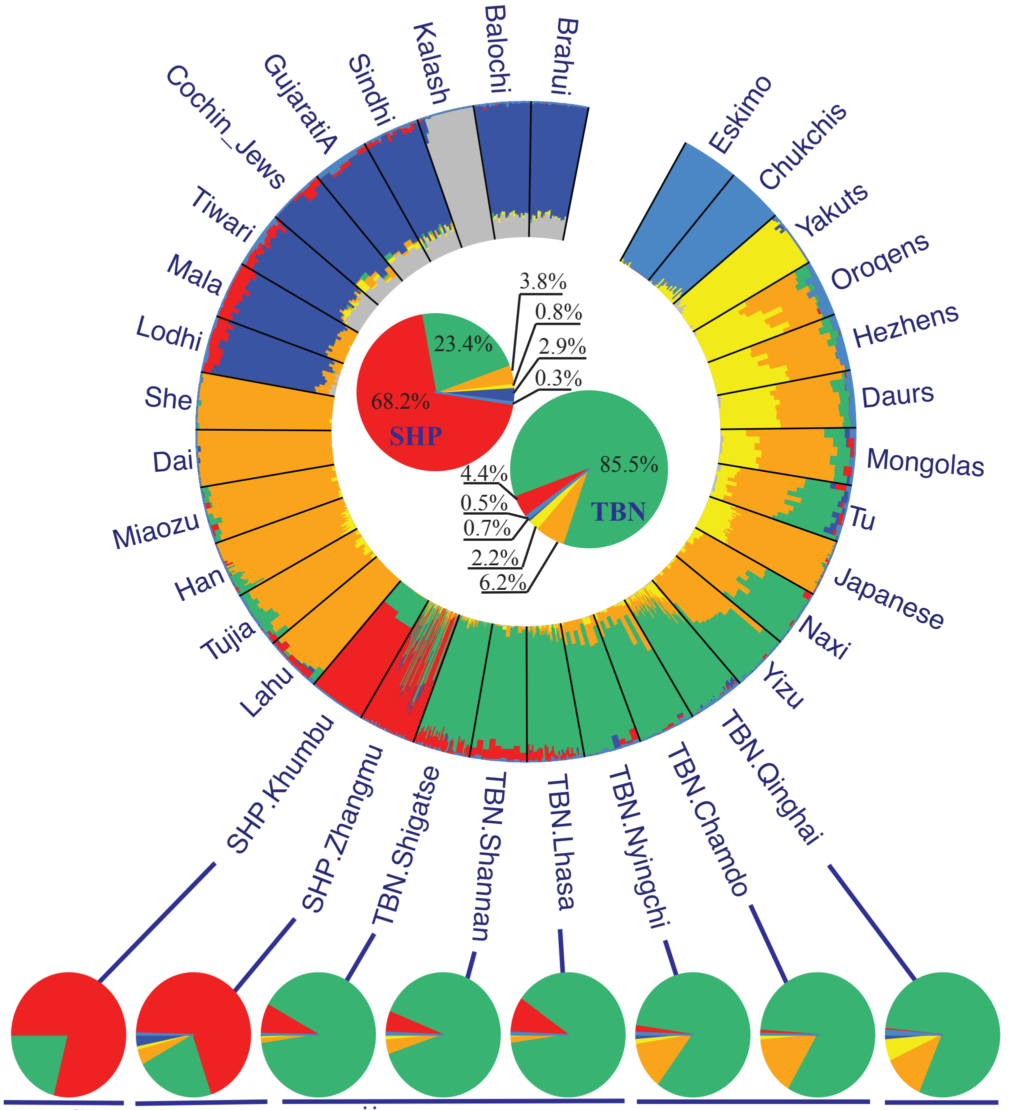

	Nepalese SHP	Chinese SHP	Ü-Tsang TBN			Kham TBN		Amdo TBN
TC	21.96	23.49	89.20	88.85	88.08	83.05	81.76	79.64
SC	77.88	67.94	7.95	5.07	8.77	1.51	0.55	0.26
EAC	0.16	3.93	1.43	3.89	1.81	12.23	15.53	11.88
SC1	0.00	0.82	0.41	0.99	0.33	1.34	1.57	5.60
SC2	0.00	0.40	0.39	0.70	0.56	0.60	0.39	1.22
SAC	0.00	3.07	0.44	0.35	0.02	1.15	0.13	0.94

Figure S21 The total length of (A) short, (B) median and (C) long run of homozygosity (ROH) in SHP, TBN and their subgroups. To measure consanguinity, ROH was calculated by PLINK. The sliding widow size is 500kb with at lest 50 SNPs and one heterozygote, five missing calls are allowed per widow. If the proportion overlapping between two windows was > 0.05, two ROHs were merged. Then we classified ROH into short, median and long classes using Mclust in R. Compared with TBN, SHP shows significant ROH for short, median and long ROH classes with P-value of 3.35e-08, 1.40e-10 and 2.2e-16 respectively. For SHP subgroups, the ROH segments of SHP.Khumbu is significantly higher than SHP.Zhangmu with P-value of 4.70e-05, 1.78e-05, and 0.005 for short, median and long ROH classes respectively. Little differences were observed between TBN subgroups.

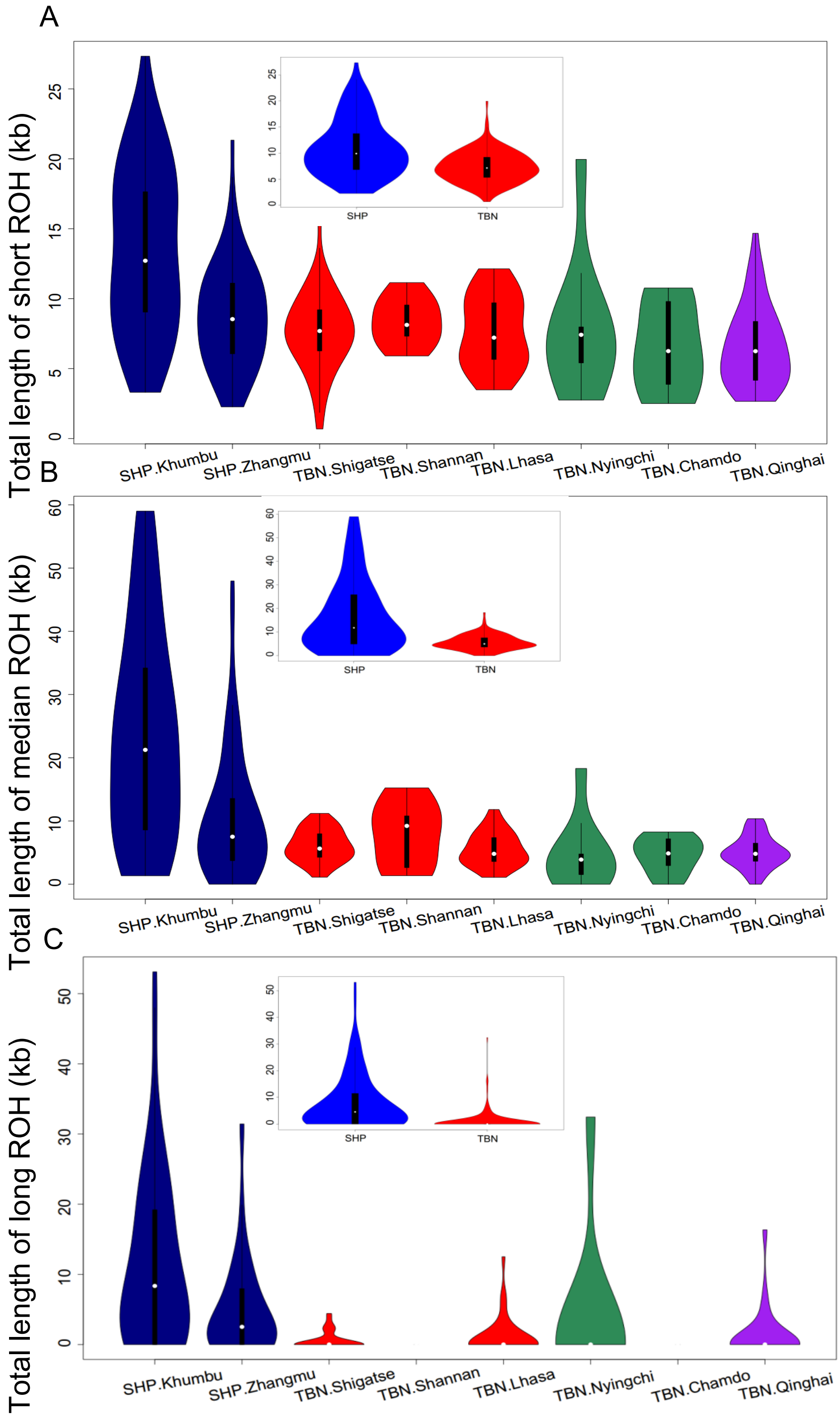

Figure S22 Panel 2 dataset-based f_3 tests of the form (A) f_3(TBN; SHP, X) and (B) f_3(SHP; TBN, X). X represents East Asian, Central Asian/Siberian and South Asian populations. SHP and TBN are enrolled all SHP and TBN individuals in Panel 2, that is 104 SHP and 142 TBN (See table S1). Symbol '**' for Z scores ≤ -3 and '*' for -3 < Z ≤-2 in the test.

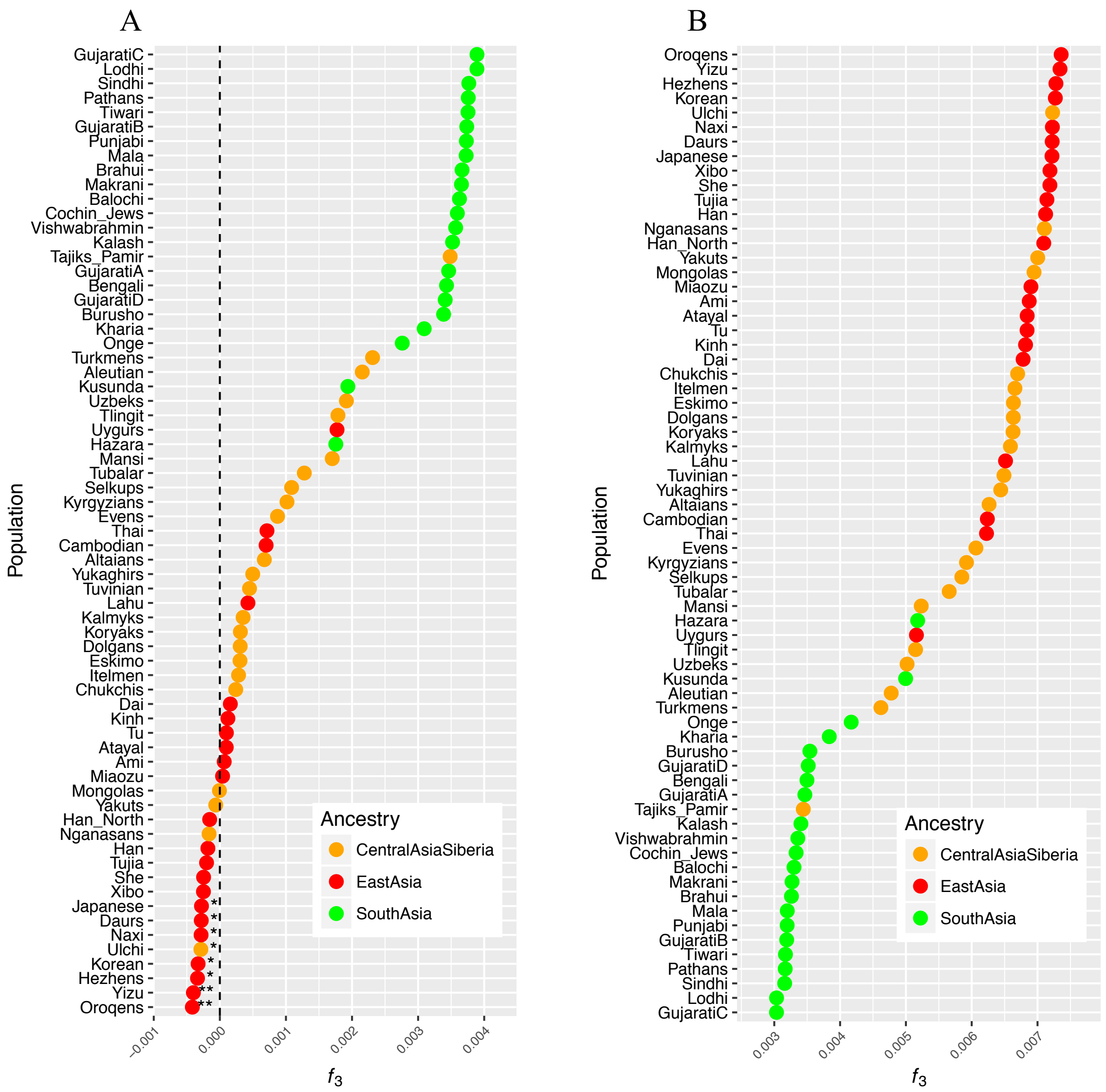

Figure S23 Panel 2 dataset-based f_3 tests to detect the gene flow from TBN subgroups to SHP subgroups in the forms (A) f_3(SHP.Zhangmu; TBN.subgroup, X) and (B) f_3(SHP.Khumbu; TBN.subgroup, X). The forms of the tests were listed on the top of each figure. Strong gene flow from TBN.Shigatse and X to SHP.Zhangmu were detected. No gene flows were observed from TBN subgroups to SHP.Khumbu. Symbol '**' for Z scores $\leq$ -3 and '*' for -3 < Z $\leq$-2 in the test.

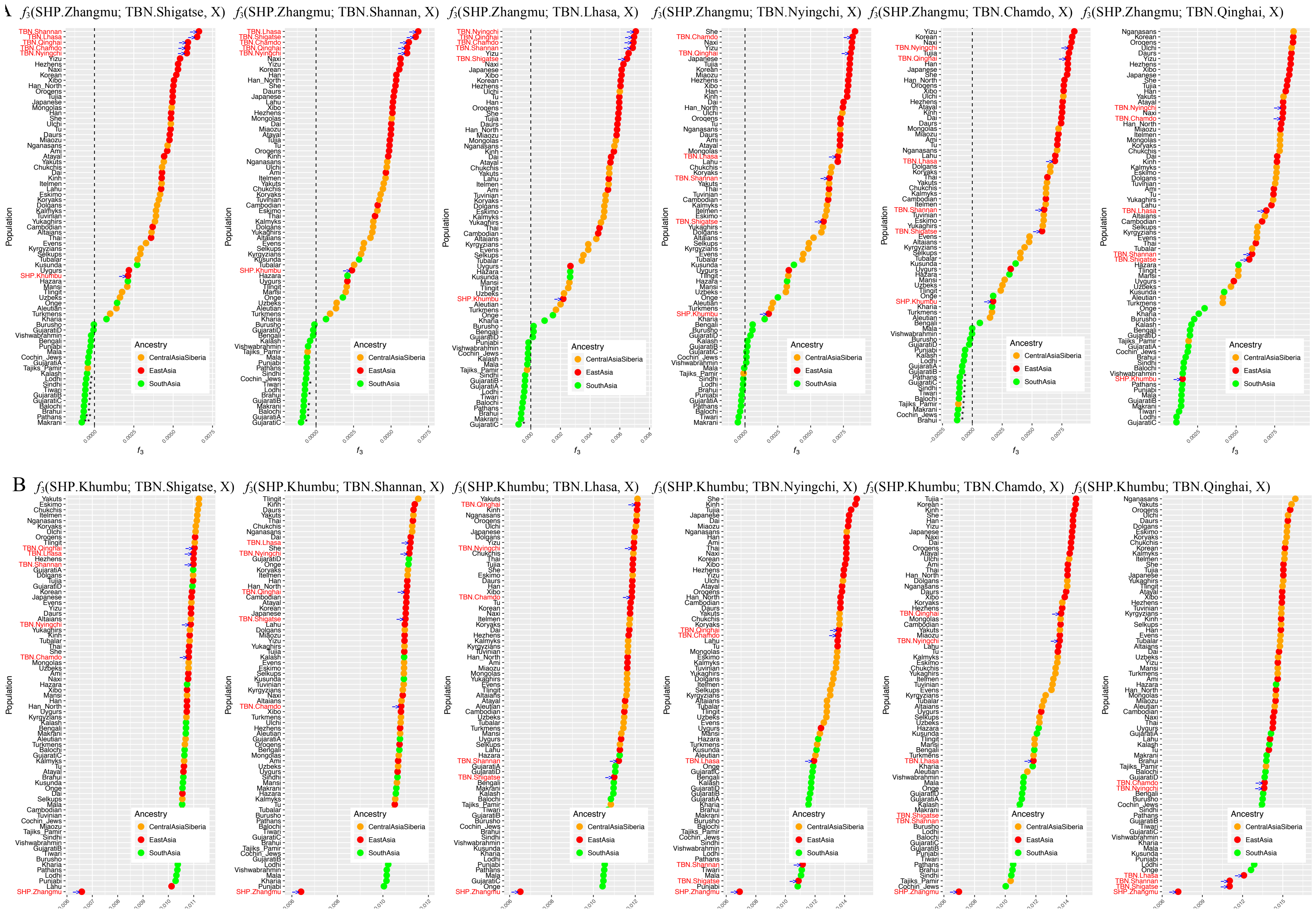

Figure S24 **Panel 2 dataset-based f_3 tests to detect the gene flow from SHP subgroups to TBN subgroups in the forms (A) f_3(TBN.subgroup; SHP.Zhangmu, X) and (B) f_3(TBN.subgroup; SHP.Khumbu, X).** The forms of the tests were listed on the top of each figure. Symbol '**' for Z scores ≤ -3 and '*' for -3 < Z ≤-2 in the test.

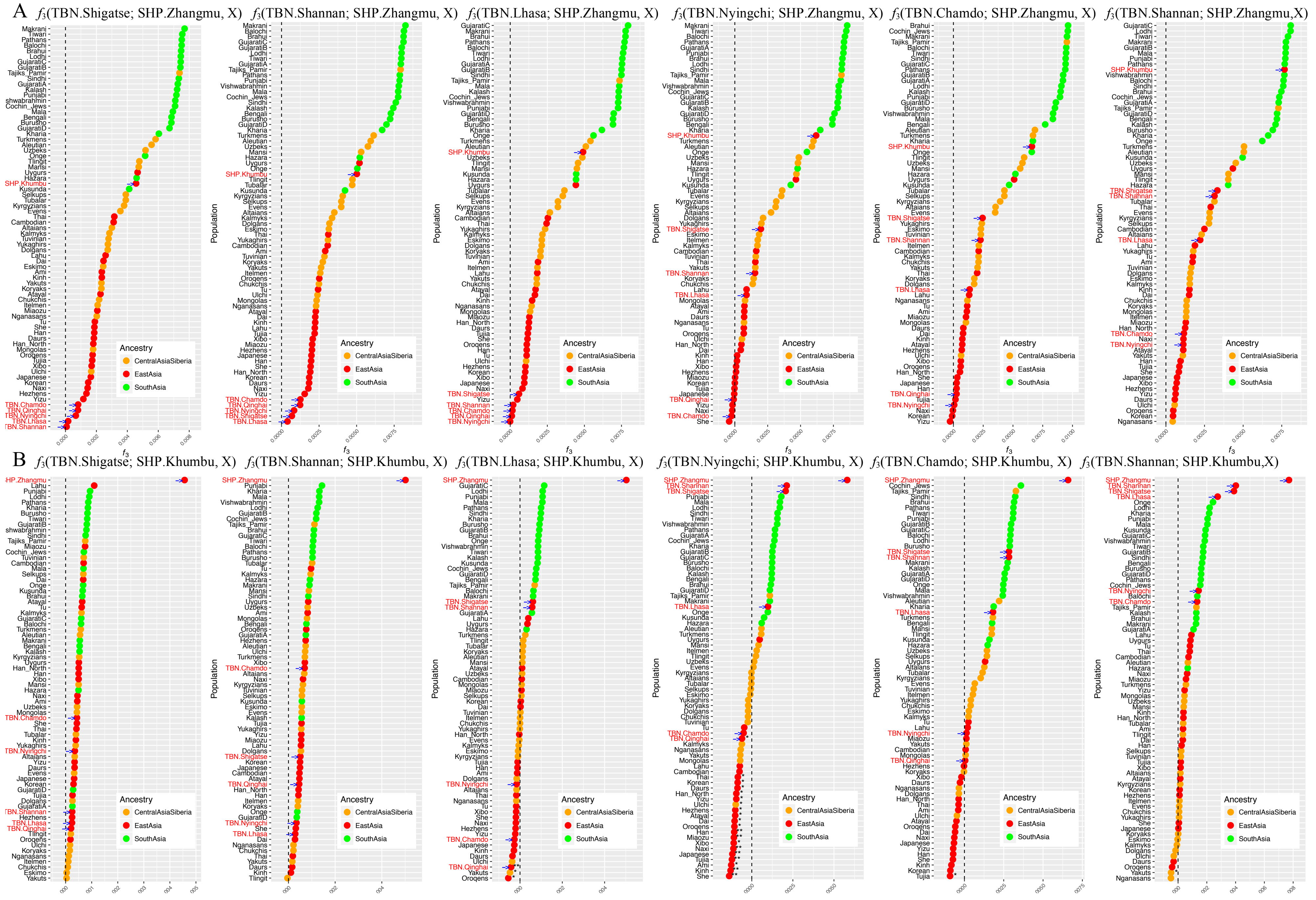

Figure S25 Detect the gene flows between SHP subgroups. (A) and (B) are panel 2 dataset-based f_3 tests in the forms of f_3(SHP.Khumbu; SHP.Zhangmu, X) and f_3(SHP.Zhangmu; SHP.Khumbu, X), respectively. Symbol '**' for Z scores ≤ -3 and '*' for -3 < Z ≤-2 in the test.

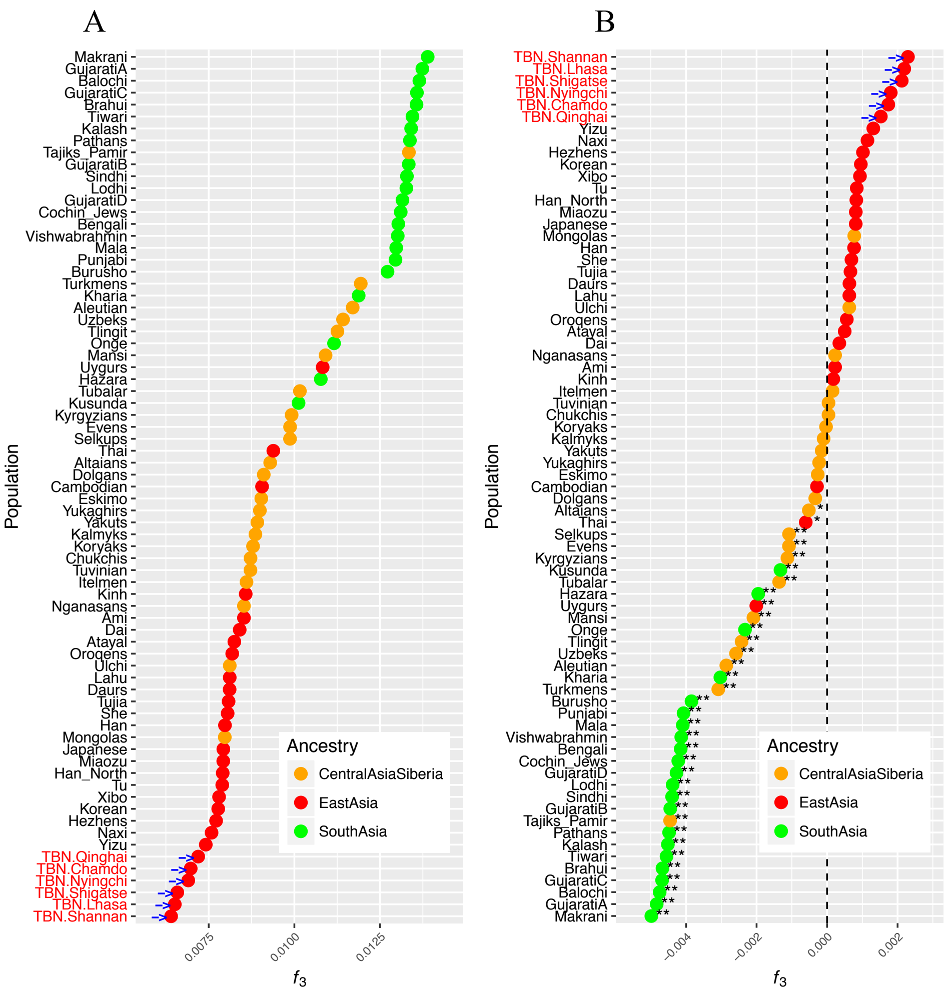

Figure S26 Panel 2 dataset-based f_3 tests of the form (A) f_3(SHP; SHPproxy, X) and (B) f_3(TBN; SHPproxy, X). SHPproxy denotes the proxy for SHP high-altitude ancestral individuals with relatively pure genetic components and less influences by gene flows and X represents East Asian, Central Asian/Siberian and South Asian populations. Symbol '**' for Z scores ≤ -3 and '*' for -3 < Z ≤-2 in the test. All the test yield significant negative scores when performing f_3(SHP; SHPproxy, X) (A). When replace the SHPproxy with TBNproxy (B), scores tends to be negative assuming X are East Asians.

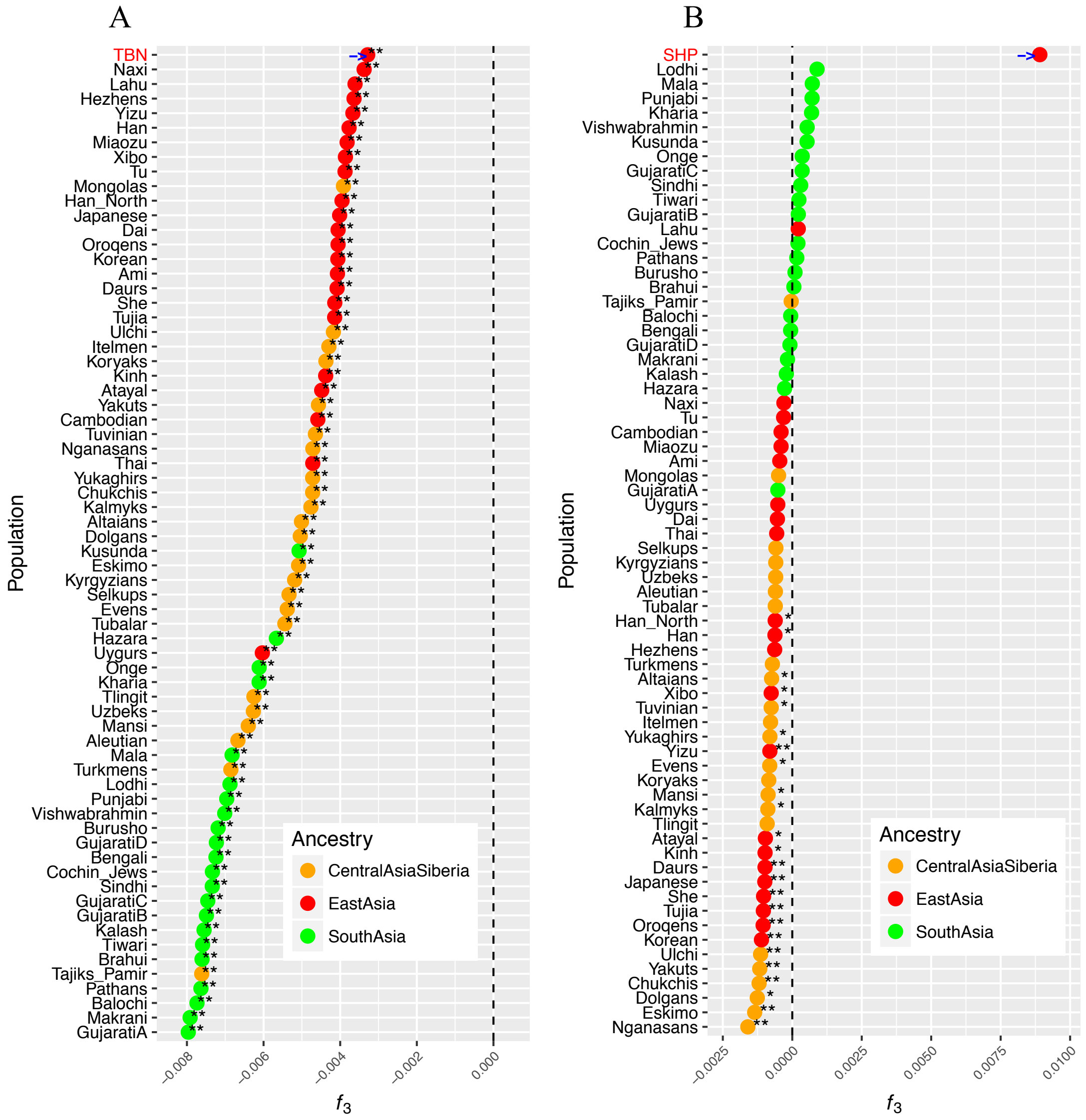

Figure S27 ***f_4* tests in the form *f_4*(SHP, TBN; Yoruba, X) to estimate the relative a mount of contribution from the reference populations to SHP and TBN.** Negative values suggest excess sharing of SHP alleles (-αg in A) and positive scores indicate X shared much more amount of alleles with TBN (αg in B). (C) and (D) show the results from Panel 1 and 2, respectively. Overall, when assuming X are South Asians, the D scores tends to be negative. While, the statistics yield significant positive values after setting East Asians and Central Asian/Siberian populations as X. Symbol '**' for |Z scores| ≤ 3 and '*' for 2 ≤ |Z| < 3 in the test.

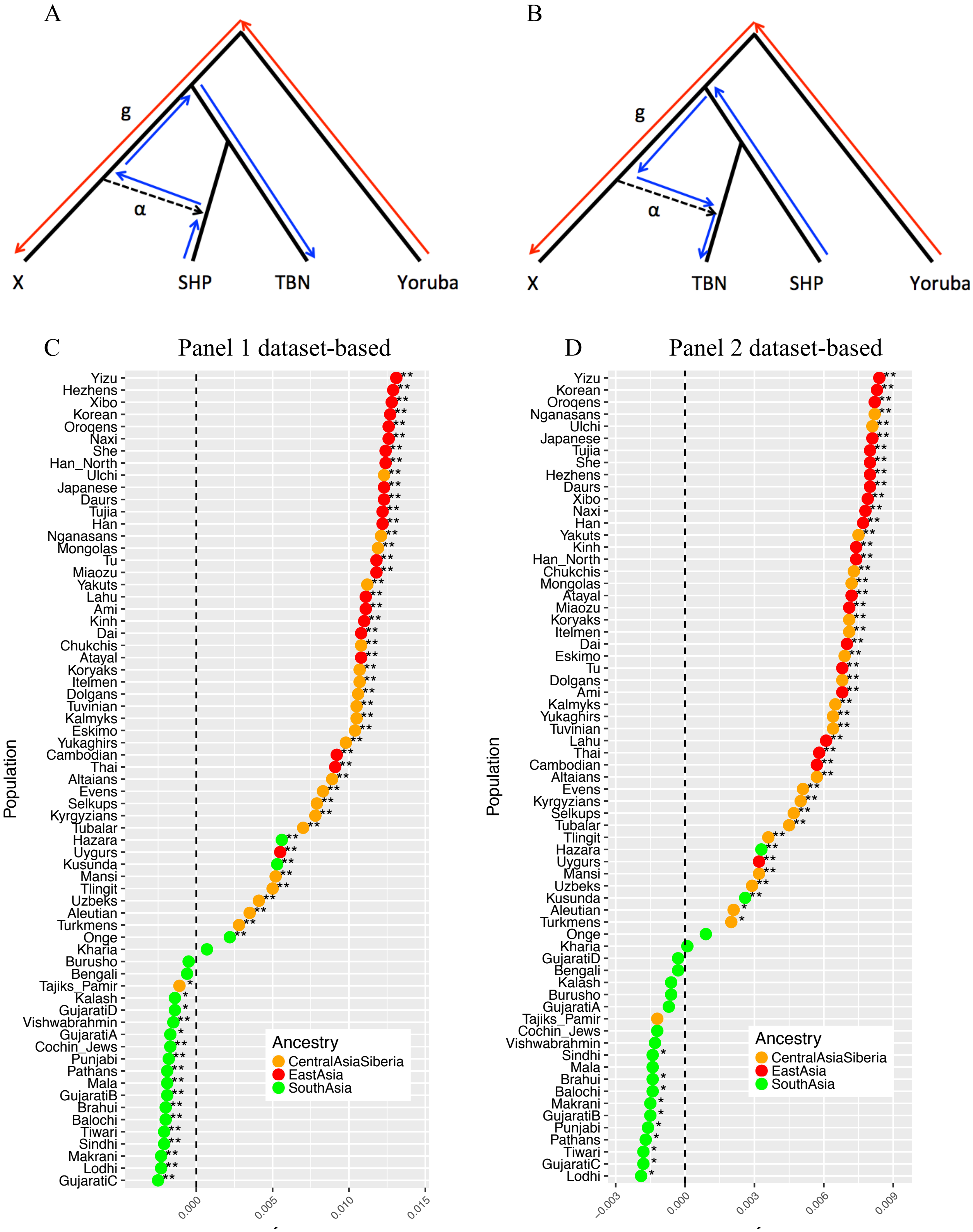

Figure S28 f_4 tests in the form f_4(SHP.Zhangmu, SHP.Khumbu; Yoruba, X) to estimate the relative a mount of contribution from the reference populations to SHPs of different geographic regions. (A) and (B) show the results from Panel 1 and 2, respectively. Symbol '**' for |Z scores| ≤ 3 and '*' for 2 ≤ |Z| < 3 in the test.

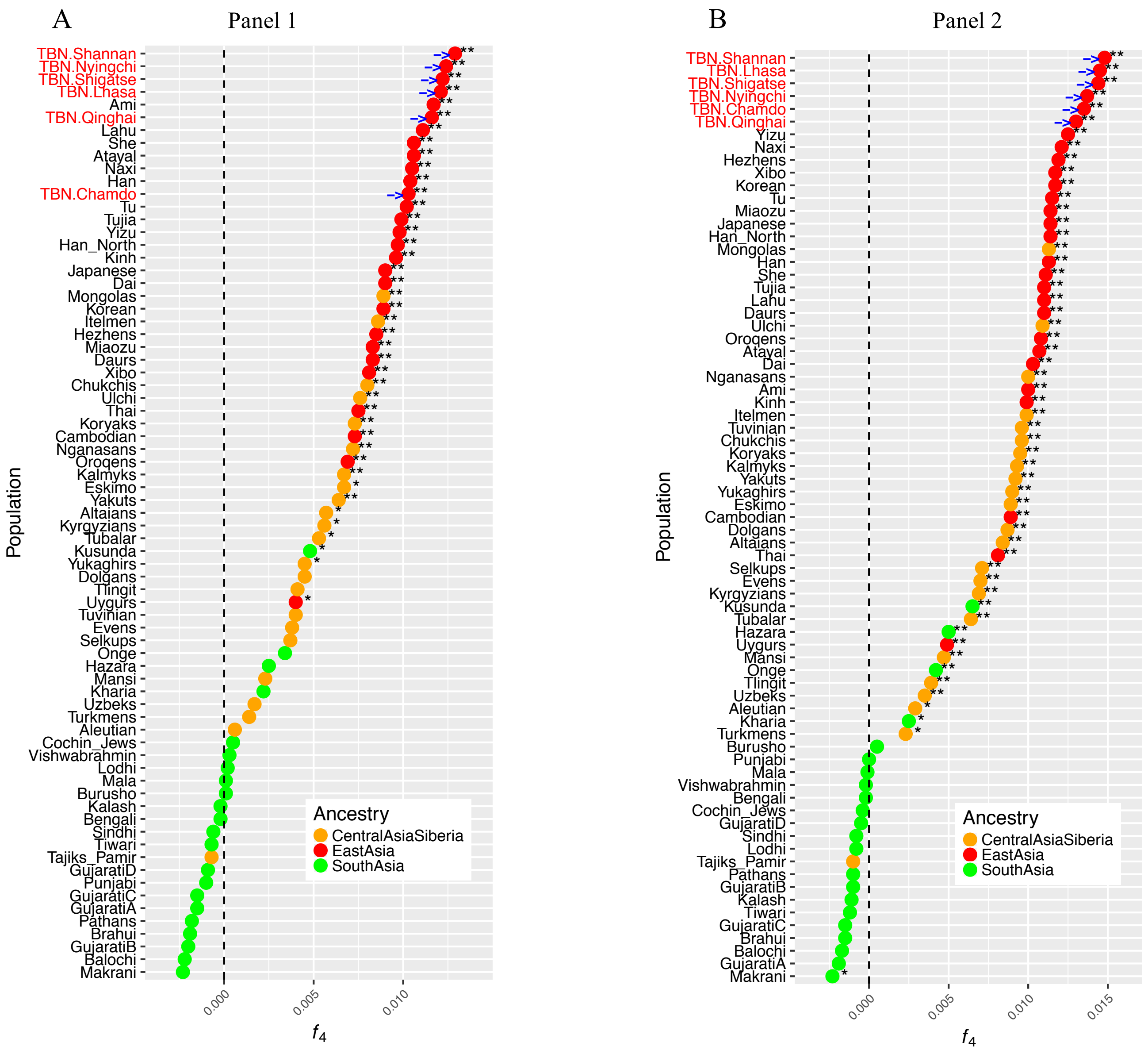

Figure S29 Panel 1 dataset-based f_4 tests in the form f_4 (TBN.Subgroup1, TBN.Subgroup2; Yoruba, X) to estimate the relative a mount of contribution from the reference populations to TBNs of different geographic regions. By performing (TBN.Subgroup1, TBN.Subgroup2; Yoruba, X), we can estimate the relative genetic affinities of other 4 subgroups to TBN.Subgroup1 (negative values if more close to TBN.Subgroup1) and TBN.Subgroup2 (positive values if more close to TBN.Subgroup1). Figures also show that TBN.Qinghai, TBN.Chamdo and TBN.Nyingchi harbor more East Asian components and TBN.Shigatse, TBN.Shannan more South Asian component.

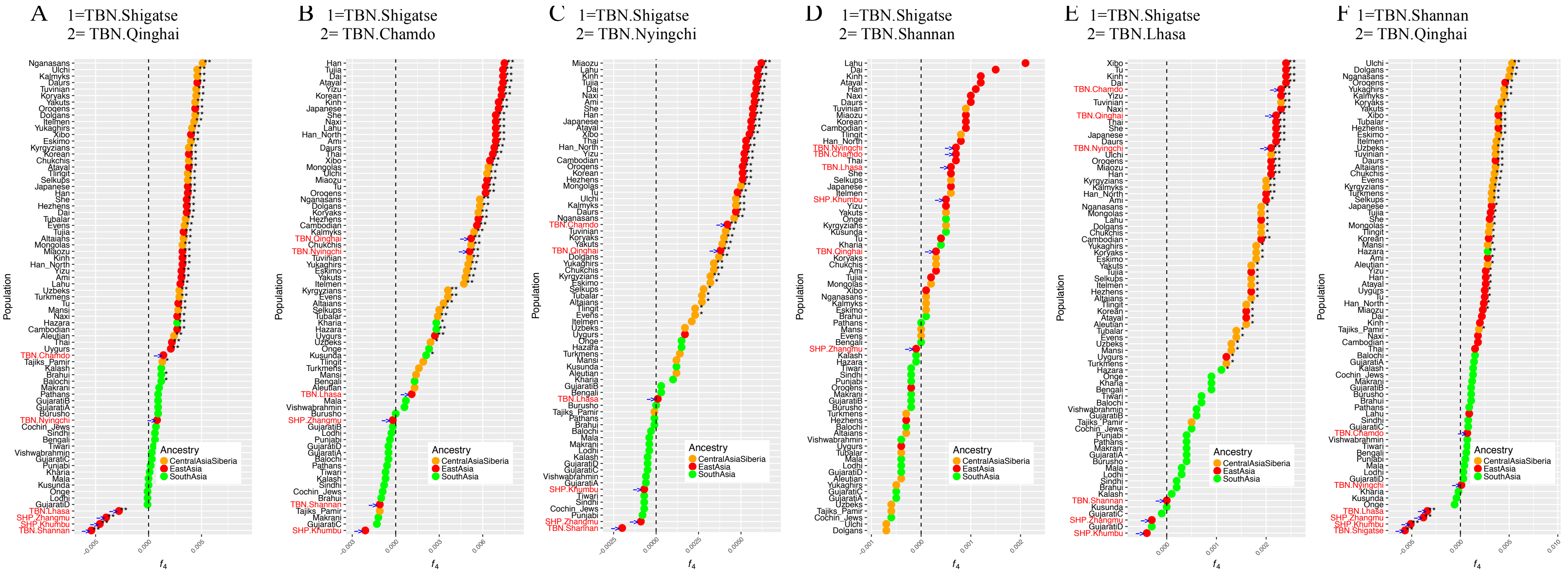

Figure S30 Panel 2 dataset-based f_4 tests in the form f_4 (TBN.Subgroup1, TBN.Subgroup2; Yoruba, X) to estimate the relative a mount of contribution from the reference populations to TBNs of different geographic regions. According to Table 1, TBN are divided into six population subgroups. By performing (TBN.Subgroup1, TBN.Subgroup2; Yoruba, X), we can estimate the relative genetic affinities of other 4 subgroups to TBN.Subgroup1 (negative values if more close to TBN.Subgroup1) and TBN.Subgroup2 (positive values if more close to TBN.Subgroup1). Figures also show that TBN.Qinghai, TBN.Chamdo and TBN.Nyingchi harbor more East Asian components and TBN.Shigatse, TBN.Shannan more South Asian component.

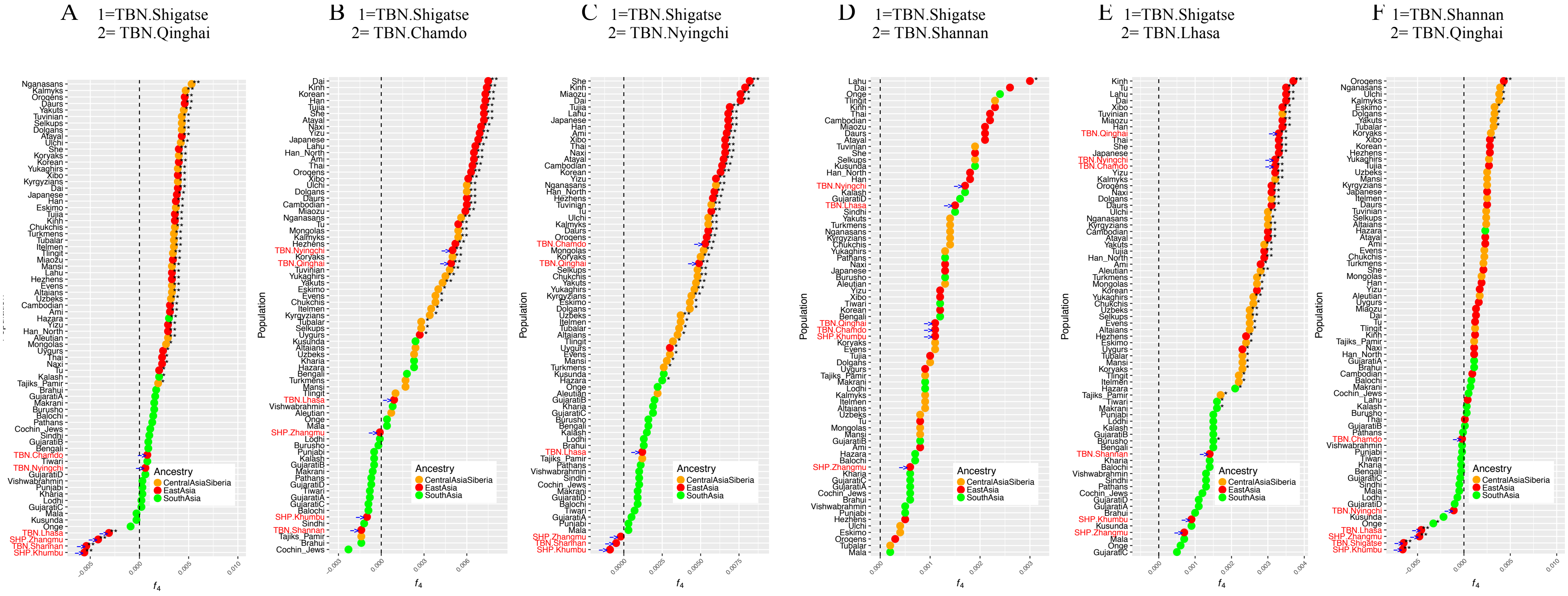

Figure S31 Admixture trees based on panel 2 with 18 populations (including SHP and TBN) were included. Plots were generated using *treemix* and shown are for migration edges m=0-10. We find migration signal from South Asian to SHP and from East Asian and Central Asian/Siberian populations to the common branch of SHP and TBN.

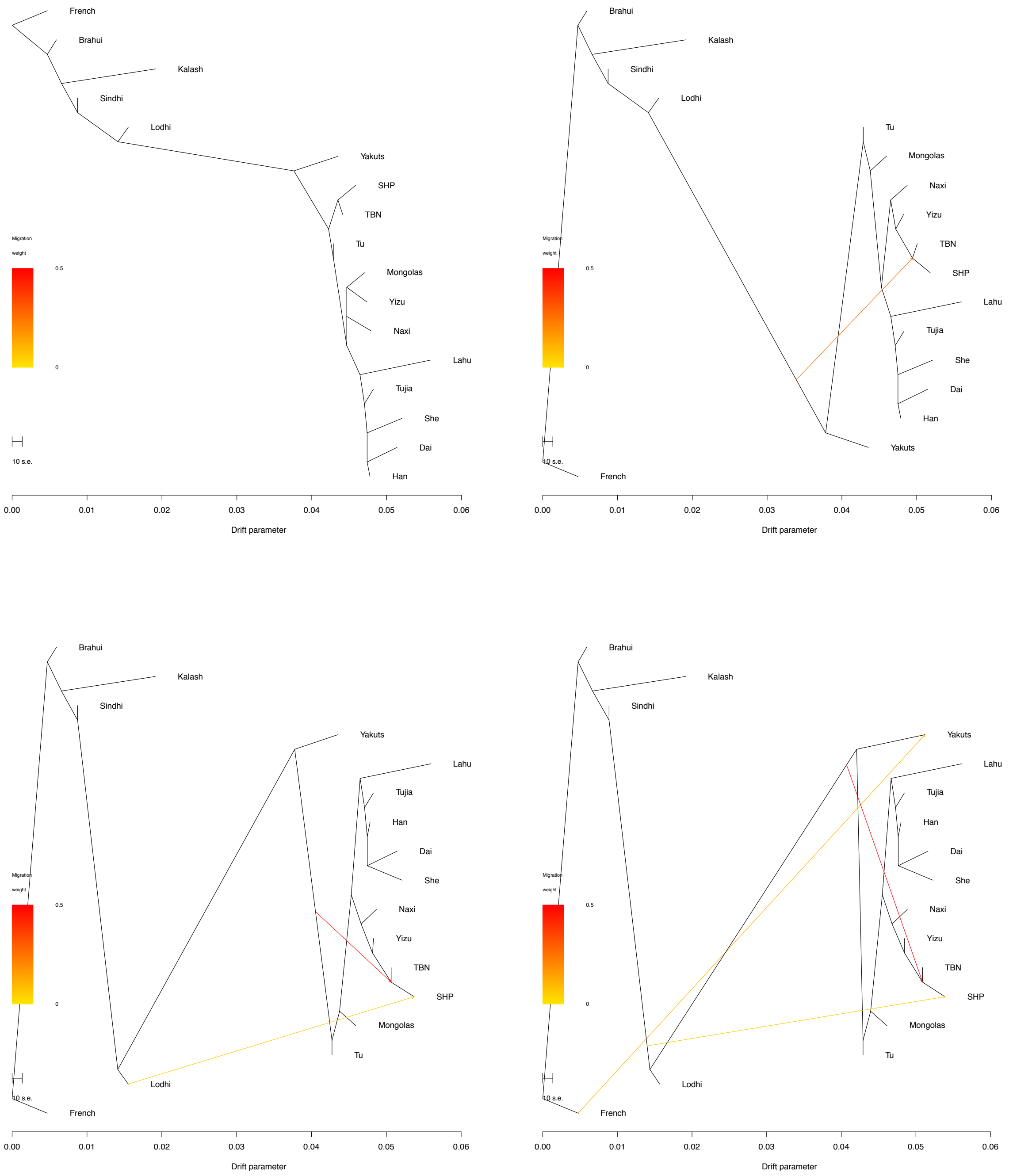

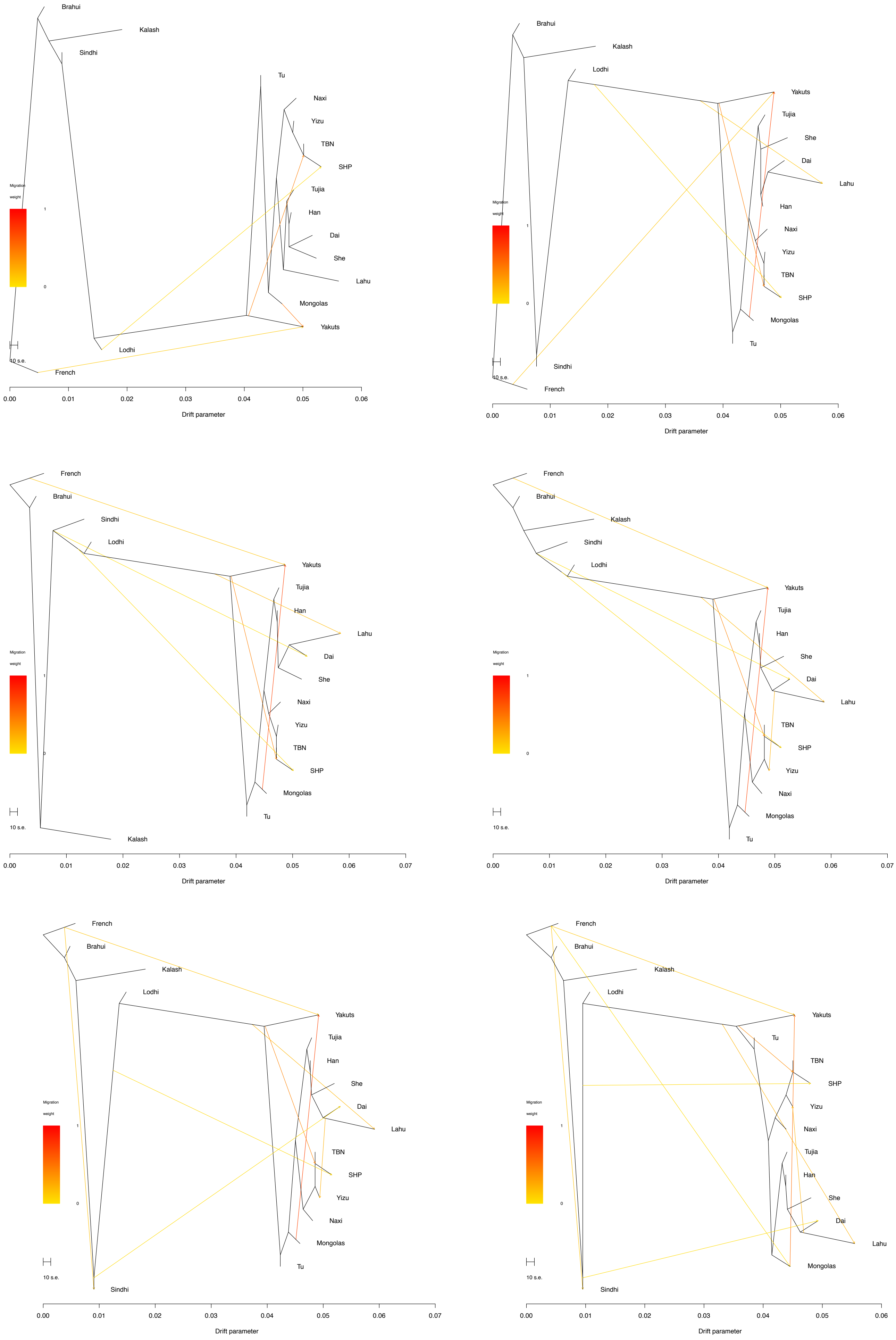

Brahui
Kalash
Sindhi
Tu
Naxi
Yizu
TBN
SHP
Tujia
Han
Dai
She
Lahu
Mongolas
Yakuts
Lodhi
French
Migration weight
1
0
10 s.e.
0.00
0.01
0.02
0.03
0.04
0.05
0.06
0.07
Drift parameter

Figure S32 Admixture trees based on panel 2 with 18 populations (including SHP and TBN) were included. Plots were generated using *treemix* and shown are for migration edges m=0-10. We find migration signal from South Asian to SHP and from East Asian and Central Asian/Siberian populations to the common branch of SHP and TBN.

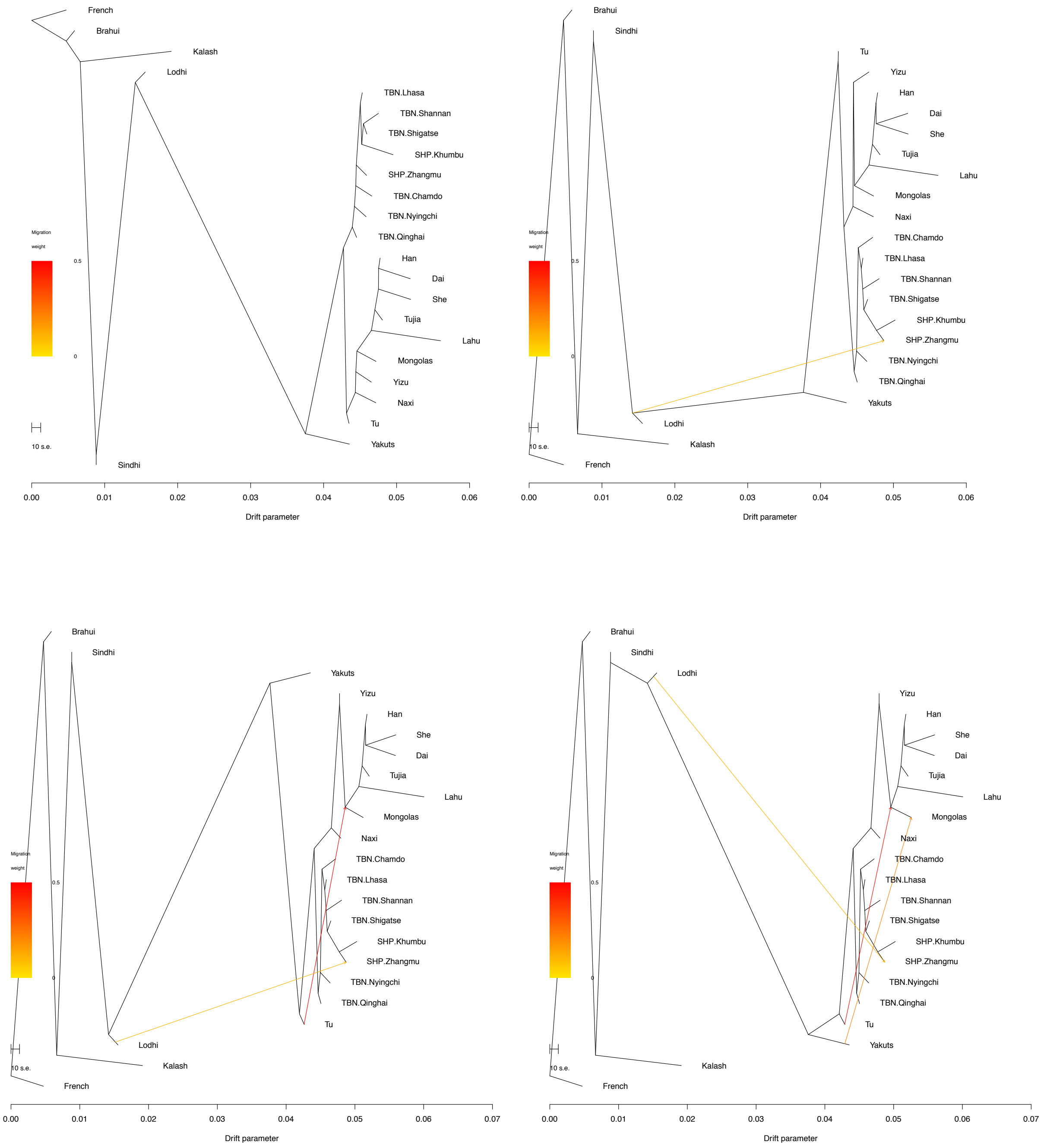

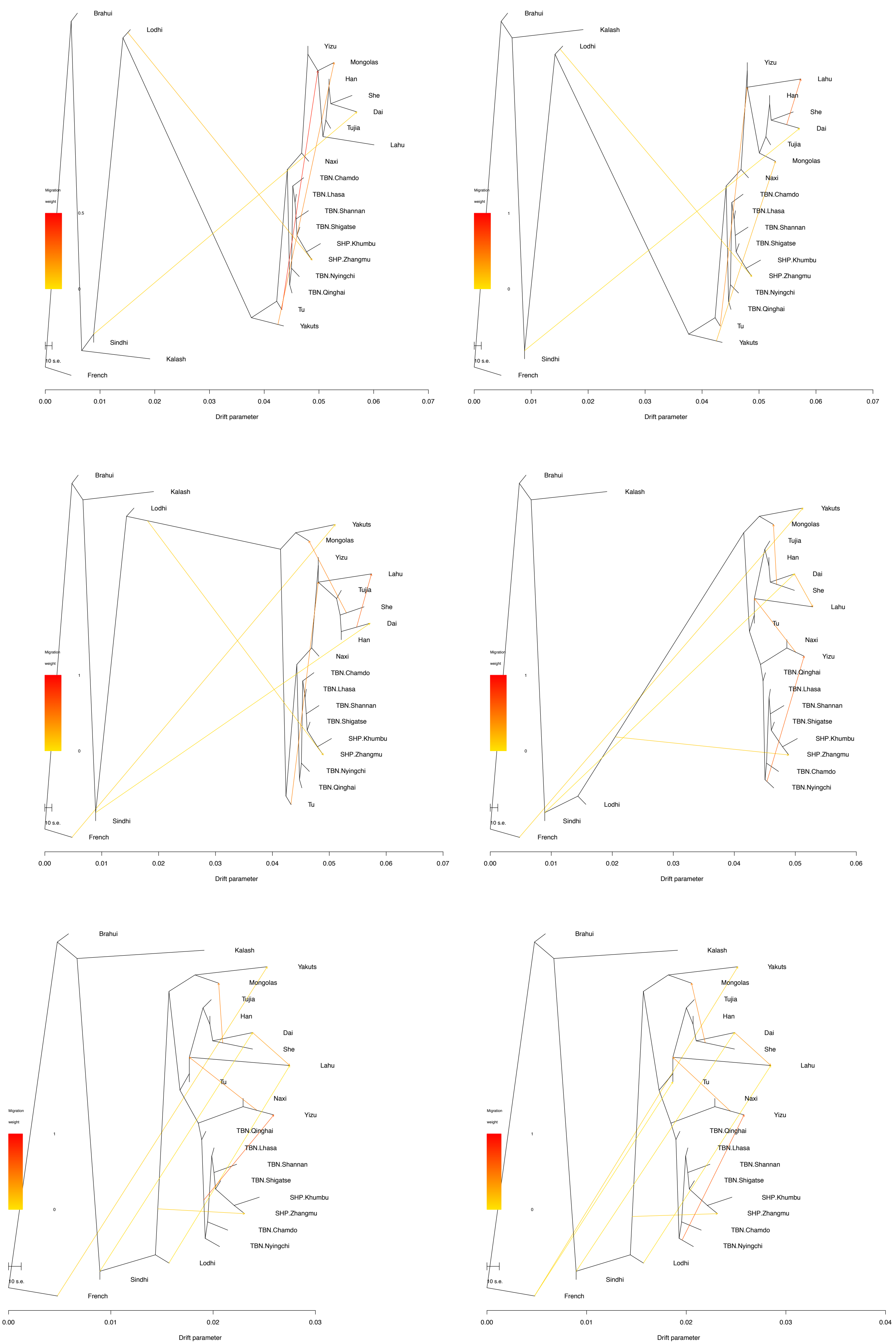

Brahui
Lodhi
Yizu
Mongolas
Han
She
Dai
Tujia
Lahu
Naxi
TBN.Chamdo
TBN.Lhasa
TBN.Shannan
TBN.Shigatse
SHP.Khumbu
SHP.Zhangmu
TBN.Nyingchi
TBN.Qinghai
Tu
Yakuts
Sindhi
Kalash
French
Migration weight
0.5
0
10 s.e.
0.00 0.01 0.02 0.03 0.04 0.05 0.06 0.07
Drift parameter
Brahui
Kalash
Lodhi
Yizu
Lahu
Han
She
Dai
Tujia
Mongolas
Naxi
TBN.Chamdo
TBN.Lhasa
TBN.Shannan
TBN.Shigatse
SHP.Khumbu
SHP.Zhangmu
TBN.Nyingchi
TBN.Qinghai
Tu
Yakuts
Sindhi
French
Migration weight
1
0
10 s.e.
0.00 0.01 0.02 0.03 0.04 0.05 0.06 0.07
Drift parameter
Brahui
Kalash
Lodhi
Yakuts
Mongolas
Yizu
Lahu
Tujia
She
Dai
Han
Naxi
TBN.Chamdo
TBN.Lhasa
TBN.Shannan
TBN.Shigatse
SHP.Khumbu
SHP.Zhangmu
TBN.Nyingchi
TBN.Qinghai
Tu
Sindhi
French
Migration weight
1
0
10 s.e.
0.00 0.01 0.02 0.03 0.04 0.05 0.06 0.07
Drift parameter
Brahui
Kalash
Yakuts
Mongolas
Tujia
Han
Dai
She
Lahu
Tu
Naxi
Yizu
TBN.Qinghai
TBN.Lhasa
TBN.Shannan
TBN.Shigatse
SHP.Khumbu
SHP.Zhangmu
TBN.Chamdo
TBN.Nyingchi
Lodhi
Sindhi
French
Migration weight
1
0
10 s.e.
0.00 0.01 0.02 0.03 0.04 0.05 0.06
Drift parameter
Brahui
Kalash
Yakuts
Mongolas
Tujia
Han
Dai
She
Lahu
Tu
Naxi
Yizu
TBN.Qinghai
TBN.Lhasa
TBN.Shannan
TBN.Shigatse
SHP.Khumbu
SHP.Zhangmu
TBN.Chamdo
TBN.Nyingchi
Lodhi
Sindhi
French
Migration weight
1
0
10 s.e.
0.00 0.01 0.02 0.03
Drift parameter
Brahui
Kalash
Yakuts
Mongolas
Tujia
Han
Dai
She
Lahu
Tu
Naxi
Yizu
TBN.Qinghai
TBN.Lhasa
TBN.Shannan
TBN.Shigatse
SHP.Khumbu
SHP.Zhangmu
TBN.Chamdo
TBN.Nyingchi
Lodhi
Sindhi
French
Migration weight
1
0
10 s.e.
0.00 0.01 0.02 0.03 0.04
Drift parameter

Figure S33 Estimates of historical effective population size (N_e) based on LD method. CHD here represents Chinese Dai in hapmap dataset.

A

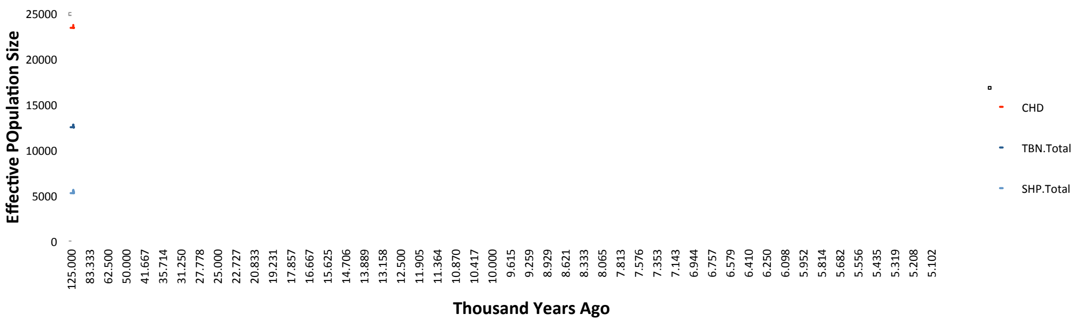

B

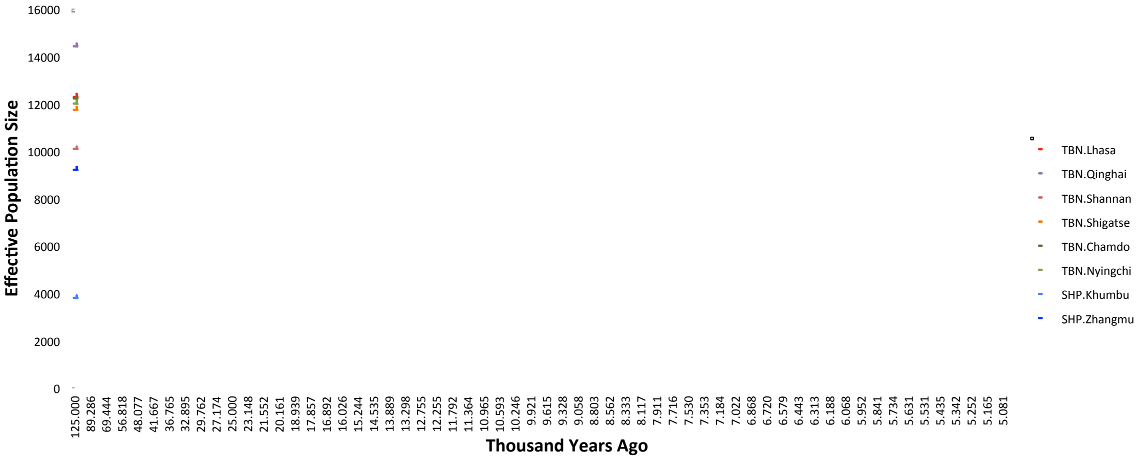

Figure S34 Divergence times estimated by MSMC. Estimates of divergence time between (A) TBN and others; (B) HAN and others; and (C) IDN and others using MSMC. Divergence time between each pair of populations was evaluated using autosomal sequences of 4 genomes, i.e., 2 individuals for each population. An autosomal mutation rate (μ_{Auto}) with 1.25×10^{-8} per base-pair per generation and 25 years per generations (g) were used.

A

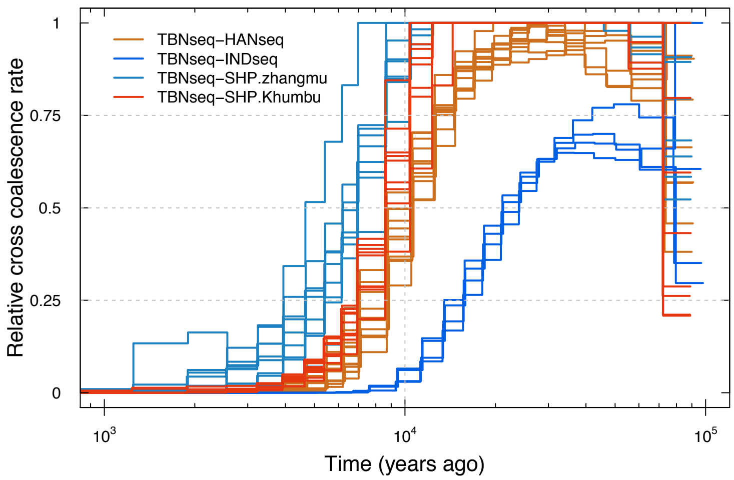

B

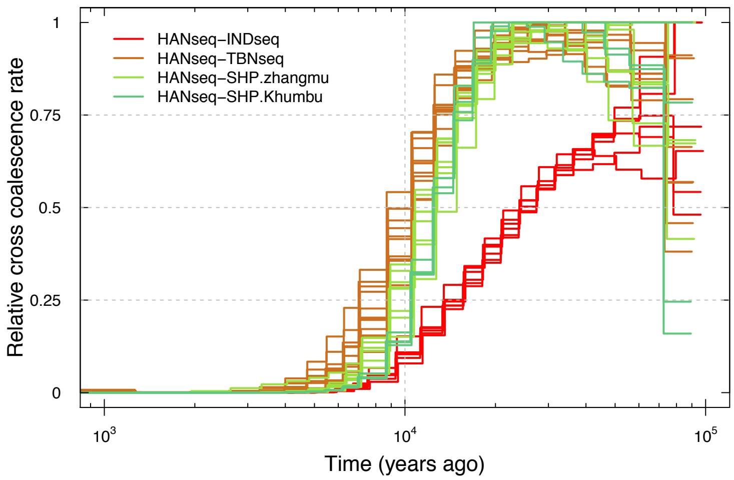

C

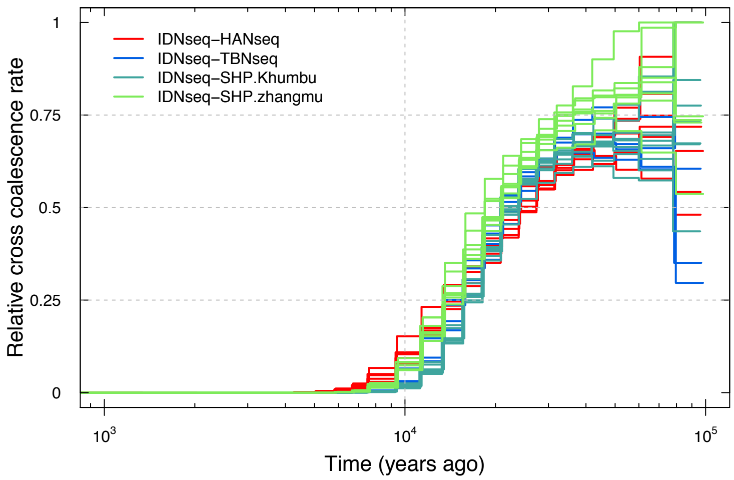

Figure S35. PBS and XP-EHH results for *ALDH3A1* region. (A) PBS and (B) XP-EHH.

A

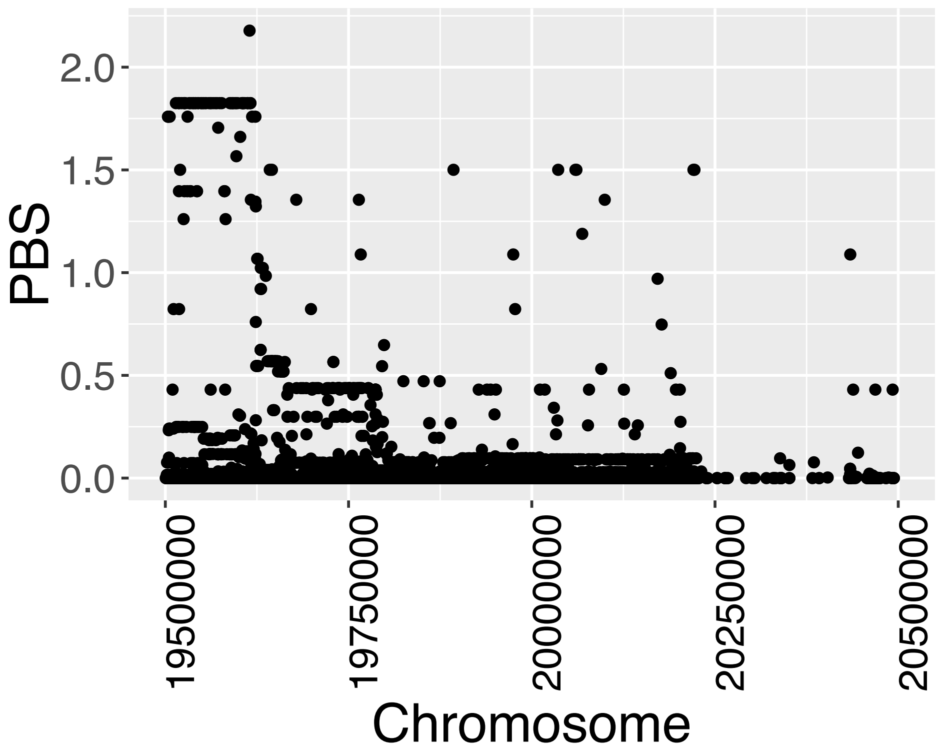

B

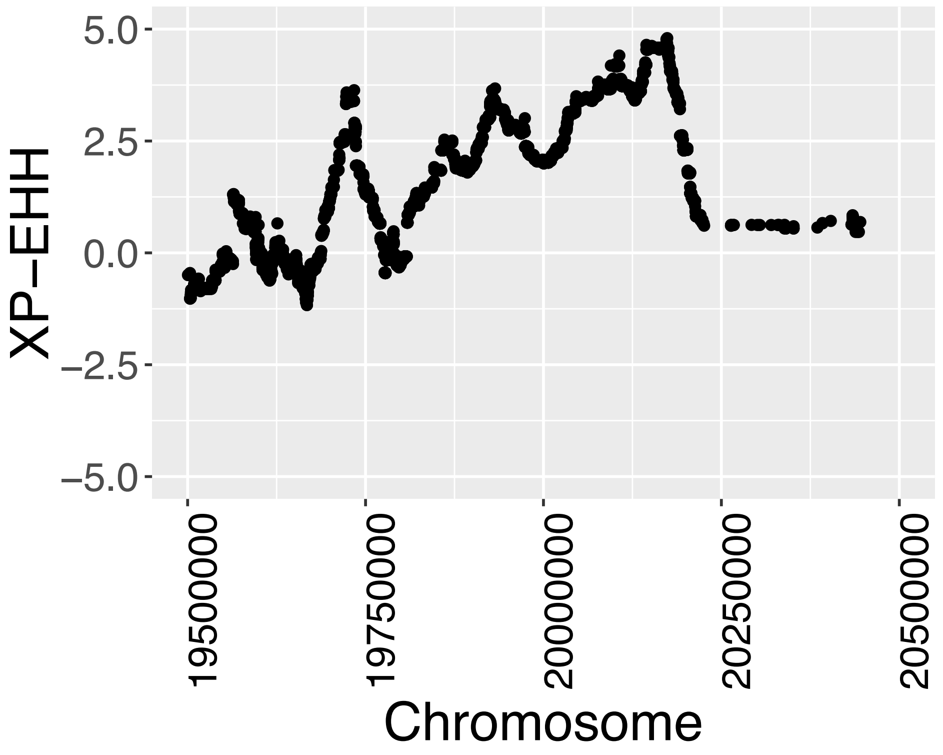

Supplemental Tables

Table S1 PCR and SNaPshot primers used in this study.

Site		PCR Primer (5'-3')	Extension Primer (5'-3')
rs17452596	F	CCCTCCTGCCTCCTTGATGTTC	GTCTCTGGGTTCCGGAGGC
	R	GGGGGATGCAGAAGACCACCTA	
rs1049230	F	CCTACCTCCGAGTCACGCTCAA	GAAATAAAGTGCMTAGGTCCTGGG
	R	CCGGTGTGACAGGTAGGACTGG	
rs10813831	F	GGTCCAGGGTCTTCCGGATATAA	TTTTTTTTCATGACCACCGAGCAGCGA
	R	ACAGCCTGCGGGGAACGTA	
rs7205130	F	TTTCAGCAATAGGTCAGAGGTGTCC	TTTTTTTTTTTTACAGCGCTTGCCTTTGCAG
	R	CCTGACCTGCGTCCAGAGCTT	
rs1049229	F	CCTACCTCCGAGTCACGCTCAA	TTTTTTTTTTTTTTTAGTGCMTAGGTCCTGGGRC
	R	CCGGTGTGACAGGTAGGACTGG	
rs56130225	F	GCCGCAGGGACAGAGTATACGA	TTTTTTTTTTTTTTTTTCACACACCCAGCAAGGCTAC
	R	CCTGCATGTGTTCACACGGACT	
rs3764990	F	CACGAAGTGGAGGCTTCCTGTG	TTTTTTTTTTTTTTTTCACTGTGAAGGAGATGATGAGAGC
	R	GGGGGATTAACCTGCCACTGAA	
rs200891942	F	TGGCTCTCATCCCGAGAGTCAT	TTTTTTTTTTTTTTTTTTTTTTGAAAGGCTACTCGAAGACCA
	R	GGCTACCCCTAGAAGAAGCAGGTC	
rs12769244	F	TGGGCCCATTCTGAACCAAAG	TTTTTTTTTTTTTTTTTTTTTTTTCGTGCAGTCTGCTGTTGTCT
	R	CAGTTCCAGTGCAGTGTTTTCTGAG	
rs671871	F	TCTTCAAAGGCTTCAACCGATCA	TTTTTTTTTTTTTTTTTTTTTTTTTTCAAACAAAAGGTTGGCTTGA
	R	TGTTTTTCCCTCTCCCCAACTTTC	
rs13100173	F	CCCAGTTCCCAGCCCAGAGT	TTTTTTTTTTTTTTTTTTTTTTTTTTTTCACTGGCTGCCTACCAGATC
	R	AACCGTGGCCAGCATTTTCAC	
rs190140224	F	GCTATTACCTTGTGTTGTCACCCAAGTCT	TTTTTTTTTTTTTTTTTTTTTTTTTTCAGTTCTGAGTGTAACAGGTTTGA
	R	AGTCAAATTTTAGGAAATCAACTGTCTACTTTTTT	
rs61761321	F	GCAGTATCCATGACCCCTGTTCC	TTTTTTTTTTTTTTTTTTTTTTTTTTTTTTCACACTCAGCGTCATTCTCCA
	R	ACCAGATATGCAGGCGTCACAA	
rs61740375	F	TGCAATCAATAGTGCTGAAGCTGAA	TTTTTTTTTTTTTTTTTTTTTTTTTTTTTGAAAGGGGTTTCAATTTAATGAAC
	R	CCAACCTGTGGTTGCAATTTGA	
rs35830695	F	TCATTCCCACTCAGACCCCTACC	TTTTTTTTTTTTTTTTTTTTTTTTTTTTTTTCTTTGATGAACTGGACTCTTTGG
	R	CACCTAACTTCAACCCCCCACAA	
rs28921397	F	GAGCATCCCAGGGAGTAAGCTGT	TTTTTTTTTTTTTTTTTTTTTTTTTTTTTTTTTTCAGAAACCTAAAGGGACTATTG
	R	AAGAAAAATAGCTTTTTCGAAAGCAAATAAC	
rs857705	F	CCATCCTGAAGCTGAGGGAATC	TTTTTTTTTTTTTTTTTTTTTTTTTTTTTTTTAATTCAGTCACTGTTGATTGGTTTC
	R	CCCTCTCATGTGCTGGACTATGC	
rs149597385	F	TTGGAAGGCATCCACAGATTCTAA	TTTTTTTTTTTTTTTTTTTTTTTTTTTTTTTGGATGTAAGCAAGATAACTCGTTATACC
	R	AAGCGAAAAATGGATGTAAGCAAGA	
rs12489516	F	TGGTCAACAAGGGATCAATGTGTG	TTTTTTTTTTTTTTTTTTTTTTTTTTTTTTTAAAGAATGAAAGAAGAAAGGCTATTTTTA
	R	CCTGATAGACAAACCACTGGCAGAA	
rs1326331	F	GGTCTGTTGTGAATCTAACCAACCAAAAT	TTTTTTTTTTTTTTTTTTTTTTTTTTTTTTTTTCCACCTGTAAAGTATCTGCAAATATTACA

	R	TGTTAGGTTATCACCCACCTGTAAAGTATCT	
rs79496824	F	GCCGCAGGGACAGAGTATACGA	TTTTTTTTTTTTTTTTTTTTTTTTTTTTTTTTTTTTTTTTTTCCTGGTGCAAGAACACATACA
	R	CCTGCATGTGTTCACACGGACT	
rs7189101	F	TTTCAGCAATAGGTCAGAGGTGTCC	TTTTTTTTTTTTTTTTTTTTTTTTTTTTTTTTTTTTTTTTTTTTTCTCCTCACTCAGTCTGCTGC
	R	CCTGACCTGCGTCCAGAGCTT	
rs1049232	F	CCTACCTCCGAGTCACGCTCAA	TTTTTTTTTTTTTTTTTTTTTTTTTTTTTTTTTTTTTTTTTTTTTTTACGGGGTCAGAAATAAA GTGC
	R	CCGGTGTGACAGGTAGGACTGG	
rs117428385	F	TGAGTTTGACGCCTACACCTCTCA	GACCCCACGCCGCAGCCCC
	R	CCATCCCCCAGGGAACCAT	
rs11466018	F	CTCGGGGTGGTCTGGAGTCTTC	TTTTTTCCGCAGCGTCCAGCTCCC
	R	CCCTAAACGTGGGACAGCTTCA	
rs12604031	F	AGTGCGTGGCTGGGTGAGTC	TTTTTTTTGCGTTTCGCAATCAGCCGC
	R	CCTCCCAAAACACCTGGTTGAG	
rs2290505	F	CCCTCACTCCCTTCCCCTTGTT	TTTTTTTTTTTTCCCAGGGCTGCTATGTCGC
	R	CTCAGTGCCCAGGGCTGCTAT	
rs1040461	F	CCATGACAGCTGGATGGGTTTC	TTTTTTTTTTTTAAGTCACTCCGGTCAGAATTCA
	R	GGTGGAAGTCACTCCGGTCAGA	
rs3741395	F	CTCCGCCAGGGCAAAGAGAC	TTTTTTTTTTTTTTTTACTGGGGCTCAAGGTCAGC
	R	CGCAGCAACGGTACCTATCAAA	
rs146810291	F	TTGACTGCCCGTGGTCCATCTA	TTTTTTTTTTTTTTTTTTTTTCATCGCACTGACCTTCAGC
	R	GTCACCACGAAGGCCAGTTTTG	
rs983034	F	GGGATGGTGCATACAAGAACATCA	TTTTTTTTTTTTTTTTTTTTGCACTGTTCACTTGGACTGTGA
	R	CACCATCCTTTCTCCGCAGGTA	
rs1801692	F	CGATTTGCATTTGAAAGTCCAACCT	TTTTTTTTTTTTTTTTTTTTTTTTTGAATATCCCAACACGATCA
	R	TGGAGCCGTACGCTATACGACTTT	
rs9294445	F	TCCCAAGGCCTCGTAGAACCTC	TTTTTTTTTTTTTTTTTTTTTTTTTTCTGAGCTCCACACCTCACTC
	R	TGCAGGCATCCATATTGCAGTT	
rs201075024	F	GCAGTGAGGACCCAGGTGTCTG	TTTTTTTTTTTTTTTTTTTTTTTTTTTTTCTGTGGACRGCGTGGCCCC
	R	CTTGGGTCTGGGTTGGGACTG	
rs74564278	F	CCGGGAATATCAGCTGCCTTCT	TTTTTTTTTTTTTTTTTTTTTTTTTTTCGAAGGAGCCTCTAATTCACTTT
	R	GGGACTTGCGAAGGAGCCTCT	
rs12449313	F	AGAGCGATGGTCAGTTGGTGCT	TTTTTTTTTTTTTTTTTTTTTTTTTTTTTGACTTTTCAGTGGAAAATGCCA
	R	GTGGCTCGGGTCCAGCTCATAA	
rs138364911	F	TGCCTTGTGATTGCAGGAACAT	TTTTTTTTTTTTTTTTTTTTTTTTTTTTTTTCGACGTATGTCTCCCATTCTTA
	R	GACCCTGCTGGGATGCTTGAG	
rs3752105	F	GGAGAAGCTGTCGGCCCTAGAA	TTTTTTTTTTTTTTTTTTTTTTTTTTTTTTTTTTCAAACCTCGCGAGGAGAAGA
	R	CTCCCTCCCCTCCCTCCACT	
rs117922207	F	GCTACCCCCTCCAGGGACAGT	TTTTTTTTTTTTTTTTTTTTTTTTTTTTTTTTTTTCCACGGCAAAGTACACRGGGA
	R	CGTCCTCCACGGCAAAGTACAC	
rs549340789	F	GGCTCAGCACAGGCTCTATCTCC	TTTTTTTTTTTTTTTTTTTTTTTTTTTTTTTTTTTTTTTCTGGTTGCTTTCTTCRCG
	R	GAATGGGCCTCAGCATGCCTAC	
rs938952	F	GGGGCTGTTGAGCAACTCTAGGA	TTTTTTTTTTTTTTTTTTTTTTTTTTTTTTTTTTTTTTTTAGAGAGGCCACCACTCCAC
	R	CCGATGGCTCCTCCAGAATCAT	
chr3:196920905	F	CAAATCCATGGACATTCTCAATCTCTG	TTTTTTTTTTTTTTTTTTTTTTTTTTTTTTTTTTTTTCTGGACCTATCACTTCATTTGTG
	R	CATGTTGCTTATCAGGGCTTCTTCA	

chr8:108263611	F	CTGGCAGCTTCTCCGGATTTCT	TTTTTTTTTTTTTTTTTTTTTTTTTTTTTTTTTTTTTTCCAGTTACTCCTTACGTTCCACAA
	R	GCTTGTGGCCCCTCCAATCTAA	
chr17:19644917	F	AGGGACTCTTCCCTCCCAGCTC	TTTTTTTTTTTTTTTTTTTTTTTTTTTTTTTTTTTTTTTTTTTTTTGGGGTGGGGAAGATCATC
	R	GCCACTGCAACTCACCCACACT	
chr16:2146963	F	CTTCAGAGCCCCCTCCTCTCAC	TTTTTTTTTTTTTTTTTTTTTTTTTTTTTTTTTTTTTTTTTTTTTTTTTGAGGGAACGCTCAGTTGGC
	R	GGCGAGGGAACGCTCAGTT	
rs150145464	F	CGGGACGAGCTCGGATTGA	CCAGCCTGGCSGACTAGGA
	R	ACCACGATGACCCCAATTTCAC	
rs116035113	F	ACACGGCGACCAATGTTTCAA	TTGGTAGCAGGCTCCCATTTTTG
	R	GTGGGCTGCTAGCTCAGGCTGT	
rs9353689	F	AACTGATCAAGGCCCTCCACAA	TTTTTTTTTCCAGAGTGGTGTCAGGGC
	R	ACAGGCCACAATTCCAGAGTGG	
rs78422682	F	GAGGTGGAGGGGAACACAGGTC	TTTTTTTTTTTTAACTGATTCCCAAGCCTGC
	R	ACCCCTGTGCCTGGCTCTCTAC	
rs2992753	F	CTGCCTCCGATGTTGACCTGAC	TTTTTTTTTTTTTCCTCCGCCTTCTGTATGAAAT
	R	AACCCCCTCCGCCTTCTGTATG	
rs2992752	F	GCCAAGCCGGTGACACAAAG	TTTTTTTTTTTTTTTTTTAAGAGCCGCCTCCCCGCAG
	R	CCCGGCTGCTTCTGCCTAA	
rs2302657	F	TGGACTCGAGCATAAGGGGAGT	TTTTTTTTTTTTTTCAGGTGTGAGTATACAGTTTCTTTGT
	R	TGTTTTTATTTTGTGCATTTCAGGTGTG	
rs200883298	F	CTACCTGGGCGACCGACATAGC	TTTTTTTTTTTTTTTTTTTTCCTCTCTAGCTCCTTCATCTCCC
	R	CACAGCCAGGAATGGAGCAGAA	
rs73362428	F	CAAGTGACTTTTTCAGTTCCTTCTGTTCC	TTTTTTTTTTTTTTTTTTTTTTCTGTTCCTTAAGTGTGTTTTCCA
	R	GCTCATCATAATTTAGTTTATTTCTTCGTGTG	
rs35496730	F	CACCTTGCCCCAAGCTGCTG	TTTTTTTTTTTTTTTTTTTTTTTTTTTTAATGGAATTCCTCCCCAGTG
	R	GGGTGACTCGTCCCACTTGTGT	
rs2992755	F	CAGACAGGAAGCCCCAGAGAAAAG	TTTTTTTTTTTTTTTTTTTTTTTTTTTTTTTCTGTTCTGGTTTCCTGGCTG
	R	TGAGTCCAGCTGGGGAGCTCTT	
rs3748085	F	GTGCCTTTCCCACCTGTGTGTC	TTTTTTTTTTTTTTTTTTTTTTTTTTTTTTTTTCACGTTGGCTCCTGCTTTAA
	R	CACTGGAAGAAGGCTCCCACAG	
rs80009430	F	TGTTACCTCCCATGCCTGCTTTC	TTTTTTTTTTTTTTTTTTTTTTTTTTTTTTTGGAGAAATTTGAACTCAAGCCAAT
	R	TCGGATTTTGGCTTGTTGTGGT	
rs7498	F	TGCTTGAGGCTACTGGGGACTG	TTTTTTTTTTTTTTTTTTTTTTTTTTTTTTTTTTTTTTCCTTTACCCGCTGCTTCCA
	R	GCAAGGGCAATGAGCAGCAC	
rs55930890	F	CGAAAGCGACCTCTGCAAAGAC	TTTTTTTTTTTTTTTTTTTTTTTTTTTTTTTTTTTTTTTTTGGGTGTGAGGGTTGGGGG
	R	GAAACTCCTCTCCGGCTTCACC	
chr17:635695	F	AAGCCCCTCCGCGTCCTC	TTTTTTTTTTTTTTTTTTTTTTTTTTTTTTTTTTTTTTTTTTGCTCATCGGCCATTCCCGC
	R	GCAGGAGCGAATGATGACGAT	
rs80006813	F	CAATATCCGAAACCAAAGACGATGA	TTTTTTTTTTTTTTTTTTTTTTTTTTTTTTTTTTTCTACTATTATTCTTTCCAGCACAATCCT
	R	CCCTTGAAGCTTCCTTAAGTTTGTTCTC	
rs34740210	F	CAGTGAGCCCCTTCAGCAACTT	TTTTTTTTTTTTTTTTTTTTTTTTTTTTTTTTTTTTTTTTGCATCCAGAGTTGCTGTATATCTTC
	R	ACAGTGCGCTCACCACACAGAT	
rs142922734	F	ATTGCCAGCTGAGTCCGTGTG	TTTTTTTTTTTTTTTTTTTTTTTTTTTTTTTTTTTTTTTTTTTTTTTTAGAGCATGGAGCAATTCACC
	R	CCTCCTTCACCTGGCTGGAAGT	

Table S2 Analysis of Molecular Variance (AMOVA) results. When two SHP subgroups and six TBN subgroups are classified into SHP and TBN groups, the source of variation among groups is significant larger than that within groups. Assuming any TBN subgroup (except TBN.Shannan in Panel 1) as a SHP group yields larger source of variation within groups than among groups, indicating that SHP and TBN are genetically two distinguishable populations. Both the small sample size of SHP.Khumbu (2) and TBN.Shannan (9) in Panel 1 may bias the results. Both results from Panel 1 and Panel 2 are shown. ** $P \leq 0.001$; * $0.001 < P \leq 0.01$.

Panel 1

Source of variation	Variance (%)						
	SHP VS TBN	SHP, TBN.Shigatse VS Other TBN	SHP, TBN.Lhasa VS Other TBN	SHP, TBN.Shannan VS Other TBN	SHP, TBN.Nyingzhi VS Other TBN	SHP, TBN.Chamdo VS Other TBN	SHP, TBN.Qinghai VS Other TBN
Among groups	0.50**	0.17**	0.11**	0.35**	0.31**	0.31**	0.05**
Among population/subgroups within groups	0.24**	0.39**	0.43**	0.31**	0.32**	0.33**	0.46**
Within population	99.26	99.44	99.46	99.35	99.36	99.36	99.48

Panel 2

Source of variation	Variance (%)						
	SHP VS TBN	SHP, TBN.Shigatse VS Other TBN	SHP, TBN.Lhasa VS Other TBN	SHP, TBN.Shannan VS Other TBN	SHP, TBN.Nyingzhi VS Other TBN	SHP, TBN.Chamdo VS Other TBN	SHP, TBN.Qinghai VS Other TBN
Among groups	0.53**	0.28**	0.23**	0.43**	0.40**	0.40**	0.12**
Among population/subgroups within groups	0.42**	0.55**	0.58**	0.47**	0.47**	0.49**	0.64**
Within population	99.04s	99.17	99.20	99.08	99.13	99.08	99.24

Table S3 Information of selected proxies for SHP (SHPproxy) according to *ADMIXTURE* when assuming K = 6 (see also Figure 3) with each individual's major genetic component > 97%. The selected proxies are viewed to be pure and less influenced by gene flow.

ID	Subgroup	% (Major component)	Symbol
CB1031	SHP.Khumbu	0.99995	SHPproxy
CB1017	SHP.Khumbu	0.99995	SHPproxy
CB1013	SHP.Khumbu	0.999949	SHPproxy
CB1046	SHP.Khumbu	0.99995	SHPproxy
CB1018	SHP.Khumbu	0.99995	SHPproxy
CB1012	SHP.Khumbu	0.99995	SHPproxy
CB1005	SHP.Khumbu	0.99995	SHPproxy
CB1026	SHP.Khumbu	0.99995	SHPproxy
CB1055	SHP.Khumbu	0.99995	SHPproxy
CB1010	SHP.Khumbu	0.99995	SHPproxy
CB1032	SHP.Khumbu	0.99995	SHPproxy
CB1003	SHP.Khumbu	0.989202	SHPproxy
CB1036	SHP.Khumbu	0.983285	SHPproxy
CB1075	SHP.Khumbu	0.99995	SHPproxy
CB1050	SHP.Khumbu	0.980921	SHPproxy
CB1040	SHP.Khumbu	0.999716	SHPproxy

Table S4 Divergence time estimated based on F_{ST} and N_e (T_F). The analysis is based on chip array dataset with the T_F (in generations) listed below.

SHP.Khumbu	0								
SHP.Zhangmu	75.03517452	0							
TBN.Chamdo	175.1021587	152.9905392	0						
TBN.Lhasa	99.4091899	84.28159419	127.451659	0					
TBN.Nyingchi	178.0916603	154.1324178	175.6156492	128.6983106	0				
TBN.Qinghai	109.7551039	90.72805593	121.4626893	62.29403727	124.5871285	0			
TBN.Shannan	155.4501294	141.7797115	183.7515094	118.5173963	181.9520458	132.1023824	0		
TBN.Shigatse	88.52221759	74.83595547	129.5063096	52.08545169	130.9166838	63.4616983	111.2678317	0	
Han	182.9228989	162.6991631	194.4988604	144.6842509	201.896477	124.782149	211.6406732	146.2082403	0
	SHP.Khumbu	SHP.Zhangmu	TBN.Chamdo	TBN.Lhasa	TBN.Nyingchi	TBN.Qinghai	TBN.Shannan	TBN.Shigatse	Han

Table S5. Summary of divergence times between Sherpas, Tibetan and HAN estimated by MSMC, F_{ST}, and G-PhoCS. The upper boundary and lower boundary of divergence times estimated by MSMC indicate the start and end point of the population divergence. The time ranges estimated by F_{ST} indicate the divergence times across different highlander subgroups. Since the divergence events in G-PhoCS model are "average genomic divergence", one time point was given for each divergence event.

Divergence	MSMC	F_{ST} and N_e	G-PhoCS
Nepalese SHP and Chinese SHP	1,240 ~ 7,800	1,875	1,500
SHP and TBN	3,200 ~ 11,300<br>(Chinese SHP and TBN)	1,850 ~ 3,850<br>(Chinese SHP and TBN)	5,100
	6,100 ~ 13,300<br>(Nepalese SHP and TBN)	2,200 ~4450<br>(Nepalese SHP and TBN)	
Highlanders and HAN	(7,900 ~ 18,500)<br>Chinese SHP and HAN	4,050<br>(Chinese SHP and HAN)	6,100
	9,500 ~ 18,000<br>(Nepalese SHP and HAN)	4,575<br>(Nepalese SHP and HAN)	
	6,200 ~ 15,800<br>(Tibetan and HAN)	3,125 ~ 5,300<br>(Tibetan and HAN)	

Table S6 Summary of the 66 missense variants with their derived allele frequencies (DAF) rechecked by enlarging sample size of both SHP and TBN in target-genotyping panel. We identified 68 missense variants show extremely high DAF in SHP (DAF_{SHP}) but low in TBN (DAF_{TBN}) and HAN(DAF_{HAN}) by analyzing our generated NGS data. We rechecked DAF in the 68 variant by enlarging sample size of both SHP (DAF_{SHP*}) and TBN(DAF_{TBN*}) in Target-genotyping Panel (4 were failed when genotyping). We found that the previously identified variants show relative smaller population differentiation in their rechecked DAFs than that in DAFs of sequence data (Table S4). Despite that, however, the AF of each site in sequence panel present a liner correlation with that in target-genotyping panel (p = 0.02), indicating that though not obviously, the differentiations exist in the candidate sites. Also, the DAF of two variants (one in *TMEM247* near EPAS1 region and one in *EGLN1*) were also rechecked to validate whether TBN and SHP show similar DAF or not in these functional variants. Thus, information of 66 variants were listed. Notably, we selected 12 putatively functional variants which may contribute to the adaptation of SHP and TBN (Table 2). The physical positions of each sites follow GRCh37.

chrom	pos	rsid	Ref	Alt	Ances	DAF_{SHPseq}	DAF_{TBNseq}	DAF_{HANseq}	$F_{ST(SHPseq,TBNseq)}$	DAF_{TBN*}	DAF_{SHP*}	$DAF_{SHPseq2}$	DAF_ESA	DAF_SAS	DAF_AFR	DAF_EUR	DAF_AMR	CADD	GERP	Gene
8	107691513	rs28921397	A	G	A	0.5	0.0000	0.0000	0.78	0.0172	0.0789	0.0000	0.0029	0.0000	0.0000	0.0000	0.0000	31	5.96	OXR1
8	108264111	NA	G	A	G	0.5	0.0152	0.0000	0.69	0.0086	0.0724	0.0000	-	-	-	-	-	31	5.9	ANGPT1
6	42933464	rs35830695	G	A	G	0.5	0.0152	0.0513	0.69	0.0129	0.0724	0.0000	0.0404	0.0653	0.1387	0.0586	0.0474	31	5.76	PEX6
17	19645417	NA	T	A	T	0.4	0.0000	0.0000	0.71	0.0000	0.0987	0.0000	-	-	-	-	-	31	4.49	ALDH3A1
16	31099000	rs201075024	C	T	C	0.4	0.0000	0.0000	0.71	0.0043	0.1600	0.0000	0.0021	0.5408	0.0000	0.0020	0.0000	20.7	5.01	RP11-196G11.1
14	92981606	rs55930890	C	G	C	0.5	0.0303	0.1026	0.64	0.0647	0.1316	0.2500	0.1470	0.3344	0.1294	0.3140	0.1942	5.811	-2.58	RIN3
1	18808526	rs2992752	A	C	C	0.5	0.0303	0.0897	0.64	0.1034	0.1711	0.0000	0.1063	0.5139	0.3393	0.3887	0.2638	0.002	0.69	KLHDC7A
1	18808292	rs2992753	C	A	A	0.5	0.0303	0.0897	0.64	0.1034	0.1711	0.0000	0.1053	0.5130	0.3400	0.3887	0.2638	12.51	4.92	KLHDC7A
1	18807897	rs2992755	C	G	G	0.5	0.0303	0.0897	0.64	0.1078	0.1711	0.0000	0.1054	0.5128	0.4310	0.3887	0.2768	6.404	1.18	KLHDC7A
19	6751293	rs1049232	T	G	T	0.8	0.1364	0.2180	0.62	0.1983	0.2829	0.0000	0.3121	0.1290	0.3057	0.1816	0.2694	14.02	1.32	TRIP10
19	6751281	rs1049230	C	T	C	0.8	0.1364	0.2180	0.62	0.1983	0.2829	0.0000	0.3121	0.1280	0.3057	0.1816	0.2670	11.83	-0.0616	TRIP10
19	6751279	rs1049229	A	G	G	0.2	0.8636	0.7821	0.62	0.8017	0.7200	1.0000	0.6879	0.8700	0.6943	0.8184	0.7331	10.84	1	TRIP10
4	15964670	rs35496730	C	T	C	0.4	0.0152	0.0513	0.58	0.0517	0.1513	0.2500	0.0854	0.1734	0.0775	0.1536	0.1271	1.662	-2.08	FGFBP2
17	48712705	rs12604031	G	A	A	0.4	0.0152	0.0385	0.58	0.0388	0.1053	0.0000	0.0861	0.2462	0.2072	0.4084	0.2394	4.034	-2.7	ABCC3
9	131812201	rs17452596	G	A	G	0.3	0.0000	0.0385	0.60	0.0043	0.0921	0.0000	0.0238	0.0012	0.0000	0.0000	0.0000	9.189	-1.76	FAM73B
7	88965150	rs74564278	A	G	A	0.3	0.0000	0.0000	0.60	0.0043	0.0658	0.0000	0.0071	0.0906	0.0905	0.0097	0.0193	2.772	-1.59	ZNF804B
7	88964061	rs80006813	A	C	A	0.3	0.0000	0.0000	0.60	0.0043	0.0658	0.0000	0.0082	0.0906	0.0694	0.0097	0.0167	7.288	4.09	ZNF804B
3	196921405	rs527829647	A	G	A	0.3	0.0000	0.0000	0.60	0.0129	0.0789	0.0000	0.0011	0.0000	0.0000	0.0000	0.0000	22.3	5.17	DLG1
21	45509771	rs146810291	G	A	G	0.3	0.0000	0.0000	0.60	0.0000	0.0724	0.2500	0.0000	0.0010	0.0000	0.0000	0.0000	8.928	1.6	TRAPPC10
2	27529154	rs142922734	G	C	G	0.3	0.0000	0.0000	0.60	0.0000	0.0000	0.0000	0.0000	0.0000	0.0007	0.0030	0.0000	17.08	4.44	TRIM54
16	2147463	rs545053536	G	A	g	0.7	1.0000	0.9872	0.60	0.0000	0.0197	0.0000	0.0000	0.0010	0.0000	0.0000	0.0000	16.18	4.44	PKD1
12	117768315	rs549340789	G	A	G	0.3	0.0000	0.0000	0.60	0.0086	0.0855	0.0000	0.0022	0.0000	0.0000	0.0000	0.0000	17.79	4.74	NOS1
10	94695617	rs1326331	C	T	T	0.3	0.0000	0.0000	0.60	0.0043	0.0395	0.0000	0.0040	0.1221	0.0084	0.3173	0.1465	21.7	5.75	EXOC6
6	90402482	rs9294445	G	A	G	0.5	0.9546	0.8590	0.60	0.8879	0.8224	0.2500	0.9077	0.3801	0.5998	0.4590	0.7031	0.002	3.31	MDN1
6	90390443	rs9353689	C	A	C	0.5	0.9546	0.8590	0.60	0.8879	0.8267	0.2500	0.9077	0.3862	0.6141	0.4705	0.7089	7.377	-0.328	MDN1
7	1733192	rs56130225	C	T	c	0.6	0.9697	0.9359	0.60	0.0517	0.0658	0.0000	0.0612	0.1043	0.0623	0.1085	0.0660	0.526	-2.15	AC074389.6
7	1733182	rs79496824	C	T	C	0.4	0.0303	0.0641	0.56	0.0517	0.0658	0.0000	0.0612	0.1063	0.2014	0.1094	0.0799	1.412	-3.12	AC074389.6
6	43100537	rs78422682	T	G	T	0.4	0.0303	0.0000	0.56	0.0129	0.0395	0.0000	0.0107	0.0893	0.0955	0.0813	0.0593	6.252	1.52	PTK7
3	50332697	rs13100173	G	A	G	0.4	0.0303	0.0897	0.53	0.0517	0.1513	0.0000	0.0853	0.2569	0.1335	0.4621	0.3427	7.308	1.65	HYAL3
14	104642520	rs117428385	G	A	G	0.4	0.0303	0.0128	0.53	0.0259	0.1579	0.0000	0.0175	0.0023	0.0000	0.0000	0.0000	4.874	1.7	KIF26A
11	64597506	rs3741395	T	C	T	0.4	0.0303	0.0385	0.53	0.0431	0.1118	0.0000	0.0480	0.3968	0.2438	0.5765	0.3475	10.71	-0.416	CDC42BPG
10	45959385	rs12769244	G	A	A	0.6	0.9697	0.9744	0.53	0.9095	0.8026	0.7500	0.9472	0.9774	0.9418	0.9213	0.9682	21.2	5.97	MARCH8
10	45956828	rs3764990	G	A	G	0.4	0.0303	0.0256	0.53	0.0905	0.1974	0.2500	0.0528	0.0226	0.0143	0.0787	0.0293	18.84	5.65	MARCH8
1	158687896	rs857705	C	T	c	0.6	0.9697	0.8974	0.53	0.0431	0.0724	0.0000	0.1518	0.2536	0.2913	0.2236	0.1560	0.014	-2.94	OR6K3
6	90315789	rs3748085	A	G	G	0.5	0.0606	0.1154	0.53	0.1164	0.1711	0.7500	0.0931	0.3245	0.3154	0.1680	0.1302	20.1	4.07	ANKRD6
19	56249615	rs80009430	G	C	G	0.5	0.0606	0.1154	0.53	0.1250	0.1645	0.5000	0.1454	0.0402	0.0224	0.0050	0.0067	11.52	-3.22	NLRP9
13	115047305	rs3752105	G	A	g	0.4	0.8939	0.9359	0.53	0.1164	0.2171	0.2500	0.1023	0.3338	0.5362	0.2562	0.1640	11.12	0.383	UPF3A
17	18221010	rs12449313	A	G	A	0.9	0.2727	0.4103	0.52	0.3448	0.4145	0.2500	0.3197	0.1511	0.1474	0.2431	0.2404	1.232	-7.65	SMCR8
17	18148485	rs7498	G	A	A	0.1	0.7273	0.5769	0.52	0.6595	0.5855	0.7500	0.6785	0.8499	0.7118	0.7563	0.7392	18.79	-7.05	FLII
17	18137141	rs2290505	A	G	G	0.9	0.2727	0.4231	0.49	0.3405	0.4145	0.2500	0.3225	0.1521	0.1534	0.2392	0.2502	0.02	2.63	LLGL1
16	11608299	rs7205130	G	A	A	0.2	0.7879	0.6154	0.50	0.7609	0.5789	1.0000	0.6712	0.6276	0.4723	0.3744	0.3097	0.063	3.21	CTD-3088G3.8
16	11608244	rs7189101	T	A	T	0.8	0.2121	0.3846	0.50	0.2391	0.4276	0.0000	0.3287	0.3714	0.4388	0.6256	0.6864	0.498	-2.03	CTD-3088G3.8
1	68603586	rs983034	C	T	C	0.8	0.2121	0.1026	0.50	0.2284	0.3092	0.5000	0.1412	0.3201	0.1098	0.3534	0.3395	21.1	4.55	WLS

9	32526146	rs10813831	G	A	G	0.3	0.0152	0.0256	0.49	0.0216	0.0526	0.0000	0.1070	0.1528	0.2077	0.2773	0.1589	7.505	-2.08	DDX58
7	21948010	rs200891942	A	G	A	0.3	0.0152	0.0000	0.49	0.0129	0.1053	0.0000	0.0010	0.0000	0.0000	0.0000	0.0000	21.1	5.95	CDCA7L
6	57055354	rs1040461	C	T	T	0.7	0.9849	0.8974	0.49	0.9483	0.9013	1.0000	0.9106	0.9316	0.8013	0.9052	0.9043	21.8	5.79	RAB23
6	56919443	rs61740375	A	G	a	0.7	0.9849	0.8846	0.44	0.0517	0.0987	0.0000	0.0904	0.0684	0.1134	0.0948	0.0892	10.25	2.04	KIAA1586
4	84205995	rs150145464	A	C	a	0.7	0.9849	0.9872	0.44	0.0302	0.0526	0.0000	0.0136	0.0010	0.0000	0.0000	0.0000	1.964	-2.76	COQ2
4	39245910	rs138364911	A	G	A	0.3	0.0152	0.0000	0.44	0.0302	0.0461	0.0000	0.0019	0.0023	0.0000	0.0009	0.0000	19.04	3.8	WDR19
3	148597612	rs12489516	C	T	T	0.3	0.0152	0.0641	0.44	0.0819	0.1053	0.2500	0.0505	0.0331	0.0035	0.0409	0.0421	21.1	4.77	CPA3
3	100539943	rs190140224	G	A	G	0.3	0.0152	0.0128	0.44	0.0259	0.0987	0.0000	0.0071	0.0000	0.0000	0.0000	0.0000	5.854	-5.03	ABI3BP
2	37232879	rs2302657	A	C	A	0.3	0.0152	0.0128	0.44	0.0086	0.1067	0.2500	0.0325	0.0023	0.0000	0.0000	0.0000	16.87	5.63	HEATR5B
2	109545691	rs61761321	T	C	T	0.3	0.0152	0.1795	0.44	0.0345	0.1447	0.0000	0.0910	0.0023	0.0012	0.0000	0.0000	10.27	4.09	EDAR
19	3193327	rs117922207	G	A	G	0.3	0.0152	0.0000	0.44	0.0043	0.0526	0.0000	0.0058	0.0000	0.0000	0.0000	0.0000	18.29	4.17	NCLN
18	6977844	rs671871	A	G	G	0.7	0.9849	0.9359	0.44	0.9181	0.8882	1.0000	0.9116	0.8669	0.3707	0.8280	0.7992	20.6	2.15	LAMA1
17	64222164	rs1801692	C	T	C	0.3	0.0152	0.0385	0.44	0.0302	0.1118	0.0000	0.0330	0.0470	0.0109	0.0391	0.0187	0.002	-2.29	APOH
17	4348459	rs200883298	C	G	C	0.3	0.0152	0.0000	0.44	0.0086	0.0724	0.0000	0.0021	0.0000	0.0000	0.0000	0.0000	0.036	-2.68	SPNS3
16	3304739	rs11466018	A	G	A	0.3	0.0152	0.0513	0.44	0.0172	0.0267	0.0000	0.0579	0.0000	0.0000	0.0000	0.0000	0.012	-1.51	MEFV
15	65489128	rs938952	C	T	T	0.3	0.0152	0.0000	0.44	0.0259	0.0592	0.0000	0.0081	0.1170	0.4205	0.1982	0.1222	4.554	3.46	CILP
14	22294137	rs34740210	G	A	G	0.3	0.0152	0.0256	0.44	0.0086	0.0329	0.2500	0.0569	0.0000	0.0007	0.0000	0.0000	6.065	-0.432	TRAV10
12	93195435	rs73362428	T	A	T	0.3	0.0152	0.0641	0.44	0.0783	0.0667	0.0000	0.0375	0.0184	0.0365	0.0124	0.0104	0.545	1.81	EEA1
1	91405998	rs149597385	C	T	C	0.3	0.0152	0.0000	0.44	0.0259	0.1184	0.0000	0.0100	0.0012	0.0000	0.0000	0.0000	32	4.63	ZNF644
1	156551628	rs116035113	G	T	G	0.3	0.0152	0.0000	0.44	0.0474	0.1250	0.0000	0.0030	0.0010	0.0475	0.0125	0.0124	23.9	4.83	TTC24
2	46707674	rs116983452	C	T	C	0.4	0.2121	0.9744	0.1017	0.7241	0.6389	1.0000	0.0207	0.0035	0.0000	0.0000	0.0000	11.18	2.97	TMEM247
1	231557623	rs186996510	G	C	G	0.1	0.5910	0.0385	0.52	0.5500	0.4800	0.25	0.0100	0.0020	0.0000	0.0000	0.0020	14.73	3.51	*EGLN1*

References

1. Xu S, *et al.* (2011) A genome-wide search for signals of high-altitude adaptation in Tibetans. *Mol Biol Evol* 28(2):1003-1011.
2. Simonson TS, *et al.* (2010) Genetic Evidence for High-Altitude Adaptation in Tibet. *Science* 329(5987):72-75.
3. Peng Y, *et al.* (2011) Genetic variations in Tibetan populations and high-altitude adaptation at the Himalayas. *Mol Biol Evol* 28(2):1075-1081.
